# Adaptation to *ex vivo* culture reduces human hematopoietic stem cell activity independently of cell cycle

**DOI:** 10.1182/blood.2023021426

**Published:** 2024-05-28

**Authors:** Carys S. Johnson, Matthew Williams, Kendig Sham, Serena Belluschi, Wenjuan Ma, Xiaonan Wang, Winnie W. Y. Lau, Kerstin B. Kaufmann, Gabriela Krivdova, Emily F. Calderbank, Nicole Mende, Jessica McLeod, Giovanna Mantica, Juan Li, Charlotte Grey-Wilson, Michael Drakopoulos, Shaaezmeen Basheer, Shubhankar Sinha, Evangelia Diamanti, Christina Basford, Nicola K. Wilson, Steven J. Howe, John E. Dick, Berthold Göttgens, Anthony R. Green, Natalie Francis, Elisa Laurenti

**Affiliations:** 1 Wellcome and Medical Research Council Cambridge Stem Cell Institute, University of Cambridge, Cambridge, United Kingdom; 2 Department of Haematology, University of Cambridge, Cambridge, United Kingdom; 3 Cell Process Development, Cell & Gene Therapy, GlaxoSmithKline, Stevenage, United Kingdom; 4 Princess Margaret Cancer Center, University Health Network, Toronto, Canada; 5 Department of Gene Therapy and Regenerative Medicine, King’s College London, London, United Kingdom

## Abstract

Loss of long-term hematopoietic stem cell (LT-HSC) function *ex vivo* hampers the success of clinical protocols reliant on culture. However, the kinetics and mechanisms by which this occurs remain incompletely characterized. Here, through time-resolved scRNA-Seq, matched *in vivo* functional analysis and the use of a reversible *in vitro* system of early G_1_ arrest, we define the sequence of transcriptional and functional events occurring during the first *ex vivo* division of human LT-HSCs. We demonstrate that the sharpest loss of LT-HSC repopulation capacity happens early on, between 6 and 24 hours of culture, before LT-HSCs commit to cell cycle progression. During this time window, LT-HSCs adapt to the culture environment, limiting global variability in gene expression and transiently upregulating gene networks involved in signaling and stress responses. From 24 hours, LT-HSC progression past early G_1_ contributes to the establishment of differentiation programmes in culture. However, contrary to current assumptions, we demonstrate that loss of HSC function *ex vivo* is independent of cell cycle progression. Finally, we show that targeting LT-HSC adaptation to culture by inhibiting early activation of JAK/STAT signaling improves HSC long-term repopulating function *ex vivo*. Collectively, our study demonstrates that controlling early LT-HSC adaptation to *ex vivo* culture, for example via JAK inhibition, is of critical importance to improve HSC gene therapy and expansion protocols.

## Introduction

A trillion blood cells are produced daily in humans. This impressive output is achieved by rare hematopoietic stem cells (HSCs) with the unique capacity to drive production of all blood cell types (differentiation), whilst maintaining a functional HSC pool (self-renewal). Owing to this extensive regenerative capacity, HSCs are the key functional units of HSC transplantation and HSC gene therapy (GT). HSC GT promises to be a curative, one-time treatment, and is under clinical investigation to treat more than 10 monogenic diseases^1^. HSC GT currently requires an *ex vivo* culture step, which strongly reduces HSC functionality^2,3^. Minimising HSC attrition during *ex vivo* protocols would provide patients with larger numbers of functional HSCs, improve treatment efficacy and safety in current HSC GT indications, and pave the way for new therapeutic applications.

Long-term blood formation post-transplantation is driven by predominantly quiescent long-term HSCs (LT-HSCs)^4,5^. Quiescence (G_0_) is defined as the reversible absence of cell cycling. G_0_ is characterized by decreased cell size, reduced protein biosynthesis^6,7^, high autophagic recycling^8,9^, an elevated basal expression of cell stress response pathways^10–13^, a relatively inactive glycolytic metabolism^14,15^, and is maintained *in vivo* by the hypoxic bone marrow niche^16,17^. Upon stimulation, HSCs exit quiescence (G_0_ – early G_1_) and progressively phosphorylate Retinoblastoma (Rb) until the restriction point, where commitment to division occurs and cell cycle progression (late G_1_–S–G_2_–M) ensues. CDK6 is a master regulator of HSC quiescence exit kinetics, whose differential regulation within the HSC pool guarantees HSC long-term maintenance^18^. An emerging body of work has demonstrated further heterogeneity within the quiescent state of LT-HSCs^4^ that is associated with distinct lineage preferences and kinetics of reconstitution post-transplantation^19–22^. Clinical GT protocols target the CD34^+^ fraction (a heterogeneous mix of HSCs and progenitor cells – HSPCs), with a limited understanding of how *ex vivo* protocols impact the underlying biology unique to the LT-HSC population.

In current culture systems, HSCs inevitably exit quiescence and divide. *Ex vivo* culture increases protein synthesis rates^6,23^, remodels the mitochondria to an activated state^24^ with increased oxidative metabolism and ROS production^25^, disrupts optimal proteostasis programs^23,26^ and reduces dependency on lysosomal recycling^27^. These changes are all associated with a net decline in long-term repopulation capacity. Early studies showed that it is exclusively HSPCs in G_0_ before^28^ and following culture^29,30^ that engraft, but not their cycling counterparts. From this and extensive literature from *in vivo* models^31–33^, it has been assumed that cell cycle progression itself drives loss of HSC function in culture. However, to date no study has formally addressed this question in human LT-HSCs undergoing clinically relevant culture. Despite recent advances in strategies to expand HSCs *ex vivo*^34^ and the increased clinical adoption of HSC GT, we are still lacking a kinetic and mechanistic understanding of how HSCs lose self-renewal *ex vivo*. In this study, by pairing scRNA-Seq with *in vivo* functional analysis in a time-resolved manner, we dissect the transcriptional and functional changes occurring in highly purified human LT-HSCs over their first division in culture.

## Methods

### Human samples

Human biological samples were sourced ethically and their research was used in accord with the terms of the informed consents under an IRB/RC approved protocol as specified below. Primary cord blood (CB) samples were obtained with informed consent from healthy donors by the Cambridge Blood and Stem Cell Biobank. CBs of both sexes were processed as a single sample, except for scRNA-seq where only single sex CB pools were used. Mobilized peripheral blood (mPB) was obtained from healthy male donors aged 25-28 by administration of daily Filgrastim (Neupogen) (10 μg/kg per day) for 5 days. Apheresis was performed on day 5 and 6 using the Optia Spectra (Terumo BCT). CB collection and experimental work on CB and mPB CD34^+^ cells were performed in accordance with regulated procedures approved under the 07/MRE05/44 and 18/EE/0199 REC research studies. Sample preparation, flow cell sorting and phenotyping are reported in Supplemental Methods.

### *Ex vivo* culture

Two complementary culture systems were used: 1) “EXPER” conditions: StemPro base media (ThermoFisher Scientific) supplemented with Nutrients (0.028%) (ThermoFisher Scientific), Pen/Strep (1%), L-Glu (1%), human LDL (50ng/ml) (Stem Cell Technologies) and the following cytokines: SCF (100ng/ml), Flt-3L (20ng/ml), TPO (100ng/ml), EPO (3 units/ml), IL-6 (50ng/ml), IL-3 (10ng/ml) and GM-CSF (20ng/ml). 2) “GT” conditions: GMP SCGM (CellGenix) supplemented with L-Glutamine (1%) (Thermo Fisher Scientific), Pen/Strep (1%) (ThermoFisher Scientific) and the following cytokines: SCF (300ng/ml), Flt-3L (300ng/ml), IL-3 (60ng/ml) and TPO (100ng/ml). All cytokines from Miltenyi/Peprotech except EPO (Janssen). The CDK4/CDK6 inhibitor Palbociclib (PD; PD0332991, Sigma, 200nM), the JAK1/JAK2 inhibitor Ruxolitinib (RUX; Selleckchem, 5-500nM), UM171 (Stem Cell Technologies, 35nM) or Z-VAD(OH)-FMK (Cayman Chemical, 100nM) were added to GT, EXPER or Myeloid-Erythroid-Megakaryocytic generating “MEM” media where indicated. Methodology for lentiviral vector production and all *in vitro* assays to assess HSC function, cell cycle, differentiation, and serial replating ability are described in Supplemental Methods.

### Xenograft transplantation

Animal studies were ethically reviewed by the University of Cambridge Animal Welfare and Ethical Review Body (AWERB) and carried out in accordance with Animals (Scientific Procedures) Act 1986, the GSK Policy on the Care, Welfare and Treatment of Animals and institutional guidelines approved by the University Health Network Animal Care Committee. NOD.Cg-PrkdcscidIl2rgtm1Wjl/SzJ (NSG) mice or NOD.Cg-Prkdcscid Il2rgtm1Wjl Tg(CMV-IL3,CSF2,KITLG)1Eav/ MloySzJ (NSG-SGM3) mice were bred in-house or obtained from Charles River. All animals were housed in a specific pathogen free animal facility and experiments were conducted under UK Home Office regulations or in accordance with institutional guidelines approved by the UHN Animal care. Primary and secondary transplantation methods and analysis criteria are reported in Supplemental Methods.

### Single cell RNA-Sequencing

scRNA-Seq libraries were prepared using the Smart-Seq2 protocol^35^, adapted as described in Supplemental Methods. Libraries were quantified using the KAPA library quantification kit (Roche) and were sequenced by paired end sequencing (150bp) using Illumina HiSeq4000 (Illumina) and NovaSeq 6000 (Illumina) at the CRUK-CI genomics core facility (Cambridge, UK). scRNA-Seq experimental design and bioinformatic methods are described in Supplemental Methods.

### Statistical Analysis

Appropriate statistical tests were performed using Graphpad Prism (v9.3), R Studio (v1.2) and Python (v3.8.6), further described in Supplemental Methods.

## Results

### Loss of HSC function occurs early during *ex vivo* culture

HSC functional attrition in culture severely reduces the efficacy of HSC clinical approaches like *ex vivo* GT. We therefore sought to study the kinetics of this phenomenon using: i) purified human LT-HSCs (CD34^+^CD19^-^CD38^-^CD45RA^-^CD90^+^CD49f^+ 4^), ii) early culture time-points (≤ 72h) corresponding to key HSC cell cycle transitions^18^, iii) two distinct *ex vivo* systems and HSC sources: “GT_mPB conditions” using mPB LT-HSCs and two hits of transduction with a lentiviral vector (LV) expressing GFP ^2,36,37^; “EXPER_CB conditions” using CB LT-HSCs (unless otherwise indicated) and promoting differentiation ^18^.

**Fig. 1:**
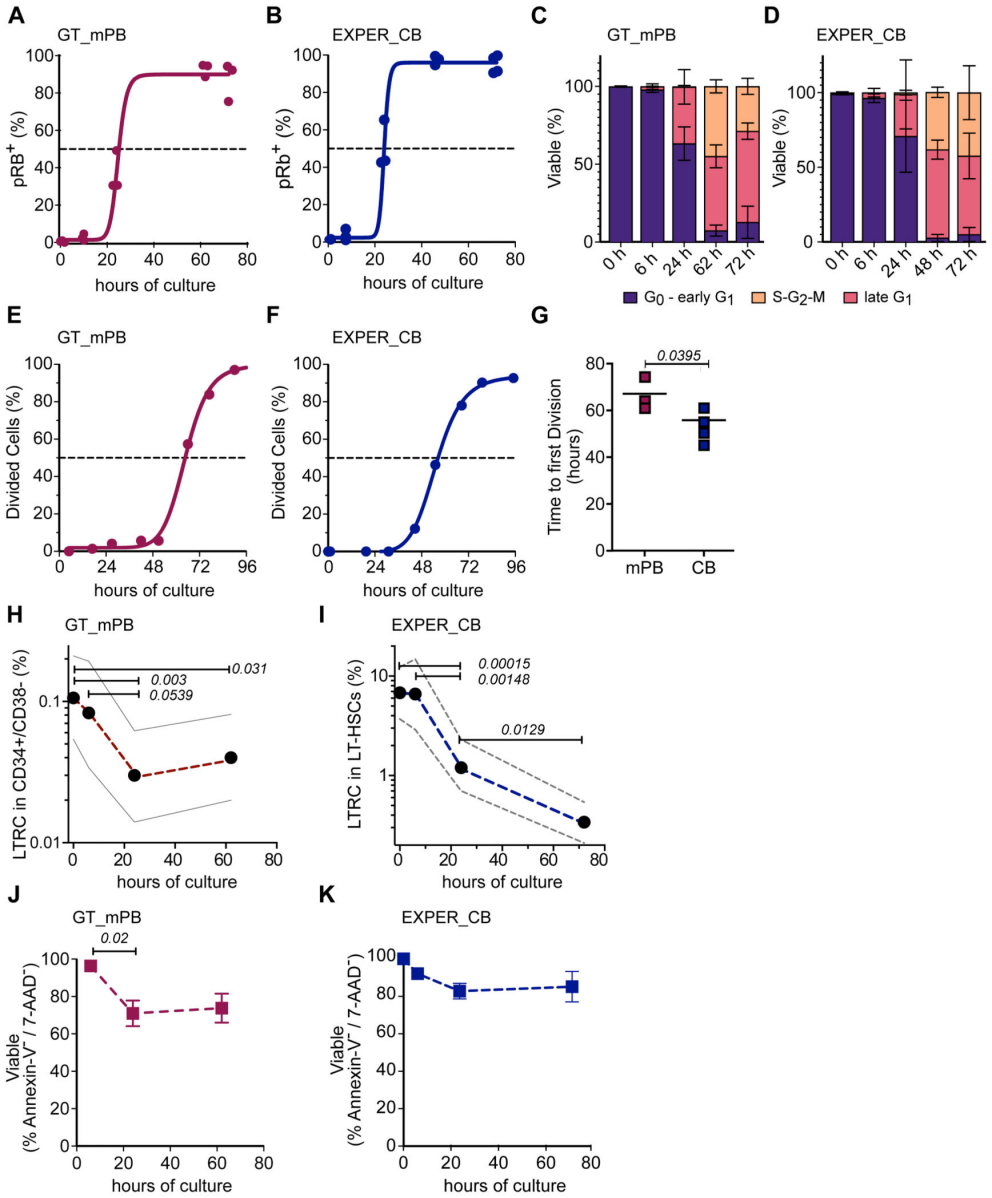
Kinetics of cell cycle progression, survival and loss of long-term repopulation capacity of LT-HSCs during *ex vivo* culture. **(A)** Cumulative quiescence exit kinetics of GT_mPB LT-HSCs determined by phospho Rb (Ser 807 – 811) flow cytometry analysis. Curve is least-squares sigmoidal fit with EC_50_ = 24.7 h; n=3 biological replicates for 0h, 24h, 62h, 72h and n=4 biological replicates for 6h. Standard error ± 5.186; R^2^ = 0.9797. **(B)** Cumulative quiescence exit kinetics of EXPER_CB LT-HSCs determined by phospho Rb (Ser 807 – 811) flow cytometry analysis. Curve is least-squares sigmoidal fit with EC_50_ = 24.72h; n=3 biological replicates for 0h, 6h, 24h, 48h and n=4 biological replicates for 72h. Standard error ± 3.944; R^2^ = 0.9844. **(A-B)** Dashed line indicates EC_50,_ time of quiescence exit. **(C)** Cell cycle phase assignment of GT_mPB LT-HSCs determined by pRb/DAPI flow cytometry analysis. Equivalent repeats as in (**A**) **(D)** Cell cycle phase assignment of EXPER_CB LT-HSCs determined by pRb/DAPI flow cytometry analysis. Equivalent repeats as in (**B**). **(E)** Cumulative first division kinetics (excluding dead cells) of GT_mPB LT-HSCs. Curve is least squares sigmoidal fit. Representative examples shown (biological replicates: n=3). Dashed line indicates EC_50_. EC_50_ = 64.83 h; 95% CI = 62.83 h – 66.87 h; R^2^ = 0.9976. **(F)** Cumulative first division kinetics (excluding dead cells) of EXPER_CB LT-HSCs. Curve is least squares sigmoidal fit. Representative examples shown (biological replicates: n=4). EC_50_ = 55.44 h; 95% CI = 54.28 h – 56.70h; R^2^ = 0.9995. **(G)** Time to first division kinetics summary of LT-HSCs cultured in GT_mPB **(E)** and EXPER_CB **(F)** systems (biological replicates: n=3 for GT; n=4 for EXPER). Unpaired t-test shown. **(H)** Percent of LTRC in GT_mPB CD34^+^CD38^-^ cells as determined by LDA analysis in the transplanted population. %LTRCs at each time-point +/- 95% CI shown. LTRC frequency estimate for GT_mPB: 0h: 1 in 939 (29 mice); 6h: 1 in 1211 (21 mice); 24h: 1 in 3371 (23 mice); 62h: 1 in 2510 (23 mice). ELDA statistical test shown. Data in Table S1. **(I)** Percent of LTRC in LT-HSCs cultured in EXPER_CB system as determined by LDA analysis in the transplanted population. % LTRCs at each time-point +/- 95% CI shown. LTRC frequency estimate for EXPER_CB: 0h: 1 in 14.8 (31 mice); 6h: 1 in 15.2 (19 mice); 24h: 1 in 80.6 (31 mice); 72h: 1 in 293.7 (40 mice). ELDA statistical test shown. Data in Table S1. **(J)** Survival of LT-HSCs cultured in GT_mPB systems determined by Annexin-V/7-AAD flow cytometry. n=3 biological replicates at each time-point. Mean +/- SD shown. **(K)** Survival of LT-HSCs cultured in EXPER_CB system determined by Annexin-V/7-AAD flow cytometry. n=3 biological replicates at each time-point. Paired t-test shown for 6h and 24h comparison. Mean +/- SD shown.

We first characterized LT-HSC cell cycle kinetics in our *ex vivo* conditions. LT-HSCs in either GT or EXPER conditions progressed past late G_1_ at ~24h post culture initiation (Fig.1A-B) as assessed by both phosphorylation of Rb at the serine residue 807-811 and acquisition of the Ki-67 marker (Fig.S1A-B). Concurrently, the proportion of cycling cells (S-G_2_-M phases) increased within the 24-72h window (Fig.1C-D). LT-HSC time to first division was estimated at 62.0h ± 6.5 for GT_mPB conditions and 53.4h ± 6.5 for EXPER_CB conditions (Fig. 1E-G). Therefore, independently of HSC source and culture conditions, LT-HSCs progress to late G_1_ by ~24h and their first division by 72h.

Next, we quantified long-term repopulating cell frequency (%LTRC) at 0, 6, 24 and 62/72h of *ex vivo* culture, performing limiting dilution analysis (LDA) xenotransplantation in NSG mice. We transplanted fixed numbers of: i) GT_mPB CD34^+^ CD38^-^ cells cultured for 62h (time of infusion into patients in HSC GT protocols); ii) EXPER_CB LT-HSCs cultured for up to 72h. Human engraftment was analysed 18 weeks post-transplantation. Of note, in GT conditions, almost all engrafted animals displayed GFP^+^ cells (10/11; Table S1), indicating that transduced cells contribute to long-term engraftment. LDA showed that the %LTRC within the transplanted LT-HSC population was unchanged for the first 6h of culture. However, the %LTRC dropped conspicuously by 24h (GT: ~3-fold, p= *0.003*; EXPER: ~5-fold, *p=0.00015* compared to 0h). An additional decrease in the %LTRC was observed past 24h in EXPER conditions (~2-fold, *p=0.0129* compared to 24h), when LT-HSCs progress through the cell cycle (Fig.1H-I, Table S1-2). We next measured apoptosis via Annexin-V/7-AAD staining. A decrease in viable cells was observed between 6h and 24h with no further decrease at later time-points in both GT and EXPER culture systems (Fig.1J-K). Interestingly, the loss in LTRC observed between 6h and 24h *ex vivo* largely outnumbered the loss in viability measured in the same time window. We therefore conclude that HSC loss *ex vivo* occurs predominantly before cells enter the late G_1_ phase of the cell cycle.

### Transcriptome dynamics of LT-HSCs over the first cell cycle *ex vivo*

**Fig. 2:**
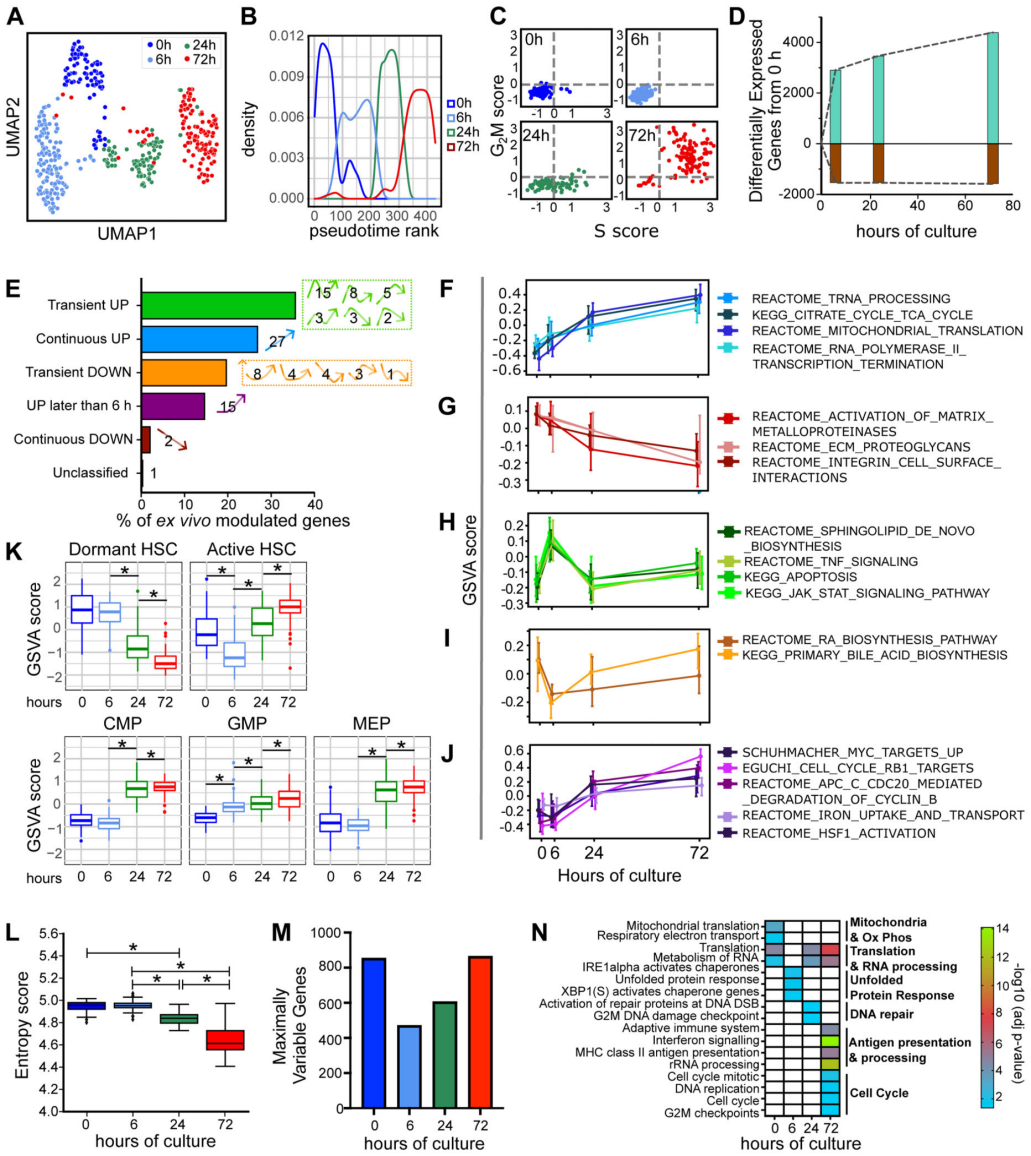
Dynamics of gene expression over the first division of LT-HSC *ex vivo* at single cell resolution. **(A)** UMAP of 429 single EXPER_CB LT-HSCs over a time-course of 0h, 6h, 24h and 72h (n=2 independent experiments). UMAP generated using Seurat 4 pipeline following cell cycle regression. **(B)** 2D pseudotime rank plot of EXPER_CB LT-HSCs over time-course generated following cell cycle regression. **(C)** Transcriptional allocation of cell cycle status at time-points of EXPER_CB LT-HSC culture; n= 429 single cells. **(D)** Number of differentially expressed genes (FDR<0.05) at each time-point with respect to 0h. Upregulated genes (green) and downregulated genes (brown). Full list of genes available in Table S4. **(E)** Broad patterns of gene expression identified over time-course (8,966 genes classified after filtering by DEG Report algorithm). Numbers indicate the percentage of genes showing the specific patterns of gene expression displayed to the right of bar. **(F-J)** GSVA score of c2 curated pathways showing specific expression patterns: **(F)** continuous up; **(G)** continuous down; **(H)** transient up; **(I)** transient down; **(J)** up later than 6h. GSVA score calculated per single cell with line at median and upper and lower whiskers indicate 25th and 75th percentile of expression. **(K)** GSVA scores of indicated published gene signatures, representative of specific HSPC subsets. Median and interquartile range shown. * indicates *p<0.001*. **(L)** scEntropy value at each time-point (calculated for both batches combined; Wilcoxon rank sum test shown; 0h vs 6h p = 0.835). Median and interquartile range shown. * indicates *p<0.001*. **(M)** Number of maximally variable genes at each time-point (MVG; see Supplemental Methods; 2792 genes total). **(N)** Selected biological pathways significantly enriched from MVG (-log_10_(adjusted p-value) *<0.05*). Full list of pathways available in Table S7.

To better understand the kinetics of LTRC loss *ex vivo*, we investigated the transcriptional changes associated with the first cell division of LT-HSCs in culture. CB_EXPER LT-HSCs were cultured for 0h, 6h, 24h and 72h before scRNA-seq via an adapted Smart-Seq2 protocol ^35^. 429 cells, collected over 2 independent experiments, passed quality control. Cells from both experiments were integrated via 2 distinct pipelines (Scanpy and Seurat 4; see Supplemental Methods), which yielded highly concordant results. LT-HSCs grouped by culture duration in UMAP visualisations, independently of the batch (Fig.2A, Fig.S2A), of whether cell cycle regression was applied (Fig.S2B) and of the integration pipeline used (Fig.S2C).

At 0h, we identified two transcriptionally defined subsets of LT-HSCs, in accordance with recent reports of transcriptional heterogeneity within the quiescent human LT-HSC fraction (Suppl. Note 1, Fig.S2D-H, Table S3). After 0h, pseudotime ordering largely recapitulated chronological time (Fig.2B, Fig. S1I) and transcriptional assignment of cell cycle status agreed with functional analysis (Fig.2C). LT-HSCs therefore progress through distinct transcriptional states over time in culture. DESeq2 differential gene expression for all pairwise comparisons in the dataset (Table S4) showed that *ex vivo* culture has an extensive and rapid effect on the transcriptome. 10,010 genes changed over the time-course (hereafter termed “*ex vivo* modulated genes”, Table S5). Interestingly, of gene expression changes occurring between 0h and 72h (5,980 genes), 75% (4,460 genes) were already observed at 6h (Fig.2D), before the vast majority (>90%) of cells have entered late G_1_ in these culture conditions (Fig.1A).

We then used degPatterns^38^ on *ex vivo* modulated genes to define gene (Fig. 2E, Fig.S3A, Table S6) and pathway (GSVA analysis^39^; Fig.S3B, Table S6) expression patterns over the time-course. Similar dynamic patterns of upregulation and downregulation were observed at the gene and pathway level (Fig. S3A-B). We found 5 expression patterns of interest, comprising: i) cumulative increases in expression of pathways linked to transcription, RNA splicing, translation and oxidative phosphorylation (Fig.2F); ii) progressive decreases of pathways linked to cell adhesion (Fig.2G); iii) early loss of the AP-1 transcription factors *JUN* and *FOS*, key to the HSC quiescence network (Fig.S3C); iv) transient patterns of gene (Fig.S3D) or pathway (Fig.2H-I) expression indicating that HSCs initiate a stress response to adapt to culture signals. Particularly abundant were genes/pathways upregulated from 0 to 6h and subsequently decreased at 24h (27.3 % of total genes; 76.3 % of “Transient UP genes”). These included sphingolipid *de novo* biosynthesis (i.e. *DEGS1)*, apoptosis (e.g. *CFLAR* and *BIRC2* genes) and stress pathways (e.g. *ATF4*); v) upregulation of expression after the 6h time-point of genes/genesets related to proliferation and differentiation (Fig.2J-K). These included *MYC* (Fig.S3E), MYC targets, cell cycle progression, signatures of active human LT-HSCs^22^ and committed progenitors^40^. Finally, we calculated scEntropy scores, which decrease during differentiation fate decisions^41^. scEntropy scores significantly decreased only after 6h (Fig.2L), supporting the concept that cell fate decisions occur at the 6 to 24h transition.

Dynamic changes in gene expression variability have been linked to cell fate decisions in haematopoietic progenitors^42^ and T cell activation^43^. We therefore investigated gene expression variability over the time-course, employing BASiCS ^44,45^ and determining genes with maximum variability (MVGs) at each time-point (Supplemental Methods, Fig.S2F). The number of MVGs was lowest at 6h (Fig.2M) showing that gene expression variability is restrained early upon culture initiation. This is likely attributable to adaptation of all LT-HSC subsets to the culture environment. MVGs at 6h were enriched for UPR regulatory genes (including *DNAJC3* and *XBP1;* Fig.2N, Table S7), indicating that despite a global restriction in gene expression variability, LT-HSCs vary in their degree of activation of cell stress response genes at this critical time-point.

In summary our results indicate that, within the first 24 hours, LT-HSCs initially adapt to culture by restricting global gene variability and activating a transient stress response. Subsequently, *MYC* and cell cycle genes are upregulated and differentiation programmes are initiated.

### Cell cycle dependent initiation of differentiation in cultured LT-HSCs

**Fig. 3:**
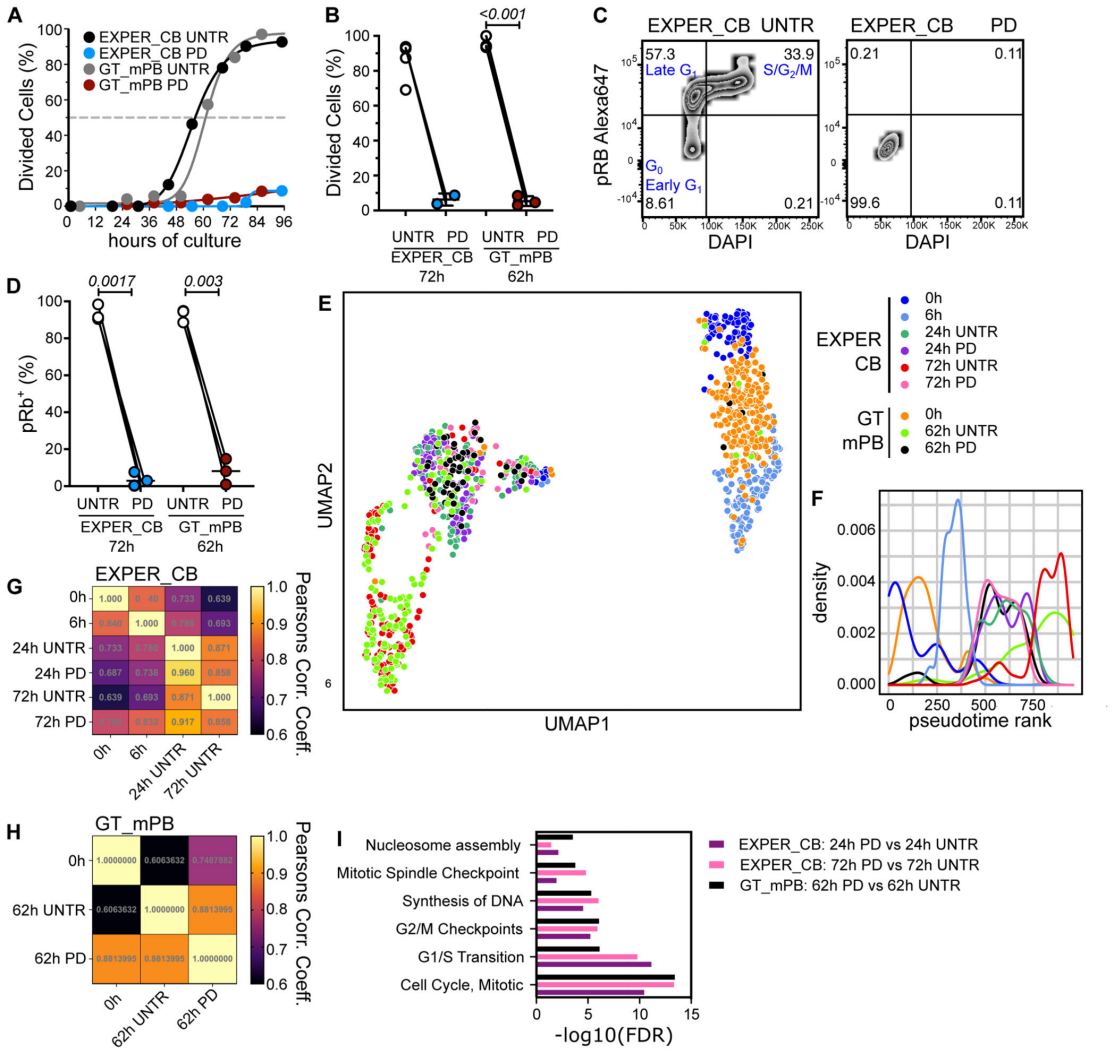
Transcriptional effects of preventing progression past early G_1_ during *ex vivo* culture of LT-HSCs. **(A)** Cumulative first division kinetics (excluding dead cells) of UNTR/PD treated LT-HSCs cultured in GT_mPB (grey and dark red) or EXPER_CB (black and blue) system. Curve is least squares sigmoidal fit. Representative example shown (n=3 UNTR/PD matched biological replicates for GT and n=2 UNTR/PD treated matched biological replicates for EXPER). Dashed line indicates EC_50_, time to first division. Untreated (UNTR) and Palbociclib treated 200nM (PD). **(B)** Divided single cells as a proportion of total alive cells at 96h (EXPER_CB: n=5 biological repeats, n=2 UNTR/PD matched biological repeats; GT_mPB: n=3 matched biological repeats). Paired t-test shown. **(C)** Representative example of flow cytometry plot for phospho Rb (Ser 807 – 811) Alexa 647 and DAPI staining on UNTR (left) or PD treated (right) LT-HSCs cultured in CB_EXPER medium for 72h. **(D)** Quantification of pRb^+^ (as % of viable cells) in UNTR/PD treated LT-HSCs cultured for 62h in GT_mPB (n=3 UNTR/PD treated matched biological repeats; no LV transduction) or 72h in EXPER_CB (n=3 UNTR/PD treated matched biological repeats) systems. Paired t-test shown. **(E)** UMAP visualisation of scRNA-seq from 954 LT-HSCs from the indicated culture conditions (EXPER_CB: 536 single cells: GT mPB: 418 single cells). Cell cycle regression applied. **(F)** 2D Pseudotime density rank plot of single cells shown in (**E**). Cell cycle regression applied. **(G)** Pearson’s correlation coefficient estimate comparing the median expression value of 10,903 genes at time-point/condition comparisons in the EXPER dataset (union of all differentially expressed genes between any 2 UNTR time-points and between PD treated and UNTR conditions; available in Table S5). **(H)** Pearson’s correlation coefficient estimate comparing the median expression value of 5,469 genes at time-point/condition comparisons in the GT dataset. (union of all differentially expressed genes between any 2 UNTR time-points and between PD treated and UNTR conditions; available in Table S5). **(I)** Selected Reactome pathways (FDR<0.05) enriched between PD treated and UNTR LT-HSCs cultured for matched durations of 24h in EXPER_CB (purple), 72h in EXPER_CB (pink) and 62h in GT_mPB (black) systems. Full DeSeq2 results and Reactome pathway enrichment available in Table S8.

E*x vivo*, changes to HSC metabolism and organelle biology occur concomitantly with cell cycle progression and loss of function ^23–25,27,46^. Our observation that the sharpest drop in LT-HSC repopulation capacity occurs before most LT-HSCs enter late G_1_ prompted us to formally test to what extent progression past early G_1_ contributes to HSC identity and function in culture. We took advantage of the CDK4/CDK6 inhibitor Palbociclib (PD033299; herein PD), previously established to prevent division of CB LT-HSCs ^18^. First, we repeated this finding with LT-HSCs cultured in both the GT_mPB and EXPER_CB systems (Fig.3A-B), showing early G_1_ arrest of CB (Fig.3C-D) and mPB LT-HSCs (Fig.S4A). PD treatment is reversible and does not compromise LT-HSC differentiation (Fig.S4B-E), therefore allowing assessment of the effect of culture in the absence of cell cycle progression.

To disentangle which transcriptional differences in culture depend on cell cycle progression, scRNA-Seq was performed on LT-HSCs treated with PD for 24 and 72h in the EXPER_CB system and 62h in the GT_mPB systems. These data were integrated with 0h and 6h cells in a unique embedding via Seurat4 (954 cells; n=6 independent experiments). As expected, PD treated LT-HSCs were transcriptionally allocated to G_1_ in both culture systems (Fig.S4F-G). Cultured LT-HSCs pharmacologically arrested in G_1_ localize with 24h UNTR LT-HSCs on the UMAP (Fig.3E) and by pseudotime (Fig.3F) independently of the cell source, culture system, duration of PD treatment and of whether cell cycle regression was applied or not (Fig.S4H, Fig. S4I). The impact of PD treatment on the transcriptome was relatively minimal, with gene expression correlations higher when comparing UNTR vs PD conditions (Pearson’s correlation coefficient; EXPER_CB 24h: 0.96; EXPER_CB 72h: 0.871; GT_mPB 62h:0.881) compared to any UNTR time-point comparisons (Fig.3G-H). Furthermore, most pathways significantly enriched by PD treatment either at 24h or 72h relate to cell cycle progression (Fig.3I, Table S8), demonstrating low off-target effects of PD.

**Fig. 4:**
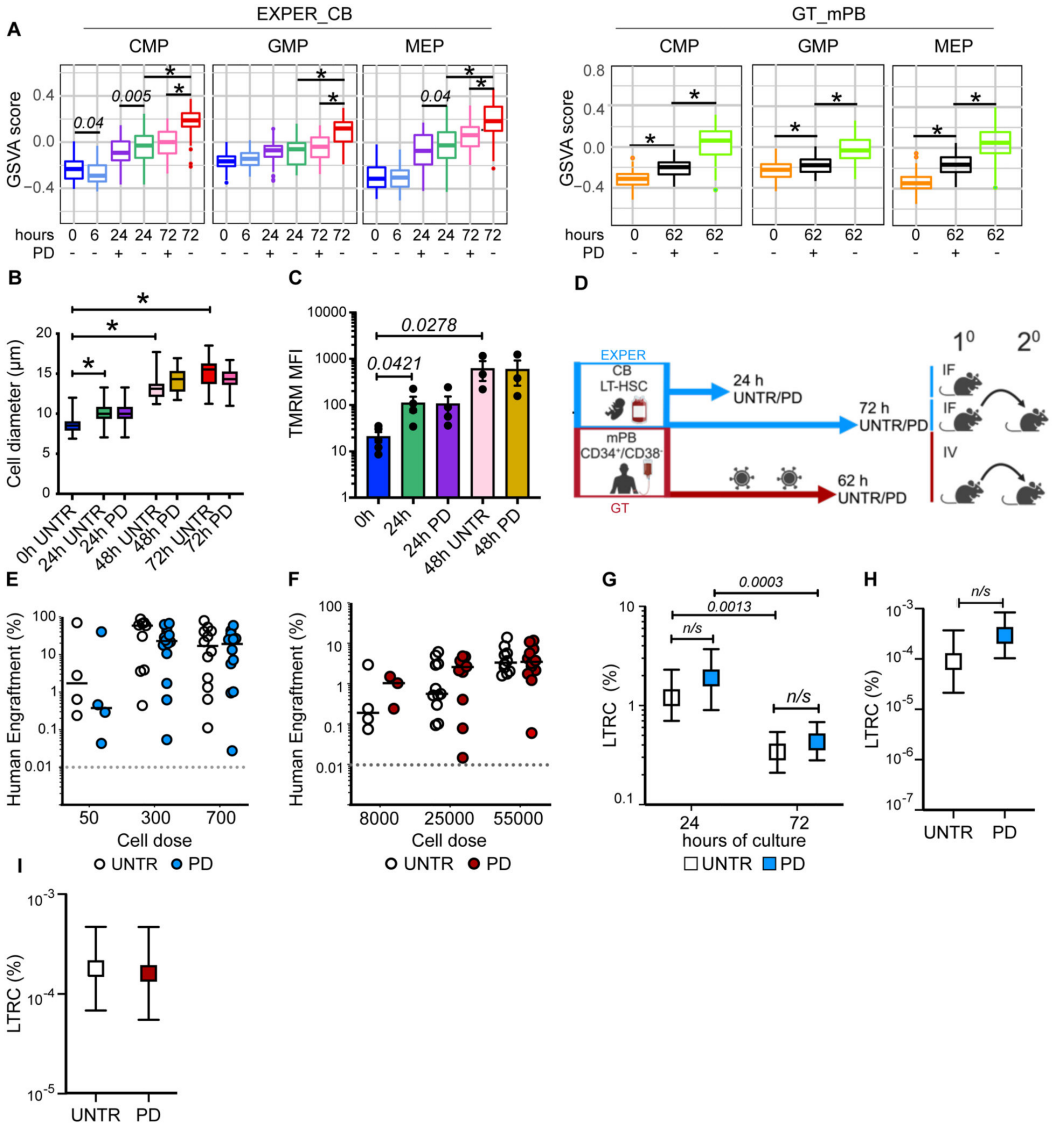
Preventing progression past early G_1_ during *ex vivo* culture of LT-HSCs dampens the establishment of differentiation programmes but does not affect loss of long-term repopulation capacity. **(A)** GSVA scores of indicated lineage gene expression signatures from ^40^ at indicated time-points of LT-HSC culture. GSVA score generated per cell and line at median. EXPER_CB : n= 536 single cells; GT_mPB: n=418 single cells. **(B)** Cell diameters of single CB LT-HSCs cultured in EXPER conditions (n=2 experiments representing n=469 total single cells). Unpaired t-test. * indicates *p<0.001*. **(C)** Tetramethylrhodamine (TMRM) staining of bulk CB LT-HSCs cultured in EXPER conditions (n=5 biological repeats for 0h; n = 4 matched biological repeats for UNTR/PD treated 24h; n=3 matched biological repeats for UNTR/PD treated 48 h). Unpaired t-test shown. **(D)** Workflow of *in vivo* transplantation of LT-HSCs cultured in EXPER_CB system (24h and 72h) and CD34^+^/CD38^-^ cells cultured in the GT_mPB system (62h). UNTR/PD treated cells transplanted in matched cell dose experiments. Created with BioRender (license agreement: KE26QKHW50). **(E)** Graft size (% of human CD45^++^ and GlyA^+^) at 18-week post transplantation of UNTR/PD treated CB LT-HSCs cultured for 72h in EXPER system (n=5 biological experiments; graph representative of engrafted mice only, n=42 PD mice, n = 38 UNTR mice). Two-way ANOVA with Sidak’s multiple comparisons performed (50 cells UNTR vs 50 cells PD p=0.9552; 300 cells UNTR vs 300 cells PD p=0.4084; 700 cells UNTR vs 700 cells PD p=0.971). **(F)** Graft size (% of human CD45^++^ and GlyA^+^) at 18-week post transplantation of mPB CD34^+^CD38^-^ cells after GT protocol culture for 62h including LV (n=3 biological repeats; graph representative of engrafted mice only, n=25 mice UNTR, n=26 mice PD). Two-way ANOVA with Sidak’s multiple comparisons performed (all cell doses UNTR vs PD p>0.9). **(G)** % LTRC in CB LT-HSCs cultured in EXPER system in presence or absence of PD, determined at 24h (n=31 mice UNTR, n=30 mice PD) and 72h (n= 42 mice PD, n=40 mice UNTR). Numerical estimates for LTRC frequency available in Table S2. ELDA statistics (24h UNTR vs 24h PD *p=0.405*; 72h UNTR vs PD *p=0.426*). **(H)** LDA of secondary transplantation experiment from EXPER_CB UNTR/PD 72 h primary mice cohort. Secondary animals were transplanted with sorted CB CD45^++^ from primary recipients (n= 20 mice total; 10 UNTR, 10 PD; n=1 experiment; Table S10). ELDA statistical test performed (*p=0.190*). **(I)** LDA of secondary transplantation experiment from GT_mPB UNTR/PD 62 h primary mice cohort. Secondary animals were transplanted with whole mouse BM isolated from primary recipients (n=21 mice total; UNTR = 11 mice; PD = 10 mice; n=1 experiment; Table S10). ELDA statistical test performed (*p= 0.860*).

It remains unclear if progression past early G_1_ contributes to initiation of differentiation in HSCs as it does in other stem cells^47–49^. Transcriptional priming of differentiation occurs from 24h of culture onwards as HSC begin cycling (Fig.2K). Interestingly, compared to UNTR conditions, several myelo-erythroid progenitor signatures were significantly downregulated in PD treated LT-HSCs (Fig.4A), with genes linked to neutropoiesis (*PRTN3, ELANE* and *CTSG*^50^,Fig.S5A) and erythropoiesis (*TPI1*^51^; Table S8) significantly reduced. Given that temporary cell cycle blockade with PD did not impact *in vitro* terminal differentiation outcomes (Fig.S4B-E), we interpret these data as a delay in lineage specification rather than a change in lineage instruction. We conclude that S-G_2_-M progression facilitates the transcriptional onset of differentiation in *ex vivo* HSCs.

### Loss of long-term repopulation capacity *ex vivo* is independent of cell cycle progression

Next, we tested whether inhibition of cell cycle progression impacts HSC functional hallmarks. An increase in cell size (Fig.4B) and mitochondrial activity (Fig.4C), both HSC activation hallmarks ^15,25,46^ were observed during EXPER_CB LT-HSC culture and were unchanged upon PD induced G_1_ arrest, demonstrating that cell growth and mitochondrial metabolism are independent of cell cycle progression.

To formally determine if progression past early G_1_ contributes to loss of long-term repopulation capacity, we calculated the %LTRC upon *ex vivo* culture with PD, with a similar LDA set up as in Fig.1 (Fig.4D, Table S1, S9, Fig.S5B). LT-HSCs treated with PD exhibited loss of function indistinguishable from UNTR controls. At 18 weeks post-transplantation, we observed no difference in graft size nor lineage output in mice transplanted with EXPER_CB cells cultured with PD for 24h (Fig. S5C-D), 72h (Fig.4E, Fig.S5E) or 62h in the GT_mPB system (Fig.4F, Fig.S4F). LDA found no difference in the %LTRC observed between PD treated and UNTR cells cultured for any duration in either EXPER_CB conditions (24h *p=0.405;* 72h *p=0.426*, Fig.4G, Table S1, S9). Secondary transplantation experiments revealed that the frequency of serially-transplantable HSCs in the injected population was comparable between PD and UNTR cells for both EXPER_CB (Fig.4H; *p=0.190*) and GT_mPB (Fig.4I; *p=0.860*) conditions (Table S10) with no significant difference in graft size observed (Fig.S5G-H). Since PD does not compromise LT-HSC function (Fig.S5B-E), our transplantation experiments demonstrate that cell cycle progression past early G_1_ is not the main driver of loss of human LT-HSC function in culture.

### Inhibition of JAK/STAT signaling during adaptation improves LT-HSC self-renewal

**Figure 5:**
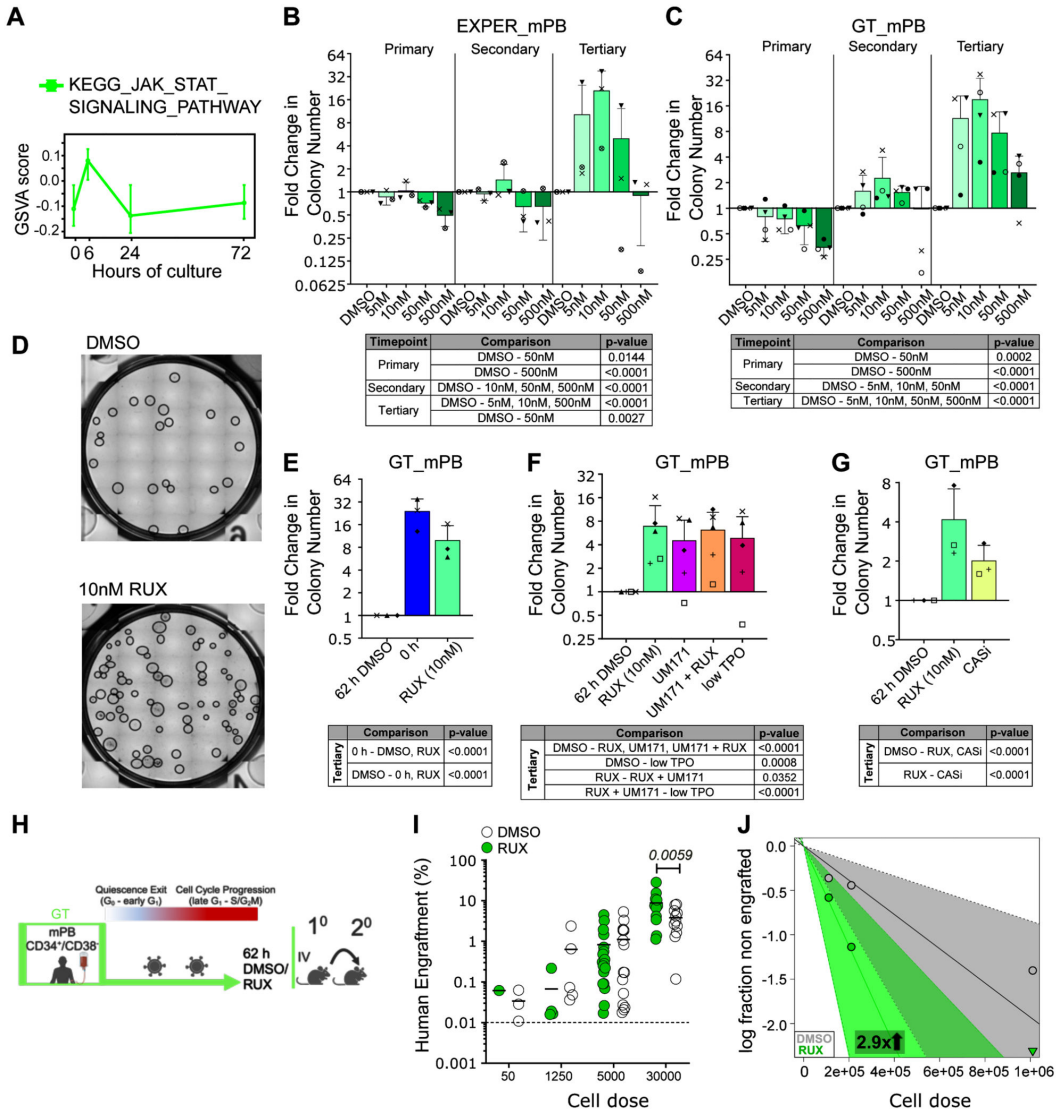
RUX treatment improves serial replating ability and self-renewal capacity of cultured HSCs. **(A)** GSVA score of KEGG JAK/STAT signaling pathway gene-set (same as Fig.2H). GSVA score calculated per single cell with line at median and upper and lower whiskers indicate 25th and 75th percentile of expression. **(B-C)** Serial replating of human mPB HSC/MPPs (CD34^+^CD38^-^CD45RA^-^) cultured for 72h in the EXPER system **(B)** or for 62h in GT **(C)**. Graphs (upper panels) show fold change in colony number compared to DMSO from primary (2 week), secondary (4 week) and tertiary (6 week) plating. n=3 independent mPB samples in (**B**) and n=4 independent mPB samples in **(C)**, individual donors indicated by shapes. Mean and SD shown. Tables (lower panels) report statistics from generalised-linear-mixed-effect-model (glmer) analysis performed with raw colony counts. Tukey corrected p-values for pairwise comparisons to DMSO where *p<0.05* are shown (Table S12 for all comparisons and raw data). **(D)** Representative image of wells from tertiary replating experiment of mPB HSC/MPPs cultured in 72h EXPER conditions (upper panel) or 62h GT conditions (lower panel) treated with either DMSO (left) or 10nM RUX (right). Circles indicate manually scored colonies. Images brightened by 17%. **(E-G)** Serial replating of human mPB HSC/MPPs cultured for 62h in GT. Graphs (upper panels) show fold change in colony number compared to DMSO from tertiary (6 week) plating in the conditions indicated: **(E-G)** RUX (10 nM), **(E)** 0h: fresh HSC/MPPs, **(F)** UM171 (35nM), low TPO (20ng/ml), **(G)** CASi: pan-caspase inhibitor Z-VAD(OH)-FMK (100nM); **(E)** and **(G)** n=3 independent mPB samples, **(F)** n=5 independent mPB samples. Individual donors indicated by shapes and matching across **(E-G)**. Mean and SD shown. Tables (lower panels) report statistics from glmer analysis fitting raw colony counts. Tukey corrected p-values for pairwise comparisons of EM means where *p<0.05* are shown (Table S12 for all comparisons and raw data). **(H)** Workflow of *in vivo* transplantation of mPB CD34^+^CD38^-^ cells cultured in the GT system for 62h with LV transduction with RUX (10nM) or DMSO. Secondary transplantations were performed from whole BM of engrafted mice. Created with BioRender (license agreement: UR26QKHL5H). **(I)** Graft size (% of human CD45^++^ and GlyA^+^) at 18-weeks post transplantation in the BM of mice transplanted with mPB CD34^+^CD38^-^ cells cultured for 62h in GT protocol with RUX (10nM) or DMSO. n=6 biological repeats; graph representative of n=68 engrafted mice (n=34 DMSO, n=34 RUX). Two-way ANOVA with Sidak’s multiple comparisons performed (30,000 cells DMSO vs 30,000 cells RUX *p=0.0059*, all other doses DMSO vs RUX p>0.9). **(J)** Log-fraction plot of limiting dilution model fitted to data in Table S14. Indicates %LTRC estimates from secondary transplantation experiments. Whole BM of engrafted mice from primary transplants was transplanted in NSG-SGM3 mice and analyzed 8 weeks post-transplantation (n=1 experiment; n=35 mice). Slope is log-active fraction. Dotted line is 95% CI. Zero negative response indicated by triangle.

We next hypothesized that inhibition of signaling pathways induced at adaptation may reduce *ex vivo* HSC functional attrition. JAK/STAT signaling stood out as a potential candidate, as JAK/STAT target genes are transiently upregulated 6h post culture initiation (Fig.5A). Consistently, upon 30 minutes of GT culture, mPB HSC/Multipotent Progenitors (HSC/MPP, CD34^+^CD38^-^CD45RA^-^) significantly increased phosphorylation of STAT5, STAT3 and to a lesser extent STAT1 (Fig.S6A-B, Table S11). STAT phosphorylation could be decreased by addition of Ruxolitinib (RUX), an FDA-approved JAK inhibitor, to the culture (Fig.S6A-B). To determine the effect of RUX treatment in HSC culture systems, we assessed the differentiative and proliferative output of RUX treated mPB LT-HSCs cultured in MEM medium for 14 days. Absolute cell numbers per well significantly decreased with increasing doses of RUX (Fig.S6C), but the proportions of erythroid progenitors or myeloid cells did not change significantly, particularly at concentrations ≤50nM (Fig.S6D). RUX treatment therefore globally decreases differentiation with no major impact on lineage commitment, suggesting it may maintain LT-HSCs *ex vivo*.

To test whether RUX addition *ex vivo* could improve the regenerative function of cultured HSCs, we performed serial replating of mPB HSC/MPPs in both GT and EXPER conditions as an *in vitro* surrogate for self-renewal. By tertiary replating, where colonies are largely generated by LT-HSCs, substantial increases in the number of colonies were observed at low doses of RUX in both media and across all donors (GT: 3-38-fold increase; EXPER: 3-37-fold; Fig.5B-D, Table S12). At 500nM RUX, the positive effect was dampened or abrogated, likely due to decreased proliferation (Fig.S6C) and potential off-target inhibition of TRK-B activity^52,53^. The number of colonies produced by 10nM RUX treated HSC/MPPs was still significantly lower than those generated by non-cultured (0h) HSC/MPPs (Fig.5E, Table S12), indicating that RUX reduces the loss of functional HSC induced during *ex vivo* adaptation, rather than by expanding the absolute number of initial HSCs.

Given the clinical need to develop robust human HSC expansion methods *ex vivo*, we performed serial replating to benchmark RUX action against previously reported strategies. In this assay, RUX (10nM) treatment generated significantly higher numbers of tertiary colonies than treatment with UM171(35nM), a pyrimidoindole derivate in clinical trials for HSC expansion applications^54–56^ (RUX: 5-16-fold compared to DMSO; UM171: 0.7-8-fold compared to DMSO; Fig.5F, Table S12). Interestingly, RUX and UM171 did not synergise in this assay (Fig.5F). RUX (10nM) treatment also outperformed lowering TPO concentration, a strategy beneficial in human HSC expansion cultures^57^ (0.3-8-fold compared to DMSO; Fig.5F). Finally, a pan Caspase inhibitor (CASi; Z-VAD(OH)-FMK, 100nM), previously used for CD34^+^ expansion^58^, only led to a modest increase in the tertiary replating ability of mPB HSC/MPPs (1.5-3-fold compared to DMSO; Fig.5G, Table S12).

To formally assess whether *ex vivo* RUX treatment can increase the *in vivo* long-term repopulation ability of cultured LT-HSCs, we treated mPB CD34^+^CD38^-^ with 10nM RUX for 62h and performed serial transplantations with an LDA design as in Fig.4 (Fig.5H). No difference in graft size was observed at 8 weeks in PB (Fig. S6E). However, at 18 weeks post-transplant, the grafts of mice transplanted with high cell doses of RUX treated CD34^+^CD38^-^ were significantly larger (Fig.5I, *p=0.0059*, Fig.S6F) than those of control mice. Whereas the %LTRC determined by LDA statistics were similar after primary transplantation (Table S13), we observed a ~3-fold increase in the %LTRC in RUX treated CD34^+^CD38^-^ compared to DMSO after secondary transplantation (Fig.5J, *p=0.0491*, Fig.S6G, Table S14). These data collectively demonstrate that *ex vivo* JAK/STAT signaling inhibition via RUX improves HSC regenerative function.

## Discussion

Our study establishes the sequence of transcriptional and functional events occurring to human LT-HSCs during their first *ex vivo* division. Our data demonstrate that the early phases of LT-HSC adaptation to culture are critically detrimental to their function, irrespective of cell cycle progression. Our data also indicate the potential of targeting key pathways induced at adaptation, such as JAK/STAT signalling, to improve the safety and efficacy of any clinical protocol involving HSC culture.

It has long been assumed that division causes loss of LT-HSC function *ex vivo. In vivo*, there is a causative relationship between excessive proliferation of LT-HSCs and loss of self-renewal (reviewed in ^59^). In addition, self-renewal capacity is enriched *in vivo* in G_0_ HSCs compared to those in G_1_ or S-G_2_-M^59,60^. *Ex vivo*, the G_0_/G_1_ fraction of cultured CD34^+^ is enriched in phenotypic HSC and contains the highest frequency of repopulating cells^30,61^. Using a unique experimental system which efficiently and reversibly arrests LT-HSCs progression past early G_1_ *ex vivo*, we demonstrate that cell cycle progression and division do not drive the sizeable loss of HSC long-term repopulation capacity observed *ex vivo* over 62/72h. These findings allay concerns that gene editing strategies requiring progression through S phase may be associated with poor engraftment^62^.

Differentiation is tightly connected to cell cycle in most stem cell types, including HSCs^47–49,59,63–65^, where CDKs and cyclins control HSC fate regulators through direct phosphorylation or chromatin binding^66–69^. We observe that temporary cell cycle blockade dampens or delays upregulation of myelo-erythroid lineage specification gene signatures, indicating that cell cycle progression facilitates the establishment of LT-HSC differentiation programs. HSC self-renewal and lineage commitment are controlled independently *in vivo* and *in vitro* in mice^70–72^. Our study of cultured human HSCs further supports this concept by temporally uncoupling loss of repopulation capacity (occurring pre-Rb phosphorylation) from establishment of differentiation (occurring post-Rb phosphorylation).

Given the extensive transcriptional rewiring occurring within 24h of culture, HSC loss of function in this time window is certainly multifactorial. Adaptation, via its characteristic transient expression dynamics and high intercellular variability of stress related genes, likely purges functional HSCs through differentiation or death, akin to the preferential culling of unfit HSCs occurring *in vivo* in response to oxidative, genotoxic and proteostatic damage^10–13,26,73,74^. Early metabolic adaptations also likely contribute to later LT-HSC functional loss. Here we observe transient upregulation of the ceramide biosynthesis enzyme, DEGS1, the inhibition of which preserves LT-HSC repopulation capacity after *ex vivo* culture^26^. Finally, we demonstrate that early induction of JAK/STAT signalling is detrimental to HSC function during culture.

The availability of large numbers of functionally fit HSCs remains a major limiting factor for most HSC transplantation and GT. Our data identify the FDA approved JAK1/2 inhibitor, as an effective compound to mitigate loss of HSC function during *ex vivo* culture. Independent work from our groups demonstrated that tyrosine-unphosphorylated STAT5 (uSTAT5) is a critical regulator of self-renewal vs differentiation decisions in mouse HSCs^75^ (Williams et al., unpublished). RUX shifts the uSTAT5/pSTAT5 ratio towards increased uSTAT5, promoting mouse HSC self-renewal (Williams et al., unpublished). We propose this same mechanism underlies the beneficial effect of RUX on cultured human HSCs. This could be complemented by direct or indirect action of RUX on the MAPK^76^, PI3K^57,77^, NF-kB^78^ and TGF-b^79^ pathways. More broadly, by pinpointing irreversible HSC fate decisions to the first 24h of culture, our work identifies an early functional bottleneck and an untapped window of opportunity to preserve HSC function in any application that requires an *ex vivo* step. Although shortening HSC GT protocols may be useful^80^, our data indicate that it should be complemented with strategies to minimize early HSC functional attrition. Similarly, targeting early HSC adaptation could further improve HSC expansion, complementing current protocols optimized to expand HSC over prolonged culture periods^55–57,81^.

## Supplementary Material

Supplementary Material

## Data Availability

Sequencing files and metadata associated to Smart-Seq2 datasets are deposited at GEO with accession numbers GSE213365 (time-course of EXPER_CB LT-HSCs) and GSE213370 (time-course of GT_mPB LT-HSCs) under the super series GSE213372. All code is publicly available on GitHub: https://github.com/elisa-laurenti/LT-HSC_exvivo. The datasets presented here are also browsable at the following urls: https://bioinf.stemcells.cam.ac.uk/shiny/laurenti/LT_HSC_exvivo_CB/ https://bioinf.stemcells.cam.ac.uk/shiny/laurenti/LT_HSC_exvivo_CB_PD/ https://bioinf.stemcells.cam.ac.uk/shiny/laurenti/LT_HSC_exvivo_mPB_PD/ All data needed to evaluate the presented conclusions are in the main text/Supplemental Materials. All other data/materials will be distributed by authors upon request.

## Key points

- Substantial attrition of hematopoietic stem cell function occurs within 24 hours of *ex vivo* culture independently of cell cycle progression.
- Inhibition of JAK/STAT signaling during culture adaptation via Ruxolitinib improves HSC function *ex vivo*.
