# Adaptation to ex vivo culture reduces human hematopoietic stem cell activity independently of the cell cycle^∗^

**DOI:** 10.1182/blood.2023021426

**Published:** 2024-05-31

**Authors:** Carys S. Johnson, Matthew Williams, Kendig Sham, Serena Belluschi, Wenjuan Ma, Xiaonan Wang, Winnie W. Y. Lau, Kerstin B. Kaufmann, Gabriela Krivdova, Emily F. Calderbank, Nicole Mende, Jessica McLeod, Giovanna Mantica, Juan Li, Charlotte Grey-Wilson, Michael Drakopoulos, Shaaezmeen Basheer, Shubhankar Sinha, Evangelia Diamanti, Christina Basford, Nicola K. Wilson, Steven J. Howe, John E. Dick, Berthold Göttgens, Anthony R. Green, Natalie Francis, Elisa Laurenti

**Affiliations:** 1 Wellcome and Medical Research Council Cambridge Stem Cell Institute, University of Cambridge, Cambridge, United Kingdom; 2 Department of Haematology, University of Cambridge, Cambridge, United Kingdom; 3 Cell Process Development, Cell and Gene Therapy, GlaxoSmithKline, Stevenage, United Kingdom; 4 Princess Margaret Cancer Center, University Health Network, Toronto, Canada; 5 Department of Gene Therapy and Regenerative Medicine, King’s College London, London, United Kingdom

## Abstract

Loss of long-term hematopoietic stem cell (LT-HSC) function ex vivo hampers the success of clinical protocols that rely on culture. However, the kinetics and mechanisms through which this occurs remain incompletely characterized. In this study, through time-resolved single-cell RNA sequencing, matched in vivo functional analysis, and the use of a reversible in vitro system of early G_1_ arrest, we defined the sequence of transcriptional and functional events that occur during the first ex vivo division of human LT-HSCs. We demonstrated that the sharpest loss in LT-HSC repopulation capacity happens early on, between 6 and 24 hours of culture, before LT-HSCs commit to cell cycle progression. During this time window, LT-HSCs adapt to the culture environment, limit the global variability in gene expression, and transiently upregulate gene networks involved in signaling and stress responses. From 24 hours, LT-HSC progression past early G_1_ contributes to the establishment of differentiation programs in culture. However, contrary to the current assumptions, we demonstrated that the loss of HSC function ex vivo is independent of cell cycle progression. Finally, we showed that targeting LT-HSC adaptation to culture by inhibiting the early activation of JAK/STAT signaling improves HSC long-term repopulating function ex vivo. Collectively, our study demonstrated that controlling early LT-HSC adaptation to ex vivo culture, for example, via JAK inhibition, is critically important to improve HSC gene therapy and expansion protocols.

## Key Points

• Substantial attrition of HSC function occurs within 24 hours of ex vivo culture independently of cell cycle progression.
• Inhibition of JAK/STAT signaling during culture adaptation via ruxolitinib improves HSC function ex vivo.

## Visual Abstract

**Figure:**
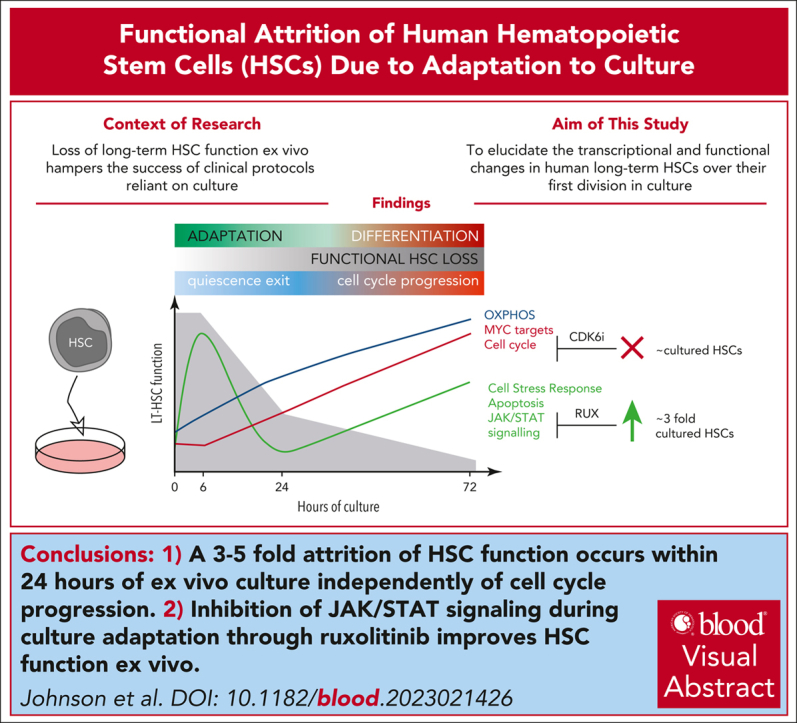

## Teaser

Culture of long-term hematopoietic stem cells (LT-HSCs) is associated with a loss of stem cell potential, a change that is thought to occur as the result of in vitro cell division. Johnson et al used single-cell RNA sequencing to characterize the gene expression changes that occur in this process and showed that, contrary to the accepted dogma, the loss of LT-HSC function is independent of cell-cycle progression. The authors further demonstrated that inhibition of early JAK/STAT signaling improved LT-HSC repopulation function.

## Introduction

A trillion blood cells are produced daily in humans. This impressive output is achieved by rare hematopoietic stem cells (HSCs) that have the unique capacity of driving production of all blood cell types (differentiation), while maintaining a functional HSC pool (self-renewal). Owing to this extensive regenerative capacity, HSCs are the key functional units of HSC transplantation and HSC gene therapy (GT). HSC GT promises to be a curative, one-time treatment, and is under clinical investigation to treat more than 10 monogenic diseases.^1^ HSC GT currently requires an ex vivo culture step, which strongly reduces HSC functionality.^2^^,^^3^ Minimizing HSC attrition during ex vivo protocols would provide patients with larger numbers of functional HSCs, improve treatment efficacy and safety in current HSC GT indications, and pave the way for new therapeutic applications.

Long-term blood formation post-transplantation is driven by predominantly quiescent long-term HSCs (LT-HSCs).^4^^,^^5^ Quiescence (G_0_) is defined as the reversible absence of cell cycling. G_0_ is characterized by decreased cell size, reduced protein biosynthesis,^6^^,^^7^ high autophagic recycling,^8^^,^^9^ an elevated basal expression of cell stress response pathways,^10^, ^11^, ^12^, ^13^ a relatively inactive glycolytic metabolism,^14^^,^^15^ and is maintained in vivo by the hypoxic bone marrow niche.^16^^,^^17^ Upon stimulation, HSCs exit quiescence (G_0_ to early G_1_) and progressively phosphorylate retinoblastoma (Rb) until the restriction point where commitment to division occurs and cell cycle progression (late G_1_ to S to G_2_ to M) ensues. CDK6 is a master regulator of HSC quiescence exit kinetics whose differential regulation within the HSC pool guarantees LT-HSC maintenance.^18^ An emerging body of work has demonstrated further heterogeneity within the quiescent state of LT-HSCs^4^ that is associated with distinct lineage preferences and kinetics of reconstitution after transplantation.^19^, ^20^, ^21^, ^22^ Clinical GT protocols target the CD34^+^ fraction (a heterogeneous mixture of HSCs and progenitor cells) with a limited understanding of how ex vivo protocols impact the underlying biology unique to the LT-HSC population.

In current culture systems, HSCs inevitably exit quiescence and divide. Ex vivo culture increases protein synthesis rates,^6^^,^^23^ remodels the mitochondria to an activated state^24^ with increased oxidative metabolism and reactive oxygen species production,^25^ disrupts optimal proteostasis programs,^23^^,^^26^ and reduces the dependency on lysosomal recycling.^27^ These changes are all associated with a net decline in long-term repopulation capacity. Early studies showed that it is exclusively HSCs and progenitor cells in G_0_ before^28^ and after culture^29^^,^^30^ that engraft and not their cycling counterparts. From this and from an extensive body of literature on in vivo models,^31^, ^32^, ^33^ it has been assumed that cell cycle progression itself drives the loss of HSC function in culture. However, to date, no study has formally addressed this question in human LT-HSCs that undergo clinically relevant culture. Despite recent advances in strategies to expand HSCs ex vivo^34^ and the increased clinical adoption of HSC GT, we are still lacking a kinetic and mechanistic understanding of how HSCs lose self-renewal capabilities ex vivo. In this study, by pairing single-cell RNA sequencing (scRNA-seq) with in vivo functional analysis in a time-resolved manner, we dissect the transcriptional and functional changes that occur in highly purified human LT-HSCs during their first division in culture.

## Methods

### Human samples

Human biological samples were sourced ethically, and the research was conducted in accordance with the terms of the informed consents under an institutional review board/research ethics committee–approved protocol as specified below. Primary cord blood (CB) samples were obtained with informed consent from healthy donors from the Cambridge Blood and Stem Cell Biobank. CBs of both sexes were processed as a single sample, except for scRNA-seq for which only single sex CB pools were used. Mobilized peripheral blood (mPB) was obtained from healthy male donors, aged 25 to 28 years, by administering daily filgrastim (Neupogen; 10 μg/kg per day) for 5 days. Apheresis was performed on day 5 and 6 using the Optia Spectra (Terumo Blood and Cell Technologies). Sample preparation, flow cell sorting, and phenotyping are reported in the supplemental Methods, available on the *Blood* website.

### Ex vivo culture

Two complementary culture systems were used, namely (1) experimental (EXPER) conditions comprising StemPro base media (Thermo Fisher Scientific) supplemented with nutrients (0.028%; Thermo Fisher Scientific), penicillin and streptomycin (1%), l-glutamine (1%), human low-density lipoprotein (50 ng/mL) (Stem Cell Technologies), and the following cytokines: stem cell factor (100 ng/mL), fms-like tyrosine kinase-3 ligand (FLT3L; 20 ng/mL), thrombopoietin (TPO; 100 ng/mL), erythropoietin (EPO; 3 units per mL), interleukin-6 (IL-6; 50 ng/mL), interleukin-3 (IL-3; 10 ng/mL), and granulocyte-macrophage colony-stimulating factor (GM-CSF; 20 ng/mL); and (2) gene therapy (GT) conditions comprising GMP Stem Cell Growth Medium (CellGenix) supplemented with l-glutamine (1%) (Thermo Fisher Scientific), penicillin and streptomycin (1%) (Thermo Fisher Scientific), and the following cytokines: SCF (300 ng/mL), FLT3L (300 ng/mL), IL-3 (60 ng/mL), and TPO (100 ng/mL). All cytokines were from Miltenyi/Peprotech, except EPO (Janssen). The CDK4/CDK6 inhibitor palbociclib (PD; PD0332991; Sigma, 200 nM), the JAK1/JAK2 inhibitor ruxolitinib (RUX; Selleckchem; 5-500 nM), UM171 (Stem Cell Technologies, 35 nM), or Z-VAD(OH)-FMK (Cayman Chemical, 100 nM) were added to GT, EXPER, or media supporting myeloid-erythroid-megakaryocytic (MEM) colony generation when indicated. The methodology for lentiviral vector production and all in vitro assays to assess HSC function, cell cycle, differentiation, and serial replating ability are described in the supplemental Methods.

### Xenograft transplantation

NOD.Cg-PrkdcscidIl2rgtm1Wjl/SzJ (NSG) mice or NOD.Cg-Prkdcscid Il2rgtm1Wjl Tg(CMV-IL3,CSF2,KITLG)1Eav/ MloySzJ (NSG-SGM3) mice were bred in-house or obtained from Charles River. All animals were housed in a specific pathogen-free animal facility, and experiments were conducted according to the UK Home Office regulations or in accordance with the institutional guidelines approved by the University Health Network Animal Care Committee. The primary and secondary transplantation methods and analysis criteria are reported in the supplemental Methods.

### scRNA-seq

scRNA-seq libraries were prepared using the Smart-Seq2 protocol,^35^ adapted as described in the supplemental Methods. Libraries were quantified using the KAPA library quantification kit (Roche) and were sequenced by paired-end sequencing (150 bp) using the Illumina HiSeq4000 (Illumina) and NovaSeq 6000 (Illumina) at the Cancer Research UK Cambridge Institute genomics core facility (Cambridge, United Kingdom). The scRNA-seq experimental design and bioinformatic methods are described in the supplemental Methods.

### Statistical analysis

Appropriate statistical tests were performed using GraphPad Prism (v9.3), R Studio (v1.2), and Python (v3.8.6), further described in the supplemental Methods.

CB collection and experimental work on CB and mPB CD34^+^ cells were performed in accordance with regulated procedures approved under the 07/MRE05/44 and 18/EE/0199 research ethics committee research studies. Animal studies were ethically reviewed by the University of Cambridge Animal Welfare and Ethical Review Body and carried out in accordance with the Animals (Scientific Procedures) Act 1986, the GlaxoSmithKline policy on the care, welfare and treatment of animals, and institutional guidelines approved by the University Health Network Animal Care Committee.

## Results

### Loss of HSC function occurs early during ex vivo culture

HSC functional attrition in culture severely reduces the efficacy of HSC clinical approaches like ex vivo GT. We therefore sought to study the kinetics of this phenomenon using (1) purified human LT-HSCs (CD34^+^CD19^−^CD38^−^CD45RA^−^CD90^+^CD49f^+^),^4^ (2) early culture time points (≤72 hours) corresponding to key HSC cell cycle transitions,^18^ (3) 2 distinct ex vivo systems and HSC sources, namely “GT_mPB conditions” using mPB LT-HSCs and 2 hits of transduction with a lentiviral vector expressing GFP^2^^,^^36^^,^^37^; and “EXPER_CB conditions” using CB LT-HSCs (unless otherwise indicated) and promoting differentiation.^18^

**Figure 1.**
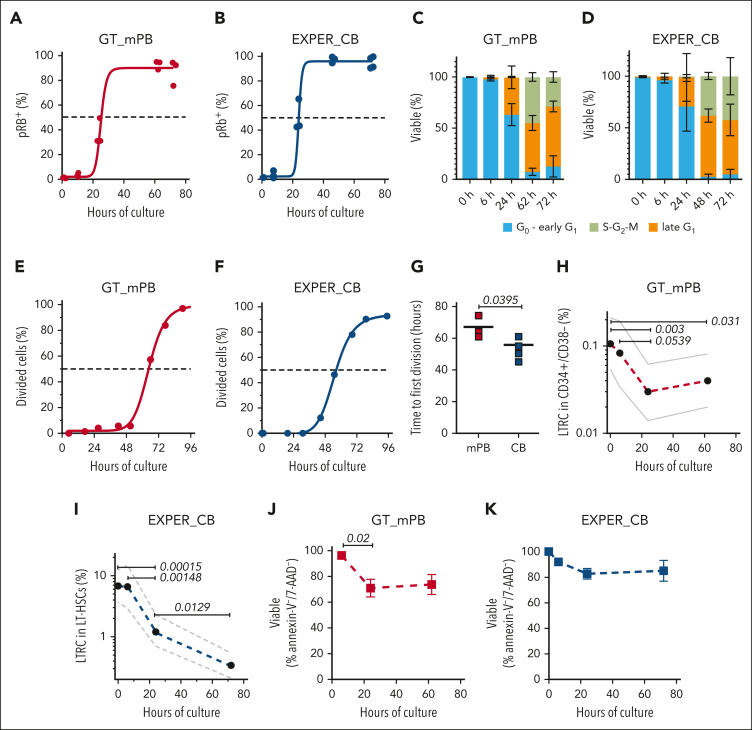
**Kinetics of cell cycle progression, survival, and loss of long-term repopulation capacity of LT-HSCs during ex vivo culture.** (A) The cumulative quiescence exit kinetics of GT_mPB LT-HSCs are shown, determined by phosphorylation of Rb (pRb; at Ser 807-811) using flow cytometry analysis. The curve is a least-squares sigmoidal fit with half maximal effective concentration (EC_50_) = 24.7 hours; there are n = 3 biological replicates for 0 hours, 24 hours, 62 hours, and 72 hours and n = 4 biological replicates for 6 hours. Standard error ± 5.186; R^2^ = 0.9797. (B) The cumulative quiescence exit kinetics of EXPER_CB LT-HSCs are shown, determined by pRb (at Ser 807-811) using flow cytometry analysis. The curve is a least-squares sigmoidal fit with an EC_50_ = 24.72 hours; there are n = 3 biological replicates for 0 hours, 6 hours, 24 hours, and 48 hours and n = 4 biological replicates for 72 hours. Standard error ± 3.944; R^2^ = 0.9844. (A-B) The dashed line indicates the EC_50_ at the time of quiescence exit. (C) Cell cycle phase assignment of GT_mPB LT-HSCs determined by pRb/DAPI flow cytometry analysis. Equivalent repeats as in panel A. (D) The cell cycle phase assignment of EXPER_CB LT-HSCs determined by pRb/DAPI flow cytometry analysis. Equivalent repeats as in panel B. (E) The cumulative first-division kinetics (excluding dead cells) of GT_mPB LT-HSCs are shown. The curve is a least-squares sigmoidal fit. Representative examples are shown (n = 3 biological replicates). The dashed line indicates the EC_50_. The EC_50_ = 64.83 hours; 95% confidence interval (CI) = 62.83-66.87; R^2^ = 0.9976. (F) The cumulative first-division kinetics (excluding dead cells) of EXPER_CB LT-HSCs are shown. The curve is a least-squares sigmoidal fit. Representative examples shown (n = 4 biological replicates). EC_50_ = 55.44 hours; 95% CI = 54.28-56.70; R^2^ = 0.9995. (G) The time to first-division kinetics summary of LT-HSCs cultured in (E) GT_mPB (F) and EXPER_CB systems (n = 3 biological replicates for GT_mPB; n = 4 biological replicates for EXPER_CB). Unpaired *t* tests are shown. (H) The percentage of LTRC in GT_mPB CD34^+^CD38^−^ cells at each time point, as determined by LDA analysis in the transplanted population ± 95% CIs is shown. The LTRC frequency estimates for GT_mPB are as follows: 0 hours, 1 in 939 (29 mice); 6 hours, 1 in 1211 (21 mice); 24 hours, 1 in 3371 (23 mice); 62 hours, 1 in 2510 (23 mice). Extreme Limiting Dilution Analysis (ELDA) statistical tests are shown. The data are shown in supplemental Table 1. (I) The percentage of LTRC in LT-HSCs cultured in EXPER_CB systems at each time point, based on LDA analysis in the transplanted population ± 95% CIs is shown. The LTRC frequency estimates for EXPER_CB are as follows: 0 hours, 1 in 14.8 (31 mice); 6 hours, 1 in 15.2 (19 mice); 24 hours, 1 in 80.6 (31 mice); and 72 hours, 1 in 293.7 (40 mice). ELDA statistical tests are shown. The data are shown in supplemental Table 1. (J) The survival of LT-HSCs cultured in GT_mPB systems, as determined by Annexin-V/7-AAD flow cytometry, is shown for n = 3 biological replicates at each time point. The mean ± SD is shown. (K) Survival of LT-HSCs cultured in EXPER_CB systems, as determined by Annexin-V/7-AAD flow cytometry, is shown for n = 3 biological replicates at each time point. Paired *t* tests are shown for the 6 hours and 24 hours comparison. The mean ± SD is shown. SD, standard deviation.

We first characterized LT-HSC cell cycle kinetics in our ex vivo conditions. LT-HSCs in either the GT or EXPER conditions progressed past late G_1_ at ∼24 hours after culture initiation (Figure 1A-B) as assessed by both phosphorylation of Rb at the serine residue 807 to 811 and acquisition of the Ki-67 marker (supplemental Figure 1A-B). Concurrently, the proportion of cycling cells (S to G_2_ to M phases) increased within the 24- to 72-hour window (Figure 1C-D). LT-HSC time to first division was estimated to be 62.0 ± 6.5 hours for the GT_mPB conditions and 53.4 ± 6.5 hours for the EXPER_CB conditions (Figure 1E-G). Therefore, independent of HSC source and culture conditions, LT-HSCs progress to late G_1_ by ∼24 hours and their first division by 72 hours.

Next, we quantified the long-term repopulating cell frequency (%LTRC) at 0, 6, 24, and 62 or 72 hours of ex vivo culture and performed limiting dilution analysis (LDA) xenotransplantation in NSG mice. We transplanted fixed numbers of (1) GT_mPB CD34^+^ CD38^−^ cells cultured for 62 hours (time of infusion into patients in HSC GT protocols) and (2) EXPER_CB LT-HSCs cultured for up to 72 hours. Human engraftment was analyzed 18 weeks after transplantation. Of note, in GT conditions, almost all engrafted animals displayed GFP^+^ cells (10/11; supplemental Table 1), indicating that transduced cells contributed to long-term engraftment. LDA showed that the %LTRC within the transplanted LT-HSC population was unchanged for the first 6 hours of culture. However, the %LTRC dropped conspicuously by 24 hours (GT, approximately threefold; *P* = .003; EXPER, approximately fivefold; *P* = .00015 when compared with 0 hours). An additional decrease in the %LTRC was observed past 24 hours in the EXPER conditions (approximately twofold; *P* = .0129 when compared to 24 hours) when LT-HSCs progressed through the cell cycle (Figure 1H-I; supplemental Tables 1 and 2). We next measured apoptosis using Annexin V/7-AAD staining. A decrease in viable cells was observed between 6 to 24 hours with no further decrease at the later time point in both the GT_mPB and EXPER_CB culture systems (Figure 1J-K). Interestingly, the loss in LTRC observed between 6 to 24 hours ex vivo largely outnumbered the loss in viability measured in the same time window. We therefore concluded that HSC loss ex vivo occurs predominantly before cells enter the late G_1_ phase of the cell cycle.

### Transcriptome dynamics of LT-HSCs over the first cell cycle ex vivo

**Figure 2.**
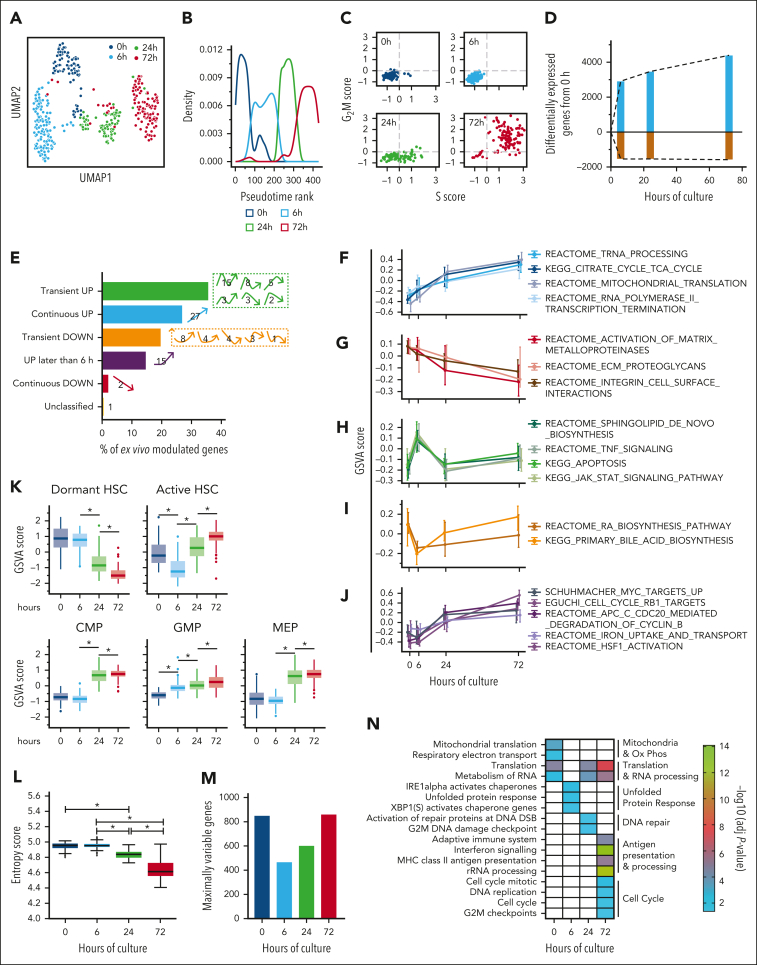
**The dynamics of gene expression over the first division of LT-HSC ex vivo at single-cell resolution.** (A) Uniform manifold approximation and projection (UMAP) of 429 single EXPER_CB LT-HSCs over a time course of 0, 6, 24, and 72 hours (n = 2 independent experiments). The UMAP was generated using the Seurat 4 pipeline after cell cycle regression. (B) A 2-dimensional pseudotime rank plot of EXPER_CB LT-HSCs over the time course generated after cell cycle regression. (C) Transcriptional allocation of cell cycle status at different time points during EXPER_CB LT-HSC culture; n = 429 single cells. (D) The number of differentially expressed genes (false discovery rate < 0.05) at each time point in comparison with the 0 hours time point. Upregulated genes are shown in blue, and downregulated genes are shown in brown. A full list of genes is available in supplemental Table 4. (E) The broad patterns of gene expression that were identified over the time course (8966 genes classified after filtering by the DEG report algorithm). Numbers indicate the percentage of genes that showed the specific patterns of gene expression displayed to the right of the bar. (F-J) GSVA score of c2 curated pathways showing the following specific expression patterns: (F) continuous up; (G) continuous down; (H) transient up; (I) transient down; and (J) up later than 6 hours. The GSVA score that was calculated per single cell with a line at the median and upper and lower whiskers indicating the 25th and 75th percentile of expression. (K) The GSVA scores of indicated published gene signatures, representative of specific HSCs and progenitor cell (HSPC) subsets. The median and interquartile range are shown. ∗*P* < .001. (L) The scEntropy value at each time point is shown (calculated for both batches combined; Wilcoxon rank sum test shown; 0 vs 6 hours; *P* = .835). The median and interquartile range are shown. ∗*P* < .001. (M) Shows the number of maximally variable genes (MVGs) at each time point (supplemental Methods; 2792 genes total). (N) Shows selected biological pathways that are significantly enriched from MVGs (–log_10_[adjusted *P* value] < .05). A full list of the pathways available in supplemental Table 7. CMP, common myeloid progenitor; GMP, granulocyte monocyte progenitor; MEP, myeloid erythroid progenitor.

To better understand the kinetics of LTRC loss ex vivo, we investigated the transcriptional changes associated with the first cell division of LT-HSCs in culture. EXPER_CB LT-HSCs were cultured for 0, 6, 24, and 72 hours before scRNA-seq via an adapted Smart-Seq2 protocol.^35^ A total of 429 cells, collected over 2 independent experiments, passed quality control. Cells from both experiments were integrated through 2 distinct pipelines (Scanpy and Seurat 4) (supplemental Methods), which yielded highly concordant results. LT-HSCs grouped by culture duration in uniform manifold approximation and projection (UMAP) visualizations independently of the batch (Figure 2A; supplemental Figure 2A), of whether cell cycle regression was applied (supplemental Figure 2B), and of the integration pipeline used (supplemental Figure 2C).

At 0 hours, we identified 2 transcriptionally defined subsets of LT-HSCs in accordance with recent reports of transcriptional heterogeneity within the quiescent human LT-HSC fraction (supplemental Note 1; supplemental Figure 2D-H; supplemental Table 3). After 0 hours, pseudotime ordering largely recapitulated chronological time (Figure 2B; supplemental Figure 2I), and transcriptional assignment of cell cycle status agreed with the functional analysis (Figure 2C). LT-HSCs therefore progressed through distinct transcriptional states over time in culture. DESeq2 differential gene expression analysis of all pairwise comparisons in the data set (supplemental Table 4) showed that ex vivo culture has an extensive and rapid effect on the transcriptome. A total of 10 010 genes changed over the time course (hereafter termed “ex vivo modulated genes”; supplemental Table 5). Interestingly, of the gene expression changes that occurred between 0 and 72 hours (5980 genes), 75% (4460 genes) were already observed at 6 hours (Figure 2D) before the vast majority (>90%) of the cells entered late G_1_ in these culture conditions (Figure 1A).

**Figure 3.**
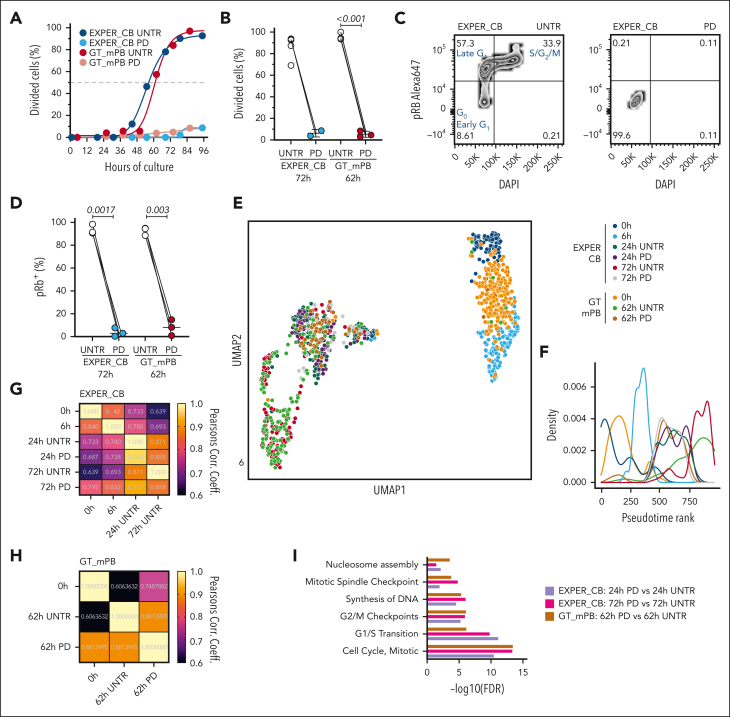
**Transcriptional effects of preventing progression past early G_1_ during ex vivo culture of LT-HSCs.** (A) The cumulative first-division kinetics (excluding dead cells) of UNTR/PD-treated LT-HSCs cultured in a GT_mPB (dark red and light red) or EXPER_CB (dark blue and light blue) system. The curve is a least-squares sigmoidal fit. A representative example is shown (n = 3 UNTR or PD–matched biological replicates: n = 2 UNTR or PD–treated matched biologic replicates). The dashed line indicates the EC_50_ time to first division. UNTR and PD-treated (200 nM) cells are shown. (B) The divided single cells as a proportion of the total alive cells at 96 hours are shown (EXPER_CB: n = 5 biological replicates, n = 2 UNTR or PD–treated matched biological repeats; GT_mPB: n = 3 matched biological replicates). Paired *t* tests are shown. (C) Representative example of the flow cytometry plot for pRb (at Ser 807-811) and DAPI staining on 72 hour EXPER_CB cultured UNTR (left) or PD-treated (right) LT-HSCs. (D) Quantification of pRb^+^ (as a percentage of viable cells) in 62 hour GT_mPB cultured UNTR or PD-treated LT-HSCs (n = 3 UNTR or PD-treated matched biological replicates; no lentiviral vector [LV] transduction) or 72 hour EXPER_CB cultured LT-HSCs (n = 3 UNTR or PD-treated matched biological replicates). Paired *t* tests are shown. (E) UMAP visualization of scRNA-seq of 954 LT-HSCs from the indicated culture conditions (EXPER_CB, 536 single cells; GT_mPB, 418 single cells). Cell cycle regression was applied. (F) The 2-dimensional pseudotime density rank plot of single cells as shown in (E). Cell cycle regression was applied. (G) Pearson correlation coefficient estimates for the comparison of the median expression value of 10903 genes at different time points or conditions for the EXPER_CB data set (union of all differentially expressed genes between any 2 UNTR time points and between PD-treated and UNTR conditions) (available in supplemental Table 5). (H) Pearson correlation coefficient estimates for the comparison of the median expression value of 5469 genes at different time points or conditions for the GT_mPB data set (union of all differentially expressed genes between any 2 UNTR time points and between PD treated and UNTR conditions) (available in supplemental Table 5). (I) Selected Reactome pathways (FDR < 0.05) enriched between PD-treated and UNTR LT-HSCs cultured for matched durations of 24 hours in EXPER_CB (purple), 72 hours in EXPER_CB (pink), and 62 hours in GT_mPB (brown) systems. The full DeSeq2 results and Reactome pathway enrichment are available in supplemental Table 8.

We then analyzed ex vivo modulated genes using degPatterns^38^ to define gene (Figure 2E; supplemental Figure 3A; supplemental Table 6) and pathway (gene set variation analysis (GSVA)^39^; supplemental Figure 3B; supplemental Table 6) expression patterns over the time course. Similar dynamic patterns of upregulation and downregulation were observed at the gene and pathway level (supplemental Figure 3A-B). We found 5 expression patterns of interest, comprising (1) cumulative increases in the expression of pathways linked to transcription, RNA splicing, translation, and oxidative phosphorylation (Figure 2F); (2) progressive decreases in pathways linked to cell adhesion (Figure 2G); (3) early loss of the activator protein-1 transcription factors *JUN* and *FOS*, key to the HSC quiescence network (supplemental Figure 3C); and (4) transient patterns of gene (supplemental Figure 3D) or pathway (Figure 2H-I) expression indicating that HSCs initiate a stress response to adapt to culture signals. Particularly abundant were genes/pathways upregulated from 0 to 6 hours and subsequently decreased at 24 hours (27.3% of the total genes; 76.3% of “transient UP” genes). These included sphingolipid de novo biosynthesis (eg, *DEGS1)*, apoptosis (eg, *CFLAR* and *BIRC2*), and stress pathways (eg, *ATF4*). The fifth pattern was upregulation of expression after the 6 hour time point of genes or gene sets related to proliferation and differentiation (Figure 2J-K). These included *MYC* (supplemental Figure 3E), MYC targets, cell cycle progression, signatures of active human LT-HSCs,^22^ and committed progenitors.^40^ Finally, we calculated scEntropy scores, which decrease during differentiation fate decisions.^41^ scEntropy scores significantly decreased only after 6 hours (Figure 2L), supporting the concept that cell-fate decisions occur at the 6 to 24 hour transition.

Dynamic changes in gene expression variability have been linked to cell-fate decisions in hematopoietic progenitors^42^ and T-cell activation.^43^ We therefore investigated gene expression variability over the time course, employing BASiCS ^44^^,^^45^ and determining maximally variable genes (MVGs) at each time point (supplemental Methods; supplemental Figure 3F). The number of MVGs was lowest at 6 hours (Figure 2M) showing that gene expression variability is restrained early in culture initiation. This is likely attributable to the adaptation of all LT-HSC subsets to the culture environment. MVGs at 6 hours were enriched for unfolded protein response regulatory genes (including *DNAJC3* and *XBP1*; Figure 2N, supplemental Table 7), indicating that despite a global restriction in gene expression variability, LT-HSCs vary in their degree of activation of cell stress response genes at this critical time point.

In summary, our results indicate that, within the first 24 hours, LT-HSCs initially adapt to culture by restricting global gene variability and activating a transient stress response. Subsequently, *MYC* and cell cycle genes are upregulated and differentiation programs are initiated.

### Cell cycle–dependent initiation of differentiation in cultured LT-HSCs

Ex vivo, changes to HSC metabolism and organelle biology occur concomitantly with cell cycle progression and loss of function.^23^, ^24^, ^25^^,^^27^^,^^46^ Our observation that the sharpest drop in LT-HSC repopulation capacity occurs before most LT-HSCs enter late G_1_ prompted us to formally test to what extent progression past early G_1_ contributes to HSC identity and function in culture. We took advantage of the CDK4/CDK6 inhibitor PD (PD033299), previously established to prevent division of CB LT-HSCs.^18^ First, we repeated this finding with LT-HSCs cultured in both the GT_mPB and EXPER_CB systems (Figure 3A-B), showing early G_1_ arrest of CB (Figure 3C-D) and mPB LT-HSCs (supplemental Figure 4A). PD treatment was reversible and did not compromise LT-HSC differentiation (supplemental Figure 4B-E), therefore allowing assessment of the effect of culture in the absence of cell cycle progression.

To disentangle which transcriptional differences in culture depend on cell cycle progression, scRNA-seq was performed on LT-HSCs treated with PD for 24 and 72 hours in the EXPER_CB system and for 62 hours in the GT_mPB system. These data were integrated with 0- and 6-hours cells in a unique embedding using Seurat4 (954 cells; n = 6 independent experiments). As expected, PD-treated LT-HSCs were transcriptionally allocated to G_1_ in both culture systems (supplemental Figure 4F-G). Cultured LT-HSCs pharmacologically arrested in G_1_, localized with the 24-hour untreated (UNTR) LT-HSCs in the UMAP (Figure 3E) and by pseudotime (Figure 3F) independently of the cell source, culture system, duration of PD treatment, and whether cell cycle regression was applied or not (supplemental Figure 4H-I). The impact of PD treatment on the transcriptome was relatively minimal with gene expression correlations being higher when comparing UNTR with PD conditions (Pearson correlation coefficient; EXPER_CB 24 hours, 0.96; EXPER_CB 72 hours, 0.871; GT_mPB 62 hours, 0.881) than when comparing any UNTR time point comparisons (Figure 3G-H). Furthermore, most pathways that were significantly enriched by PD treatment either at 24 hours or at 72 hours were related to cell cycle progression (Figure 3I; supplemental Table 8), demonstrating the low off-target effects of PD.

**Figure 4.**
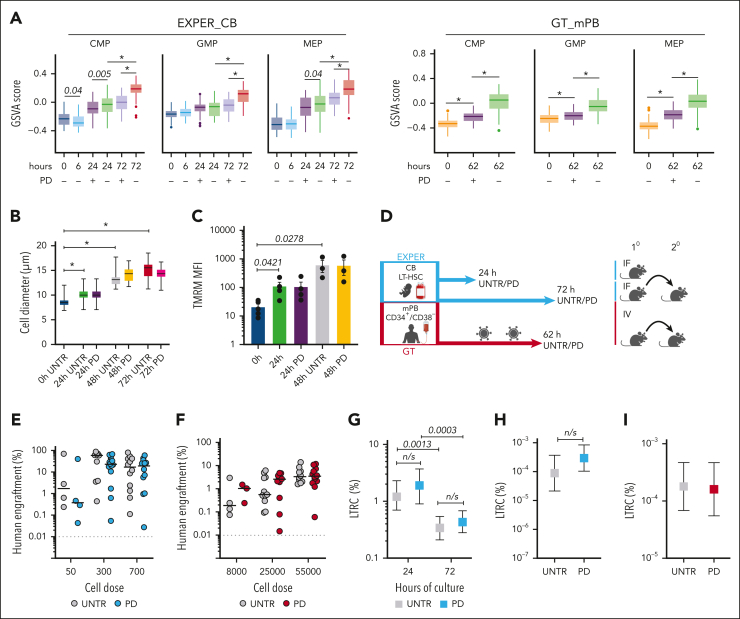
**Preventing progression past early G_1_ during ex vivo culture of LT-HSCs dampens the establishment of differentiation programs but does not affect the loss of long-term repopulation capacity.** (A) GSVA scores for the indicated lineage gene expression signatures from Laurenti et al^40^ at the indicated time points for the LT-HSC culture. The GSVA score is generated per cell, and the line indicates the median. EXPER_CB, n = 536 single cells; GT_mPB, n = 418 single cells. (B) The cell diameters of single EXPER_CB cultured LT-HSCs (n = 2 biological replicates representing n = 469 total single cells). Unpaired *t* tests are shown. ∗*P* < .001. (C) Tetramethylrhodamine (TMRM) staining of bulk EXPER_CB cultured LT-HSCs (n = 5 biological replicates for 0 hours; n = 4 matched biological replicates for UNTR or PD-treated cells for 24 hours; n = 3 matched biological replicates for UNTR or PD-treated cells for 48 hours). Unpaired *t* tests are shown. (D) Workflow of in vivo transplantation of EXPER_CB cultured LT-HSCs (24 hours and 72 hours) and CD34^+^/CD38^−^ cells cultured in the GT_mPB system (62 hours). UNTR or PD-treated cells were transplanted in matched cell dose experiments. (E) Graft size (percentage of human CD45^++^ and GlyA^+^) at 18 weeks after transplantation of UNTR or PD-treated EXPER_CB LT-HSCs cultured for 72 hours (n = 5 biological experiments; the graph is representative of engrafted mice only; n = 42 PD mice; n = 38 UNTR mice). Two-way analysis of variance (ANOVA) with Sidak multiple comparisons performed (50 cells UNTR vs 50 cells PD; *P* = .9552; 300 cells UNTR vs 300 cells PD; *P* = .4084; 700 cells UNTR vs 700 cells PD; *P* = .971). (F) Graft size (percentage of human CD45^++^ and GlyA^+^) at 18 weeks post transplantation of mPB CD34^+^CD38^−^ cells after GT protocol culture for 62 hours including LV transduction (n = 3 biological replicates; the graph is representative of engrafted mice only; n = 25 mice UNTR; n = 26 mice PD-treated). Two-way ANOVA with Sidak multiple comparisons performed (all cell doses UNTR vs PD; *P* > .9). (G) The percentage of LTRC in EXPER_CB cultured LT-HSCs UNTR or PD-treated, determined at 24 hours (n = 31 mice UNTR; n = 30 mice PD) and 72 hours (n = 42 mice PD; n = 40 mice UNTR). Numerical estimates for LTRC frequency available in supplemental Table 2. ELDA statistics (24 hours UNTR vs 24 hours PD; *P* = .405; 72 hours UNTR vs PD; *P* = .426). (H) LDA of secondary transplantation experiment from EXPER_CB cultured LT-HSCs UNTR or PD-treated from the 72 hour primary mice cohort. Secondary animals were transplanted with sorted CB CD45^++^ from primary recipients (n = 20 mice total; 10 UNTR, 10 PD; n = 1 experiment) (supplemental Table 10). An ELDA statistical test was performed (*P* = .190). (I) LDA of secondary transplantation experiment from GT_mPB UNTR or PD-treated from the 62 hour primary mice cohort. Secondary animals were transplanted with whole mouse bone marrow (BM) isolated from primary recipients (n = 21 mice total; UNTR = 11 mice; PD = 10 mice; n = 1 experiment) (supplemental Table 10). An ELDA statistical test was performed (*P =* .860). (D) was created with BioRender.com (license agreement, KE26QKHW50).

It remains unclear if progression past early G_1_ contributes to the initiation of differentiation in HSCs as it does in other stem cells.^47^, ^48^, ^49^ Transcriptional priming of differentiation occurs from 24 hours of culture onward as HSCs begin cycling (Figure 2K). Interestingly, when compared with UNTR conditions, several myelo-erythroid progenitor signatures were significantly downregulated in PD-treated LT-HSCs (Figure 4A) with genes linked to neutropoiesis (*PRTN3*, *ELANE*, and *CTSG*^50^) (supplemental Figure 5H) and erythropoiesis (*TPI1*^51^) (supplemental Table 8) being significantly reduced. Given that temporary cell cycle blockade with PD did not impact in vitro terminal differentiation outcomes (supplemental Figure 4B-E), we interpreted these data to be a delay in lineage specification rather than a change in lineage instruction. We concluded that S to G_2_ to M progression facilitates the transcriptional onset of differentiation in ex vivo HSCs.

### Loss of long-term repopulation capacity ex vivo is independent of cell cycle progression

Next, we tested whether the inhibition of cell cycle progression impacts HSC functional hallmarks. An increase in cell size (Figure 4B) and mitochondrial activity (Figure 4C), both of which are HSC activation hallmarks,^15^^,^^25^^,^^46^ were observed during EXPER_CB LT-HSC culture and were unchanged upon PD-induced G_1_ arrest, demonstrating that cell growth and mitochondrial metabolism are independent of cell cycle progression.

To formally determine if progression past early G_1_ contributed to the loss of long-term repopulation capacity, we calculated the %LTRC in ex vivo culture with PD, using a similar LDA setup as in Figure 1 (Figure 4D; supplemental Tables 1 and 9). LT-HSCs treated with PD exhibited loss of function that was indistinguishable from UNTR controls. At 18 weeks after transplantation, we observed no difference in the graft size, nor in the lineage output, in mice transplanted with EXPER_CB cells cultured with PD for 24 hours (supplemental Figure 5B-C), 72 hours (Figure 4E; supplemental Figure 5D), or GT_mPB cells cultured with PD for 62 hours (Figure 4F; supplemental Figure 5E) with respect to UNTR control cells cultured in the same conditions. LDA found no difference in the %LTRC observed between PD-treated and UNTR cells cultured for any duration in either of the EXPER_CB conditions (24 hours *P* = .405; 72 hours *P* = .426; Figure 4G; supplemental Tables 1 and 9). Secondary transplantation experiments revealed that the frequency of serially transplantable HSCs in the injected population was comparable between PD-treated and UNTR cells for both the EXPER_CB (Figure 4H; *P* = .190) and GT_mPB (Figure 4I; *P* = .860) conditions (supplemental Table 10) with no significant difference in the graft size observed (supplemental Figure 5F-G). Because PD does not compromise LT-HSC function (supplemental Figure 4B-E), our transplantation experiments demonstrated that cell cycle progression past early G_1_ was not the main driver of loss of human LT-HSC function in culture.

### Inhibition of JAK/STAT signaling during adaptation improves LT-HSC self-renewal

**Figure 5.**
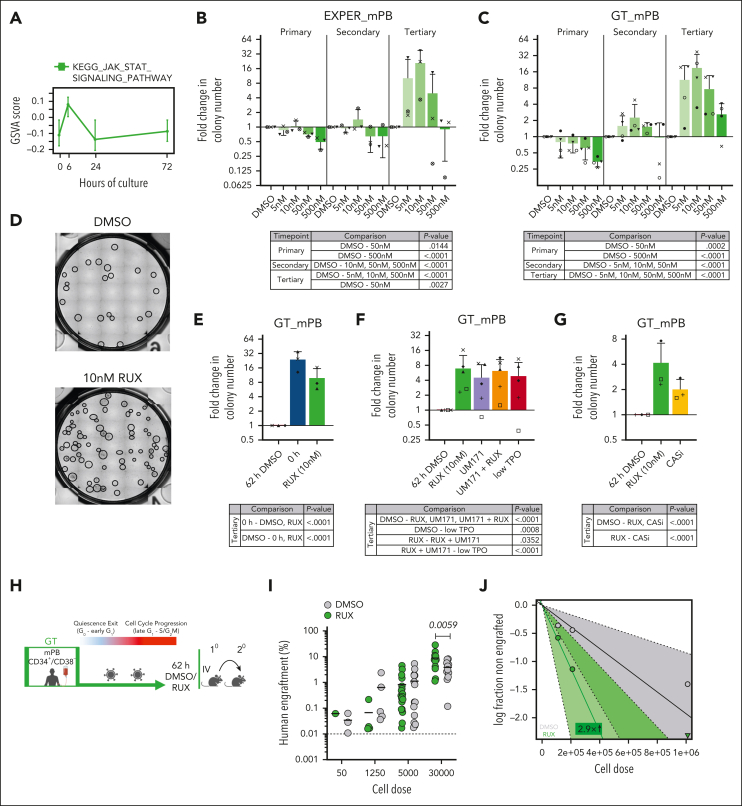
**RUX treatment improves serial replating ability and self-renewal capacity of cultured HSCs.** (A) GSVA score of the Kyoto Encyclopedia of Genes and Genomes (KEGG) JAK/STAT signaling pathway gene-set (same as Figure 2H). The GSVA score was calculated per single cell with the line indicating the median and the upper and lower whiskers indicating the 25th and 75th percentile of expression. (B-C) Serial replating of human mPB HSC/MPPs (CD34^+^CD38^−^CD45RA^−^) cultured for 72 hours in EXPER conditions (B) or for 62 hours in GT conditions (C). Graphs (upper panels) show the fold change in the colony number in comparison with DMSO from primary (2 week), secondary (4 week), and tertiary (6 week) plating. There are n = 3 mPB biological replicates in (B) and n = 4 mPB biological replicates in (C); the individual donors are indicated by shapes. The mean ± SD are shown. Tables (lower panels) report the statistics from a generalized linear mixed-effects model (glmer) analysis performed with raw colony counts. Tukey corrected *P* values for pairwise comparisons with DMSO with *P* < .05 are shown (supplemental Table 12 contains all comparisons and raw data). (D) Representative image of wells from tertiary replating experiment of mPB HSC/MPPs cultured for 72 hours in EXPER conditions (upper panel) or for 62 hours in GT conditions (lower panel) treated with either DMSO (left) or 10 nM RUX (right). Circles indicate manually scored colonies. Images brightened by 17%. (E-G) Serial replating of human mPB HSC/MPPs cultured for 62 hours in GT conditions. Graphs (upper panels) show fold change in colony number in comparison with DMSO from tertiary (6 week) plating in the conditions indicated as follows: (E-G) RUX (10 nM); (E) 0 hours fresh HSC/MPPs; (F) UM171 (35 nM), low TPO (20 ng/mL); (G) the pan-caspase inhibitor (CASi) Z-VAD(OH)-FMK (100 nM); (E,G) n = 3 mPB biological replicates; (F) n = 5 mPB biological replicates. Individual donors indicated by shapes and matching across (E-G). The mean ± SD are shown. Tables (lower panels) report the statistics from the glmer analysis with fitting of raw colony counts. Tukey corrected *P* values for pairwise comparisons of EM means with *P* < .05 are shown (supplemental Table 12 contains all comparisons and raw data). (H) Workflow of in vivo transplantation of mPB CD34^+^CD38^−^ cells cultured in GT conditions for 62 hours with LV transduction and RUX (10 nM) or DMSO. Secondary transplantations were performed from whole BM of engrafted mice. (I) Graft size (percentage of human CD45^++^ and GlyA^+^) in the BM at 18 weeks post transplantation of mPB CD34^+^CD38^−^ cells cultured for 62 hours in GT conditions with RUX (10 nM) or DMSO. n = 6 biological replicates; the graph shows representative of n = 68 engrafted mice (n = 34 DMSO; n = 34 RUX). Two-way ANOVA with Sidak multiple comparisons was performed (30 000 cells DMSO vs 30 000 cells RUX; *P* = .005; all other doses DMSO vs RUX; *P* > .9). (J) The log-fraction plot of the limiting dilution model fitted to data in supplemental Table 14. Indicates the percentage of LTRC estimates from secondary transplantation experiments. Whole BM of engrafted mice from primary transplants was transplanted in NSG-SGM3 mice and analyzed 8 weeks posttransplantation (n = 1 experiment; n = 35 mice). Slope indicates the log-active fraction. The dotted line shows the 95% CI. Zero negative response indicated by triangle. Panel H was created with BioRender.com (license agreement UR26QKHL5H).

We next hypothesized that inhibition of signaling pathways induced at adaptation may reduce ex vivo HSC functional attrition. JAK/STAT signaling stood out as a potential candidate, because JAK/STAT target genes were transiently upregulated 6 hours after culture initiation (Figure 5A). Consistently, at 30 minutes of GT culture, mPB HSC/multipotent progenitors (HSC/MPP; CD34^+^CD38^−^CD45RA^−^) significantly increased the phosphorylation of STAT5, STAT3, and, to a lesser extent, STAT1 (supplemental Figure 6A-B; supplemental Table 11). STAT phosphorylation could be decreased by the addition of RUX, a US Food and Drug Administration (FDA) approved JAK inhibitor, to the culture (supplemental Figure 6B). To determine the effect of RUX treatment in HSC culture systems, we assessed the differentiative and proliferative output of RUX-treated mPB LT-HSCs cultured in MEM medium for 14 days. The absolute cell numbers per well decreased significantly with increasing doses of RUX (supplemental Figure 6C), but the proportions of erythroid progenitors or myeloid cells did not change significantly, particularly at concentrations ≤50 nM (supplemental Figure 6D). RUX treatment therefore decreased differentiation globally with no major impact on lineage commitment, suggesting it may maintain LT-HSCs ex vivo.

To test whether RUX addition ex vivo could improve the regenerative function of cultured HSCs, we performed serial replating of mPB HSC/MPPs in both GT and EXPER conditions as an in vitro surrogate for self-renewal. By tertiary replating, in which colonies are largely generated by LT-HSCs, substantial increases in the number of colonies were observed at low doses of RUX in both media and across all donors (GT, 3-fold to 38-fold increase; EXPER, 3-fold to 37-fold; Figure 5B-D; supplemental Table 12). At 500 nM RUX, the positive effect was dampened or abrogated, likely because of decreased proliferation (supplemental Figure 6C) and potential off-target inhibition of tropomyosin receptor kinase B (TRK-β) activity.^52^^,^^53^ The number of colonies produced by 10 nM RUX-treated HSC/MPPs was still significantly lower than those generated by uncultured (0 hour) HSC/MPPs (Figure 5E; supplemental Table 12), indicating that RUX reduces the loss of functional HSCs induced during ex vivo adaptation instead of expanding the absolute number of initial HSCs.

Given the clinical need to develop robust human HSC expansion methods ex vivo, we performed serial replating to benchmark RUX action against previously reported strategies. In this assay, RUX (10 nM) treatment generated significantly higher numbers of tertiary colonies than treatment with UM171 (35 nM), a pyrimidoindole derivate in clinical trials for HSC expansion applications^54^, ^55^, ^56^ (RUX, 5-fold to 16-fold when compared with DMSO; UM171, 0.7-fold to 8-fold when compared with DMSO) (Figure 5F; supplemental Table 12). Interestingly, RUX and UM171 did not synergize in this assay (Figure 5F). RUX (10 nM) treatment also outperformed lowering TPO concentration, a strategy beneficial in human HSC expansion cultures^57^ (0.3-fold to 8-fold when compared with DMSO; Figure 5F). Finally, a pan-caspase inhibitor (Z-VAD(OH)-FMK, 100 nM), previously used for CD34^+^ expansion,^58^ only led to a modest increase in the tertiary replating ability of mPB HSC/MPPs (1.5-fold to 3-fold when compared with DMSO; Figure 5G; supplemental Table 12).

To formally assess whether ex vivo RUX treatment can increase the long-term in vivo repopulation ability of cultured LT-HSCs, we treated mPB CD34^+^CD38^−^ with 10 nM RUX for 62 hours and performed serial transplantations with an LDA design as in Figure 4 (Figure 5H). No difference in graft size was observed at 8 weeks in PB (supplemental Figure 6E). However, at 18 weeks after the transplant, the grafts of mice transplanted with high cell doses of RUX-treated CD34^+^CD38^−^ were significantly larger (*P* = .0059; Figure 5I; supplemental Figure 6F) than those of control mice. Although the %LTRC determined by LDA statistics were similar after primary transplantation (supplemental Table 13), we observed an approximately threefold increase in the %LTRC in RUX-treated CD34^+^CD38^−^ when compared with DMSO treatment after secondary transplantation (*P* = .0491; Figure 5J; supplemental Figure 6G; supplemental Table 14). These data collectively demonstrate that ex vivo JAK/STAT signaling inhibition via RUX improves HSC regenerative function.

## Discussion

Our study establishes the sequence of transcriptional and functional events that occur in human LT-HSCs during their first ex vivo division. Our data demonstrate that the early phases of LT-HSC adaptation to culture are critically detrimental to their function, irrespective of cell cycle progression. Our data also indicate the potential of targeting key pathways induced at adaptation, such as JAK/STAT signaling, to improve the safety and efficacy of any clinical protocol involving HSC culture.

It has long been assumed that division causes loss of LT-HSC function ex vivo. In vivo, there is a causative relationship between excessive proliferation of LT-HSCs and loss of self-renewal (reviewed in Johnson et al^59^). In addition, self-renewal capacity is enriched in vivo in G_0_ HSCs when compared with those in G_1_ or other cell cycle phases.^28^^,^^59^ Ex vivo, the G_0_/G_1_ fraction of cultured CD34^+^ is enriched in phenotypic HSC and contains the highest frequency of repopulating cells.^29^^,^^30^ Using a unique experimental system that efficiently and reversibly arrests LT-HSC progression past early G_1_ ex vivo, we demonstrate that cell cycle progression and division do not drive the sizable loss of LT-HSC repopulation capacity observed ex vivo over 62 or 72 hours. These findings allay concerns that gene editing strategies that require progression through the S phase may be associated with poor engraftment.^60^

Differentiation is tightly connected to cell cycle in most stem cell types, including HSCs,^47^, ^48^, ^49^^,^^59^^,^^61^, ^62^, ^63^ in which CDKs and cyclins control HSC fate regulators through direct phosphorylation or chromatin binding.^64^, ^65^, ^66^, ^67^ We observe that temporary cell cycle blockade dampens or delays the upregulation of myelo-erythroid lineage specification gene signatures, indicating that cell cycle progression facilitates the establishment of LT-HSC differentiation programs. HSC self-renewal and lineage commitment are controlled independently in vitro and in vivo in mice.^68^, ^69^, ^70^ Our study of cultured human HSCs further supports this concept by temporally uncoupling loss of repopulation capacity (occurring pre-Rb phosphorylation) from establishment of differentiation (occurring post-Rb phosphorylation).

Given the extensive transcriptional rewiring that occurs within 24 hours of culture, HSC loss of function in this time window is certainly multifactorial. Adaptation, through its characteristic transient expression dynamics and high intercellular variability of stress-related genes, likely purges functional HSCs through differentiation or death, akin to the preferential culling of unfit HSCs that occurs in vivo in response to oxidative, genotoxic, and proteostatic damage.^10^, ^11^, ^12^, ^13^^,^^26^^,^^71^^,^^72^ Early metabolic adaptations also likely contribute to later LT-HSC functional loss. In this study, we observed transient upregulation of the ceramide biosynthesis enzyme, DEGS1, the inhibition of which preserves LT-HSC repopulation capacity after ex vivo culture.^26^ Finally, we demonstrate that early induction of JAK/STAT signaling is detrimental to HSC function during culture.

The availability of large numbers of functionally fit HSCs remains a major limiting factor for most HSC transplantations and GT. Our data identify the FDA–approved JAK1/2 inhibitor, as an effective compound to mitigate loss of HSC function during ex vivo culture. Independent work from our groups demonstrate that tyrosine-unphosphorylated STAT5 (uSTAT5) is a critical regulator of self-renewal vs differentiation decisions in mouse HSCs.^73^^,^^74^ RUX shifts the uSTAT5/pSTAT5 ratio toward increased uSTAT5, promoting mouse HSC self-renewal.^74^ We propose this same mechanism underlies the beneficial effect of RUX on cultured human HSCs. This could be complemented by direct or indirect action of RUX on the MAPK,^75^ PI3K,^57^^,^^76^ NF-κB^77^ and TGF-β^78^ pathways. More broadly, by pinpointing irreversible HSC fate decisions to the first 24 hours of culture, our work identifies an early functional bottleneck and an untapped window of opportunity to preserve HSC function in any application that requires an ex vivo step. Although shortening HSC GT protocols may be useful,^79^ our data indicate that it should be complemented with strategies to minimize early HSC functional attrition. Similarly, targeting early HSC adaptation could further improve HSC expansion, complementing current protocols optimized to expand HSC over prolonged culture periods.^55^, ^56^, ^57^^,^^80^

Conflicts-of-interest disclosure: E.L. reports receiving research funds from GlaxoSmithKline. A.R.G. and J.L. report serving as a consulting for Incyte. The remaining authors declare that they have no competing interests.

## Supplementary Material

Supplemental Table 12

Supplemental Methods, Figures, Tables, and References
