# Supplementary material for: Adaptation to ex vivo culture reduces human hematopoietic stem cell activity independently of the cell cycle
Source: Blood. 2024 May 31;144(7):729–41. doi: 10.1182/blood.2023021426 (PMC7616366; doi:10.1182/blood.2023021426)
Supplement: Supplemental Methods, Figures, Tables, and References [file BLOOD_BLD-2023-021426-mmc9.pdf]

Supplemental data for Johnson et al.

- Supplemental Methods
- Supplemental Note 1
- Figs. S1 to S8
- Tables S1 to S18
- References

Supplemental Methods

Extended description of experimental methods

Human CB and mPB CD34$^+$ cell selection. CB Mononuclear cells (MNCs) were isolated from whole blood (diluted 1:1 in PBS) by Pancoll (PAN-Biotech) density gradient centrifugation at 500g for 25 minutes with the brake off. Red blood cells were lysed by incubation with Red Blood Cell Lysis Buffer containing ammonium chloride for 15 minutes at 4°C (BioLegend). CD34$^+$ selection was performed using AutoMACS cell separation technology following incubation with CD34$^+$ selection beads (Miltenyi Biotech) and FcR blocking reagent (Miltenyi Biotech) in PBS + 3% foetal calf serum (FCS) (PAN-Biotech) for 30 minutes at 4°C. CB CD34$^+$ cells were then cryopreserved with 20% DMSO (Sigma) in FCS (RBMI) at -150°C until further use. mPB CD34$^+$ cells were enriched by the CliniMacs Prodigy system (Miltenyi Biotech) using the automated LP-34 programme for 5 h and 45 minutes consisting of successive washes, CliniMACS® CD34 GMP MicroBeads (Miltenyi Biotec) incubation, immunomagnetic column selection, volume reduction and target cell elution using the TS310 tubing set (Miltenyi Biotec). mPB CD34$^+$ cells were cryopreserved in 10% DMSO (Sigma) in FCS (RBMI) at -150°C until further use.

Flow cytometry and Fluorescence Activated Cell Sorting. mPB and CB CD34$^+$ cells were thawed by dropwise addition of pre-warmed Iscove's Modified Dulbecco's Medium (IMDM) (Thermo Fischer Scientific) supplemented with 0.1mg/ml DNAse (Lorne Laboratories) and 50% FCS. Harvested cells were incubated in PBS + 3% FCS containing an antibody mix (**Table S15**; Panel A) for 20 minutes at room temperature (RT). Cells were washed and resuspended in PBS + 3% FCS for cell sorting on the BD FACS Aria Fusion (BD Biosciences) at the NIHR Cambridge BRC Cell Phenotyping hub. Populations were isolated in either a bulk or single cell manner depending on experimental purpose. Single cells were sorted with the single cell purity setting and index data recorded for all surface markers. Bulk cells were sorted with purity setting. Purity for all sorts was estimated at >95%. Previously defined phenotypic populations were sorted: (CD19$^-$ CD34$^+$CD38$^-$ for mPB) and LT-HSC (CD19$^-$CD34$^+$CD38$^-$CD90$^+$CD49f$^+$ for CB and mPB) [1] using the representative gates shown in **Fig. S7A**. For scRNA-Seq experiments performed following culture in mPB and CB, phenotypic populations were sorted at 0h and Zombie$^-$ (Live) fractions from each population were re-sorted post-culture at indicated time-points. Flow cytometry analysis was performed using the BD LSR Fortessa (BD Biosciences), BD LSR Fortessa X-20 (BD Biosciences), FACSCelesta (BD Biosciences), MACSQuant Analyzer 10 (Miltenyi Biotech) and in the case of high throughput analysis, the HTS Plate reader was used on the BD LSR Fortessa X-20. Cells were incubated in PBS + 3% FCS containing an antibody mix for 20 minutes at room temperature (RT). All antibodies and antibody panels are listed in **Table S15**.

Lentiviral vector (LV) production and purification. Third generation self-inactivating (SIN) lentiviral vectors expressing GFP under control of an EIF1α promoter were produced by transient transfection in HEK293T cells. HEK293T cells were cultured in an orbital shaker (37°C & 5% CO_2) and passaged until the optimum density ($1.5x10^6$ cells/ml) was achieved. Harvested HEK293T cells were transfected with four plasmids (incorporating Gag-Pol (pG3-SYNGP), Rev (pG3-REV), VSVG (pG3-VSVG) and the transfer plasmid containing an eGFP construct (pG3T)) in GMP grade Stem Cell Growth Medium (SCGM) (CellGenix) with Polyethylenimine PEIPro

(Polyplus) added to enhance transfection. Plasmids were obtained from the GSK research facility (Stevenage, UK). HEK293T cells were cultured (37°C & 5% CO_2) and 5mM of Sodium Butyrate (Sigma) was added to enhance transfection at 24 h. Cells were harvested at 72 h post transfection and LV containing medium was separated from cell debris by centrifugation (1,000g for 20 minutes at 4°C) with the supernatant clarified using 0.8µm and 0.45µm vacuum filters (Corning). LV particles were concentrated by ultracentrifugation (5,000g for 20 h at 4°C), then the supernatant was discarded and the pellet air dried. Pellets were resuspended in SCGM and stored at -80°C.

LV titration. CEM A3-04 cells at an optimum density ($1.5x10^6$ cells/ml) were resuspended in RPMI (Thermo Fisher) containing 8µg/ml protamine (Sigma) and plated at a density of $1.5x10^6$ cells/ml (density kept constant between wells). 10-fold serial dilutions of purified LV were added to wells and the cells incubated (37°C & 5% CO_2) for 2 h. Additional RPMI 1640 supplemented with 1% L-Glu (Life Technologies), 10% FCS (Life Technologies) and 1% Penicillin/Streptomycin (Pen/Strep) (ThermoFisher Scientific) was added to each well and the plates incubated (37°C & 5% CO_2) for a further 4 days. Harvested CEM cells were resuspended in PBS + 5% Human Serum Albumin (HSA) (Irvine Scientific) containing 1% 7-AAD (Biolegend). GFP positivity was analysed on the MACSQuant Analyzer 10 (Miltenyi Biotech). Vector titre (TU/ml) was calculated as = (Number of cells x %GFP+ x Dilution Factor) / Transduction volume (0.5 mL). Vectors of ≥1.19E+08 TU/ml were used for all experiments at multiplicity of infection (MOI) of 100-300. For scRNA-Seq experiments vectors of ≥4.44E+08 TU/ml were used.

LV transduction. Flow-sorted cells were cultured in GT media in a 96 well flat bottom plate coated with 33.3 µg/ml of Retronectin (Takara). The 62h protocol consisted of pre-stimulation in GT media (24h), transduction with a LV containing GFP (14h), an interim incubation in GT media without the vector (10h) and a second hit of transduction (14h).

Cell cycle assays. For time to first division assays, LT-HSCs were single cell sorted into 96 well u-bottom plates containing 100µl of indicated media per well either untreated (UNTR) or PD treated (200nM), centrifuged at 500g for 5 minutes and manually counted every 12h for 96h using a light inverted microscope. For phoshoRb stainings, cells were harvested from culture at indicated time-points and fixed with neat Cytofix/Cytoperm (BD Biosciences) for 10 minutes at RT. Cells were washed with 1x Permwash (BD Biosciences) and stained overnight with anti-Rb (phospho S807/S811, conjugated to Alexa647 fluorochrome) (Cell Signalling Technologies). Cells were again washed with 1x Permwash and stained with 0.5µg/ml of DAPI (ThermoFisher Scientific) for 15 minutes. Cells were resuspended in PBS + 3% FCS for flow cytometry analysis. DAPI was recorded on a linear scale and samples analysed at ≤ 30 events/second. For Ki-67 staining, cells were harvested from culture at indicated time-points and fixed with neat Cytofix/Cytoperm (BD Biosciences) for 10 minutes at RT. Cells were washed with 1x Permwash (BD Biosciences) and stained with anti-Ki-67 (conjugated to FITC fluorochome) (BD Biosciences) for 20 minutes at room temperature. Cells were again washed with 1x Permwash and stained with 0.5µg/ml of DAPI (ThermoFisher Scientific) for 15 minutes. Cells were resuspended in PBS + 3% FCS for flow cytometry analysis. DAPI was recorded on a linear scale and samples analysed at ≤ 30 events/second.

Cell size measurements. 100 CB LT-HSCs per well were sorted into a 384 well plate containing MEM media (see Methods) either UNTR or PD treated (200nM). Cell images were taken every 24 h in bright field (20x magnification) on a Leica DMI300 B microscope using the MetaMorph Microscopy Automation and Image Analysis Software. Cell size was analyzed using ImageJ measuring 25 cells/well and expressed as cell diameter (μm).

Mitochondrial activity assay. CB LT-HSCs were cultured in EXPER media, harvested at indicated time-points and stained with the cationic dye Tetramethylrhodamine (TMRM) (100nM) (Life Technologies) which accumulates in active mitochondria for 40 minutes at 37°C + 5% CO_2. Cells were washed and resuspended in an appropriate volume of PBS + 3% FCS for flow cytometry analysis.

Apoptosis assay. mPB LT-HSCs were cultured in GT media and CB LT-HSCs were cultured in EXPER media. Cells were harvested at indicated time-points, washed and resuspended in fridge cold PBS containing Annexin-V/PE (1/20) (BD Biosciences) and 7-AAD (1/20) (BD Biosciences) for 15 minutes at RT. 1X Annexin-V binding buffer (BD Biosciences) was added before immediate flow cytometry analysis.

Intracellular Flow Cytometry

mPB HSC/MPPs ($CD34^{+}CD38^{-}CD45RA^{-}$) were isolated and plated into 96-well plates at 2,000 cells per well in 50μl of serum free, cytokine free GT media with RUX (10nM/50nM Selleckchem) or DMSO (Sigma). Plates were incubated at 37°C for 1 h, and complete GT or EXPER media (see above) with RUX (10nM/50nM, Selleckchem) or DMSO (Sigma) was added for 30 minutes. No RUX, DMSO or cytokines were added to the unstimulated (UNSTIM) controls. As a positive control for maximum JAK/STAT activity, cells were plated in STIM conditions: GT media supplemented with TPO (200ng/ml), SCF (600ng/ml), Flt-3L (600ng/ml), IL-3 (120ng/ml), IL-6 (120ng/ml) and IFNγ (100ng/ml). After 30 minutes all samples were fixed with neat Cytofix/Cytoperm (BD Biosciences) for 10 minutes at RT. Fixed cells were washed with 1x Permwash (BD Biosciences) and stained with anti-pY694/Y699 STAT5 BV421 antibody (BD Biosciences), anti-pY701 STAT1 AF488 antibody (BD Biosciences) and anti-pY705 STAT3 PE antibody (BD Biosciences) on ice for 1h before immediate flow cytometry analysis. All antibodies and antibody panels are listed in **Table S15**.

Single cell MEM differentiation assay. Cells were single cell sorted into 96 well flat bottom plates containing medium facilitating the growth of myeloid (My), megakaryocyte (Meg), erythroid (Ery) and lymphoid (Lym; only natural killer (NK) cells supported) colonies, termed "MEM" media. MEM media: StemPro base media supplemented with Nutrients (0.035%), Pen/Strep (1%), L-Glu (1%), human LDL (50ng/ml), SCF (100ng/ml), Flt-3L (20ng/ml), TPO (100ng/ml), EPO (3 units/ml), IL-6 (50ng/ml), IL-3 (10ng/ml), GM-CSF (20ng/ml), IL-11 (50ng/ml), IL-2 (10ng/ml) and IL-7 (40ng/ml). All cytokines from Miltenyi/Peprotech except EPO (Janssen). Cells were harvested at day 21 of culture into a 96 well u-bottom plate, stained with an antibody mix (**Table S15**, Panel B) for 20 minutes at RT and washed (100μl per well of PBS + 3% FCS) before high-throughput flow cytometry analysis of colony size, colony output and clonogenic efficiency ((number of colonies generated / total single cells plated) * 100). A true

colony was defined as having ≥30 cells in ($CD45^+$ & $GlyA^+$) gates. Colonies were further assigned to a colony type as follows: Myeloid: ≥30 $CD45^+CD11b^+$ cells; Monocyte: ≥30 $CD45^+CD14^+$ cells; Granulocyte: ≥30 $CD45^+CD15^+$ cells; Lymphoid (NK only): ≥30 $CD45^+$ $CD56^+$ $CD11b^-$ cells. Undifferentiated colonies were determined as having ≥30 cells in ($CD45^+$ & $GlyA^+$) gates but no lineage assignment. Representative gates for analysis are shown in **Fig. S7B**.

Bulk LT-HSC differentiation assay with RUX. 250 mPB LT-HSCs were sorted into 96 well flat bottom plates containing MEM media. After 2.5h in culture, RUX (5nM, 10nM, 50nM, 100nM or 500nM, Selleckchem) or DMSO vehicle control was applied to cultures. Cells were harvested at day 14 of culture into a 96 well u-bottom plate, stained with an antibody mix (**Table S15**, Panel F) for 20 minutes at RT and washed (100µl per well of PBS + 3% FCS) before high-throughput flow cytometry analysis of colony size and lineage output. Representative gates for analysis are shown in **Fig. S8**.

Serial colony replating: mPB HSC pool cells ($CD34^+CD38^-CD45RA^-$) were isolated by flow cytometry and seeded at 400 cells per well in 96 well plates and cultured in GT, GT media with low TPO (20ng/ml) or EXPER media. 2 h after cells were placed into culture, RUX (5nM, 10nM, 50nM or 500nM, Selleckchem), pan-Caspase inhibitor (Z-VAD(OH)-FMK, Cayman Chemical; 100nM), UM171 (StemCell Technologies; 35nM) or DMSO (Sigma) was added to the media. After 72h of culture, cells were harvested and washed with FACS buffer (PBS + 3% FCS). Cells from each condition were split into two dilutions: either containing 20% or 80% of cells. These two dilutions were placed into H4034 Methocult medium (Stemcell Technologies) supplemented with 10ng/ml Flt3-L (Peprotech), 10ng/ml IL-6 (Peprotech) and 10U/ml Penicillin-Streptomycin (ThermoFisher Scientific) and split into duplicate wells (2 x 10% cell suspension and 2 x 40% cell suspension) of a 6 well plate. After 14 days, the number of colonies in each condition was counted using the Stemvision analyser (Stemcell Technologies) and then manually counted to adjust StemVision automated analysis (primary plating analysis). After counting, the duplicate well contents were harvested and washed with FACS buffer (3%FBS in PBS) before replating. The harvested cells were again split into two dilutions (either containing 20% or 80% of cells) and placed into H4034 Methocult (Stemcell Technologies) with added cytokines and Penicillin-Streptomycin and were then split into duplicate wells. After a further 14 days, the colonies were again counted by Stemvision followed by manual adjustment (secondary plating analysis) and replated once again. After a further 14 days, the colonies were again counted (tertiary plating analysis) as before. Colony counts shown are from the replating with 80% of cells and assigned by manual image analysis.

Primary xenograft transplantation. All experimental cohorts were >11 weeks old. Only female cohorts were used for experiments involving NSG animals. For primary transplantation experiments, NSG mice were sub-lethally irradiated (2.4 Gy) 24 h prior to transplantation. For intrafemoral (IF) injections, mice were anesthetized with isoflurane and transplanted with the indicated cell dose in PBS + 0.1% Pen/Strep (ThermoFisher) (25µl). Following transplantation, mice were injected subcutaneously with the analgesic buprenorphine (Animalcare) at 0.1mg/kg. For intravenous injection, mice were transplanted with a cell suspension (max 150µl volume) in PBS + 0.1% Pen/Strep by tail vein injection. For all xenograft experiments involving cell culture, injected cell doses at time-points are representative of the cell count at the time of sort (0 h). Mice were bled at 8 weeks post-transplant from the tail vein. 5 drops of blood were collected and Pancoll

(PAN-Biotech) added to each sample. Density gradient centrifugation was performed at 500g for 25 minutes with the brake off. The MNC layer was collected and taken for antibody staining (**Table S15**; Panel C) for 20 minutes before washing and resuspension in PBS + 3%FCS. Mice were culled and bone marrow harvested 18-20 weeks for primary transplantation experiments. The femur and tibia bones from the two hind legs were taken and for IF injected mice, the injected femur was analysed separately. Bone marrow was stained in an antibody panel (**Table S15**; Panel C) for 20 minutes before washing and resuspension in PBS + 3% FCS for flow cytometry analysis.

Mice were considered engrafted if human cells (hCD45^{++} & GlyA^{+}) were ≥30 cells and represent ≥0.01% of Singlets, and if ≥20 cells were present in any lineage determination gate (**Fig. S7C**). Grafts were considered GFP^{+} if ≥30 cells were present in the GFP^{+} gate (**Fig. S7C**).

Secondary xenograft transplantation. Both male and female animals were used for experiments involving NSG-SGM3 animals. For secondary transplantation experiments in the EXPER culture system, NSG-SGM3 mice were irradiated using 2.25 Gy 24 h prior to transplantation and primary BM samples were thawed in X-VIVO 10 media (Lonza) + 50% FBS (Wisent) supplemented with Dnase (100 μg/ml, Roche). Viable (SytoxBlue^{-}) (ThermoFisher Scientific) human CD45^{++} cells were sorted (**Table S15**; Panel D) on the Aria Fusion (BD Biosciences). Cells were pooled based on condition and intrafemorally injected in three doses. Mice were culled at 8 weeks, bone marrow harvested, stained in an antibody panel (**Table S15**; Panel E) and analysed by flow cytometry with the same methodology as in primary transplantation experiments. Secondary transplantation experiments for EXPER conditions were kindly performed at UHN, Toronto.

For secondary transplantations of experiments in the GT culture system, primary mouse BM was thawed by dropwise addition of pre-warmed Iscove's Modified Dulbecco's Medium (IMDM) (Thermo Fisher Scientific) supplemented with 0.1mg/ml Dnase (Lorne Laboratories) + 50% FCS. Cells were counted and injected in three doses by IV injection (max 150μl volume) in PBS + 0.1% Pen/Strep. Mice were culled at 8 weeks, bone marrow harvested, stained in an antibody panel (**Table S15**; Panel C) and analysed by flow cytometry with the same methodology as in primary transplantation experiments. Mice were considered engrafted with the same criteria as for primary xenograft transplantation experiments.

Smart-Seq2 adapted protocol. Single cell RNA-Sequencing (scRNA-Seq) libraries were prepared using an adapted Smart-Seq2 protocol[2]. A cell lysis buffer was prepared of 20U/μl SUPER-In RNase inhibitor (Thermo Fisher Scientific) and 0.4% Triton-X100 (ratio of 1:19). The lysis buffer was added to a mix of DTT (Thermo Fisher Scientific) (final concentration of 0.57mM), dNTPs (Invitrogen) (final concentration 0.11mM) and nuclease free water (Thermo Fisher Scientific) and 4μl aliquoted per well in a 96 well PCR plate and stored at -80°C. Upon thawing and single cell sorting, an annealing mix was added (containing External RNA Consortium Controls (ERCC) RNA Spike-In mix diluted 1:3x10^{6} (Thermo Fisher Scientific)), 10μM Oligo-dT30 VN (Thermo Fisher Scientific) and nuclease free water (ThermoFisher Scientific) and annealing performed (72°C for 3 minutes). The plate was briefly centrifuged (1,000g for 30 seconds at 8°C) to collect liquid at the bottom of the well, the reverse transcription master mix added (containing 10U/μl SMARTScribe reverse transcriptase (Thermo Fisher Scientific), 1U/μl SUPER-In RNase inhibitor (Thermo Fisher Scientific), 2μM Template Switching Oligo (TSO), 5X First Strand Buffer (Thermo Fisher Scientific) and nuclease free water

(Thermo Fisher Scientific)) and reverse transcription performed (~2.5 h). The plate was again briefly centrifuged (1,000g for 30 seconds at 8°C), the PCR master-mix added (containing final concentration of 166nM IS-PCR primer, 2X KAPA HiFi HotStart ReadyMix (Roche) and nuclease free water) with 23 PCR cycles performed to account for the low RNA content of quiescent HSCs (~3 h). Plates were stored at -20°C until PCR purification. RT Ampure XP Beads (Beckmann Coulter) were mixed and incubated with PCR product (1:0.6/0.7 ratio) and PCR purification performed as in [2], to remove fragments of a small, suboptimal length. The Biomek FXP Automated Workstation was used for experiments involving >3 plates. The quality of material was checked using the Agilent High Sensitivity DNA kit (Agilent) following manufacturer's instructions. The Quant-iT PicoGreen dsDNA kit and reagents (Thermo Fisher Scientific) were then used to determine the concentration of DNA (triplicate values per plate per cell condition) and dilution plates created (using elution buffer as diluent) for all samples to achieve 0.1-0.15ng/µl. Tagmentation was performed (55°C for 10 minutes) using the Nextera XT DNA library preparation kit (Illumina) mixing 2.5µl Tagment DNA Buffer, 1.25µl Amplicon Tagment Mix and 1.25µl of DNA per sample. The Tn5 transposase was stripped using the NT buffer (Illumina) (1.24µl per sample) and a PCR mix added (containing 3.74µl of NPM mix (Illumina) and 1.24µl of appropriate i7 and i5 primers (Illumina) per sample) with PCR performed (~30 mins) to amplify adapter-ligated sequences. PCR product purification was performed in two successive steps using RT Ampure XP beads (1:0.5, 1:0.3) to yield final average amplicon lengths of ~400-700 bp.

scRNA-Seq experimental design. Two scRNA-Seq datasets were generated in this study: a time-course of LT-HSCs cultured in EXPER conditions (Dataset 1), and a time-course of LT-HSC cultured in GT conditions (Dataset 2). PD treated (200nM) LT-HSCs were included in both datasets at indicated time-points. Dataset 1 was acquired over 2 independent experiments (Batch 1 and 2) using LT-HSCs isolated from CB samples from 2 independent pools of male donors at the time-points and conditions indicated in **Table S16**. Dataset 2 was acquired over 4 independent experiments (Batches 1 to 4) using LT-HSCs isolated from mPB samples from 4 independent healthy male donors at the timepoints and conditions indicated in **Table S17**. UNTR and PD treated conditions were always paired at matched culture durations in the same batches.

Two integrations were performed in this study: i) all CB LT-HSCs from 0 h and UNTR timepoints of Dataset 1 (Integration 1); ii) all single cells of Dataset 1 and Dataset 2 (UNTR and PD treated; Integration 2). Integration 1 was performed with 2 independent methods: i) Seurat 4 method; ii) Scanpy method. Integration 2 was performed with the Seurat 4 method. All bioinformatic methods described in extended description of bioinformatic methods below.

Statistical analysis. Analysis of the HSC frequency/Long term repopulating cell frequency (%LTRC) from transplanted populations was performed by using Extreme Limiting Dilution Analysis (ELDA) software (https://bioinf.wehi.edu.au/software/elda) taking into account the number of engrafted mice, the total mice used and the cell dose transplanted. For analysis of a statistical difference in the HSC frequency within two groups, a Chi-Squared test was performed within the ELDA software. For the analysis of *in vitro* serial replating data, raw colony counts per technical duplicates were average and rounded up. These colony counts (**Table S12**) were fitted to a generalized linear mixed-effects model (*lme4* library, *glmer* function in R, Poisson distribution) using donor as a random effect. EM means pairwise comparison was then performed with *emmeans*.

Flow cytometry data was analysed using FlowJo software (v10). For analysis of colony data derived from single sorted LT-HSCs, FlowJo v.10 gating statistics were exported and data further analysed in R Studio (v.1.2). Graphpad Prism (v9.3), python (v3.8.6) and R Studio (v1.2) were used for the creation of all plots. Statistical analysis between multiple groups was performed in Graphpad Prism or R Studio. Normality of data was deduced from the Shapiro-Wilks normality test. For statistical analysis between two groups a parametric test (Students t-test) or a non-parametric test (Mann-Whitney U-test) was performed. For analysis between multiple groups, an analysis of variance (one or two-way ANOVA) test was performed. All statistical tests were performed with a confidence interval of 95%.

Visualization. Data graphs were produced in R, Python or GraphPad Prism. Figures 4D (license agreement: KE26QKHW50), **5H** (license agreement: UR26QKHL5H) and **S4B** (license agreement: GS26QMICN2) were created with BioRender with appropriate licensing options.

Extended description of bioinformatic methods

scRNA-Seq quality control. Read alignment was performed using GSNAP [3] against Ensembl genes and initial quality control (QC) was performed by FastQC [4]. The count matrix was generated by HTSeq [5]. Additional QC was then performed in the bglab package [6] using the determined thresholds shown in **Table S18**, yielding the number of cells passing QC reported in **Table S16** for Dataset 1 and **Table S17** for Dataset 2.

Seurat 4 pipeline for batch correction and batch/dataset integration. Seurat objects were created using the Seurat package (v4)[7]. Genes from raw counts were filtered if not detected in >3 cells. The function SCTransform was used to perform normalisation and variance stabilisation, the mitochondrial genes percentage was regressed out. All other parameters were left as default if not mentioned specifically. For cell cycle regression, S and G_2-M cell cycle scores were calculated using the CellCycleScoring function on the object following SCTransform. The values of G_2-M scores were subtracted from the values of S scores resulting in the difference of the cell cycle scores. SCTransform was applied on the Seurat object again regressing the mitochondrial genes percentage and the difference of the cell cycle scores. For batch/dataset integration, Seurat objects to be integrated or batch corrected were curated into a list. 3,000 features were selected with the function SelectIntegrationFeatures. The list of objects was prepared to integrate using the function PrepSCTIntegration. FindIntegrationAnchors function was used to find a set of anchors for integration with the k.filter parameter set to 100. Integration was performed with the function IntegrateData. PCA was computed on the batch corrected/aligned Seurat objects with the RunPCA function. UMAPs were computed using the RunUAMP function with the dims parameter at 1:30.

Pseudotime analyses. The Monocle3 package (version 1.2.9) [8] was used to determine the pseudotime ordering of samples. Seurat integrated objects were converted to Monocle3 objects with the function as.cell_data_set from the package seurat-wrappers [9]. The cluster_cells function was run with the parameter reduction_method set to 'UMAP'. The principal graph was generated with the function learn_graph (with use_partitition parameter set to TRUE for Integration 1 and set to FALSE for Integration 2). Cells were ordered on the principal graph using the order_cells function, with a manually chosen root cell.

Scanpy normalisation and processing of counts. Raw counts of both batches from Dataset 1 were processed using the Scanpy package (version 1.4.5.1) [10]. Scanpy anndata objects of both batches were created and concatenated. The function filter_genes was run with parameter min_cells=3. After filtering, 33,774 genes were left. Cells were normalised with the function normalize_total with parameter target_sum=1e4. Counts were log transformed with a pseudocount of 1 added to mitigate the mean-variance relationship using the log1p function, to reduce skewing of data and account for drop-outs. Batch effects were regressed out with the function combat, using the time-point as the covariates. The scanpy combat function is a wrapper function for the combat package (https://github.com/brentp/combat.py). Highly variable genes were selected with the function highly_variable_genes, which implements the works of [11]. The parameters for the highly_variable_genes function were as follows: min_mean=0.05, max_mean=13, min_disp=0.1, max_disp=3. The anndata object was subset with 9,212 highly variable genes. Principal component analysis was performed using the pca function in the Scanpy package with default parameters. Nearest neighbours for the cells were selected using the bbknn package (version 1.3.7) [12]. The function bbknn_pca_matrix was used with the PCA matrix calculated by Scanpy and the batch information as inputs, parameters were set as follow: approx=False, metric= 'euclidean'. The function umap was used to find the UMAP representation of the data, the parameter n_components=3.

Differential expression and definition of "ex vivo modulated genes". Genes from the raw count matrix were filtered out if expressed in < 4 cells. Filtered raw counts were used to perform differential expression analysis with the R package DESeq2 (version 1.36.0) [13]. Batch effects were accounted for in the model where applicable. For Integration 1, the union of all differentially expressed genes in each pairwise time-point comparison was curated, generating a list of 10,010 genes, herein referred to as "*ex vivo* modulated genes" (**Table S5**). A variance stabilizing transformation was performed in the DESeq2 package from the differential expression analysis. Batch effects were removed on the variance stabilised matrix using the limma package correction (version 3.52.2) [14]. These VST batch corrected values were used for visualisations of gene expression in violin plots and as input for degPatterns.

Gene set enrichment and variation analysis. Gene Set Enrichment Analysis (GSEA) (v4.2) [15] was performed against the c2 curated pathway database using a pre-ranked list by the stat value (value of the Wald test statistic) from the DESeq2 output specifying 10,000 permutations. Gene-Set Variation Analysis (GSVA) was also performed [16] against c2 curated pathways using the normalised, batch corrected count matrix generated by Scanpy (parameters min.siz=10, max.sx=500). GSEA and GSVA were also performed with curated signatures. All gene signatures were created using the top 100 differentially expressed genes contrasting previously reported populations. Signatures were curated from $CD34^{lo}CLEC9A^{hi}$ (Subset1) and $CD34^{hi}CLEC9A^{lo}$ (Subset 2) [17] and from human dormant and activated HSC populations [18].

Identification of patterns of gene expression along the time course. The degPatterns function from the DEGreport package [19] was used to group genes based on expression pattern, inputting VST limma corrected matrix values for *ex vivo* modulated genes. The time parameter was set to the time-point and eachStep=TRUE. 8,966 genes remained after degpatterns filtering, removing clusters with <15 genes. A distance matrix was generated from all pairwise comparisons between time-points and hierarchical clustering was performed specifying 13 clusters. Upon visual

observation 2 of the 13 clusters were manually split, generating 15 clusters which fit the dataset without showing redundancy in expression pattern (clusters were renamed 1-15 accordingly).

To group GSVA biological pathway patterns based on expression trend, the degPatterns function from the DEGreport package [19] was again used, inputting the GSVA score matrix, with the parameter time set to the incubation time of the samples and eachStep=TRUE. 4,367 out of 4,596 pathways remained after filtering and 13 clusters again were specified to fit the dataset. Upon visual observation, 6 of 13 clusters of the GSVA score patterns were manually split further, resulting in 19 clusters with no redundancy in expression pattern observed (clusters were renamed 1-19 accordingly).

scEntropy measurements. To measure the transcriptomic order of single cells in the dataset, the package scEntropy was used [20]. The scEntropy per cell is defined as the difference between the cell of interest and an intrinsic reference value calculated by the package (parameter option =RCSA). Scanpy was used to pre-process raw counts from both batches separately and cells were normalised by $1x10^4$ reads (normalize_total function with parameter target_sum=1e4). The scEntropy value for single cells was calculated. To account for batch effects, the difference in the mean entropy values of batch 1 and batch 2 was subtracted from each entropy value of batch 1.

Bayesian modelling of gene expression and over-dispersions to measure expression variability. The BASiCS package (version 2.2.4) [21] was used to perform Bayesian modelling of the counts. The amount of ERCC molecules present together with each cell was calculated from the concentration of the ERCC mix added and the information for the ERCC mix acquired online ([https://assets.thermofisher.com/TFS-Assets/LSG/manuals/cms_095046.txt]). Raw counts were filtered to average reads per million > 20, resulting in 8,464 genes. BASiCS objects were created with the filtered counts, ERCC information and batch information. The objects were created independently per time-point. For the 24 h time sample, one cell was not included for the creation of BASiCS object due to a low ERCC quantity present. Bayesian inference of the parameters of gene count distributions were performed using the function BASiCS_MCMC. The parameters for the BASiCS_MCMC function which calculate residual over dispersion (ε) were as follow: N=10000, Thin=10, Burn=1000, WithSpikes=TRUE and Regression=TRUE. To identify residual over-dispersed genes for all time-points, the function BASiCS_TestDE was used that compares the value of residual over-dispersion and performs statistical testing between 2 samples. Genes that are differentially over-dispersed based on the residual over-dispersion value were curated for each time-point. To calculate maximally variable genes (MVG) at each time-point, genes that were differentially variable in multiple pairwise comparison were assigned to the time-point with the highest residual over-dispersion value.

Correlations of median expression. For EXPER_CB conditions, the median expression value for *ex vivo* modulated genes plus genes differentially expressed in PD treated comparisons (genes changed in 0h vs 72h PD, 72h PD vs 72h UNTR, 0 vs 24h PD, 24h PD vs 24h UNTR) (n=10,903 genes total) was plotted for each comparison and the Pearson's correlation coefficient calculated (95% CI; mean value shown). For GT conditions, the median expression value for the sum of genes changed in 0h vs 62h UNTR, 0h vs 62h PD and 62h PD vs 62h UNTR, was used (5,469 genes) and each cell at each time-point was compared. All gene lists can be found in **Table S5.**

Supplemental note 1

An increasing body of work is identifying transcriptional and functional heterogeneity within the human LT-HSC pool [17,18,22–24], indicating the potential presence of multiple subpopulations at the 0 h time-point. 0 h LT-HSCs were therefore further split into 2 clusters by k means clustering (**Fig. S2D)**. Differential gene expression was performed with DESeq2, followed by GSEA (**Table S3)** and GSVA analyses. One cluster expressed significantly higher levels of genes involved in hypoxia regulation (**Fig. S2E**) than the other and displayed enrichment for the most dormant [18] and multipotent [17] LT-HSCs gene signatures **(Fig. S2F)**. This cluster was denoted “0 h-early” and was subsequently used as pseudotime origin (**Fig. S2G**). In the other cluster, named “0 h-late”, we found higher levels of oxidative phosphorylation genes compared to “0 h-early” LT-HSCs (**Fig. S2H;** *p=0.0580*) and significant enrichment of gene signatures related to a myelo-lymphoid restricted self-renewing HSC subset [17] (**Fig .S2F**). In summary, our scRNA-seq strategy supports recent literature identifying transcriptional heterogeneity within the quiescent human LT-HSC fraction [17,22–25].

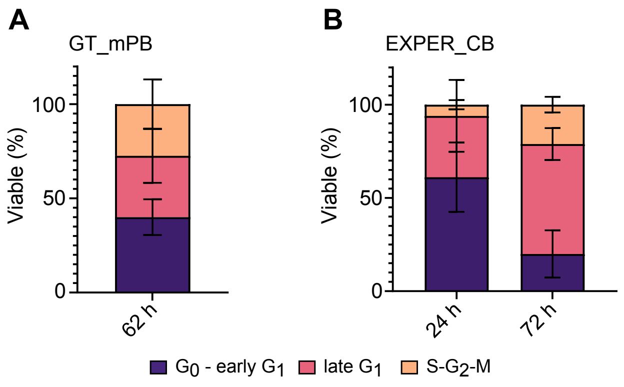

Fig. S1. Cell cycle status of LT-HSC during GT and EXPER culture

(A-B) Cell cycle phase assignment of LT-HSCs cultured in **(A)** GT_mPB and **(B)** EXPER_CB systems determined by Ki-67/DAPI flow cytometry analysis. n=3 independent experiments.

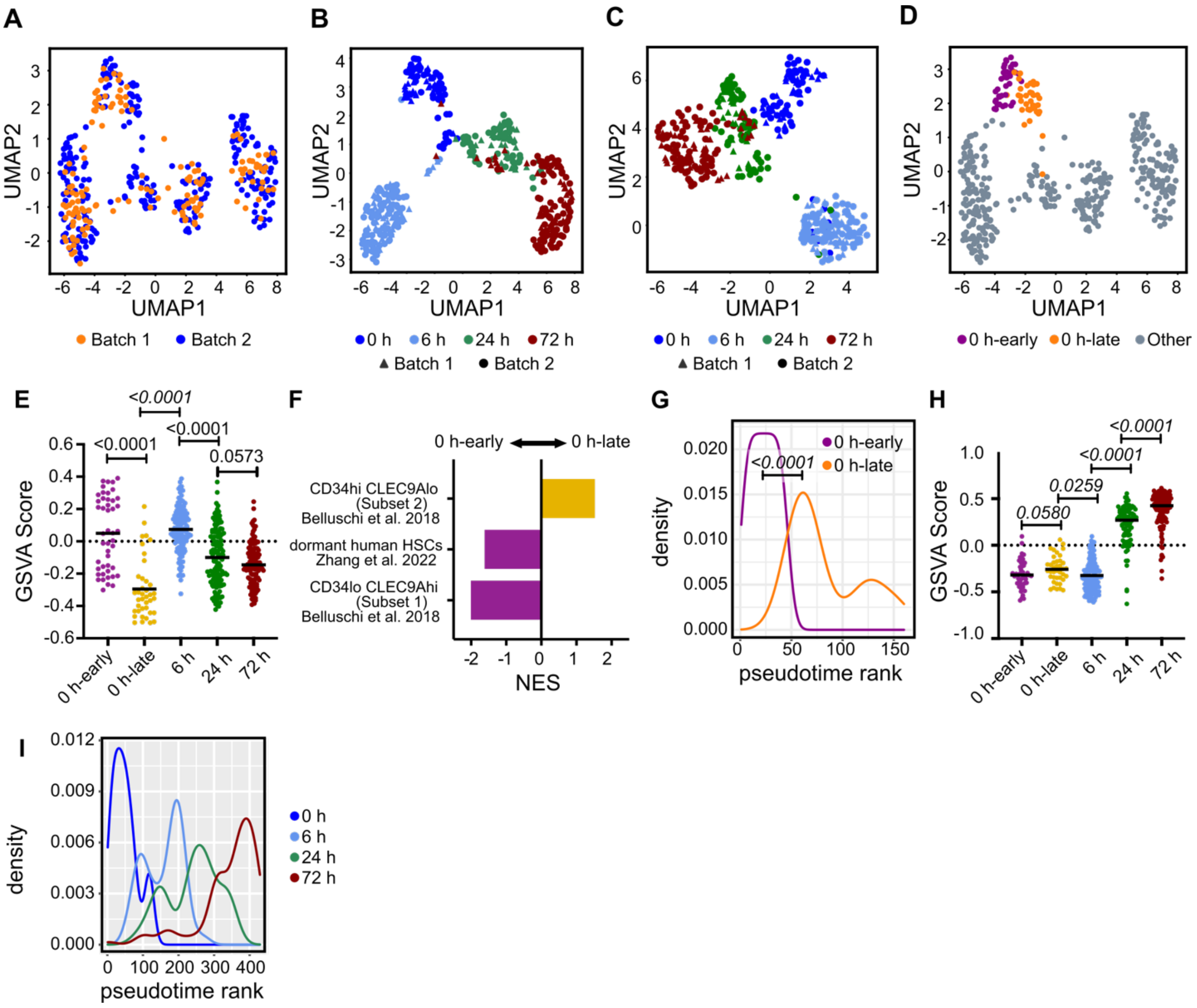

Fig. S2. Time course scRNA-seq data of cultured LT-HSCs and characterization of heterogeneity in quiescent CB_EXPER LT-HSCs

(A-D) UMAPs of 429 single CB LT-HSCs cultured in the EXPER system over a time-course of 0h, 6h, 24h and 72h. **(A)** UMAP coloured by experimental batch and generated using Seurat 4 pipeline following cell cycle regression. **(B)** UMAP colored by time-point with shape representing experimental batch and generated using Seurat 4 pipeline with no cell cycle regression. **(C)** UMAP colored by time-point with shape representing experimental batch and generated using Scanpy pipeline with no cell cycle regression. **(D)** UMAP as in (A) showing k=2 clusters defined within 0 h cells: 0h-early (purple, n=46 cells) and 0h-late (yellow, n=39 cells).

(E) GSVA scores per cell of "KRIEG_HYPOXIA_VIA_KDM3A" gene-set in the indicated conditions. Unpaired t-test.

(F) GSEA analysis comparing 0h-early and 0h-late LT-HSCs to curated signatures generated from dormant human HSCs [25], multipotent LT-HSCs ($CD34^{hi}CLEC9A^{lo}$; Subset 1) and myelo-lymphoid restricted LT-HSCs ($CD34^{lo}CLEC9A^{hi}$; Subset 2)[17], all comparisons *FDR < 0.05*.

(G) 2D Pseudotime rank plot of 0h-early and 0h-late. Wilcoxon rank sum test performed.

(H) GSVA scores per cell of "REACTOME_RESPIRATORY_ELECTRON_TRANSPORT_ATP_SYNTHESIS_BY_CHEMIOSMOTIC_COUPLING_AND_HEAT_PRODUCTION_BY_UNCOUPLING_PROTEINS" gene-set in indicated conditions. Unpaired t-test.

(I) 2D Pseudotime rank plot of LT-HSCs over time-course generated with no cell cycle regression applied.

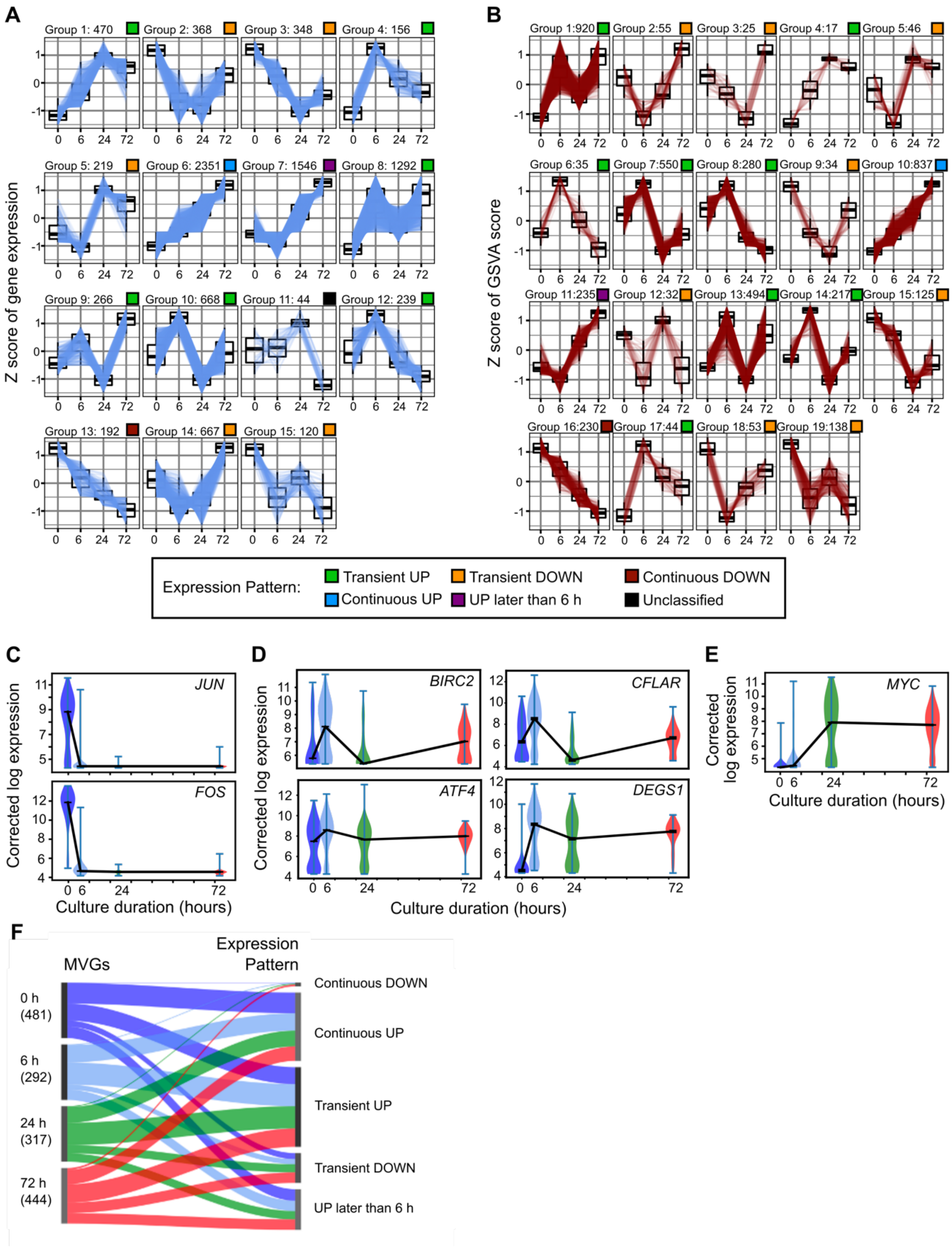

Fig. S3. Dynamic patterns of LT-HSC gene expression observed during *ex vivo* culture

(A-B) Output from DEG Report clustering of **(A)** gene expression patterns or (**B**) GSVA pathway patterns**.** **(A)** 15 gene expression patterns identified from 10,010 genes classified total; 8,966 genes after filtering. **(B)** 19 GSVA biological pathway patterns identified from 4,596 total GSVA pathways classified; 4,367 pathways after filtering. **Table S5**.

(C-E) Violin plot of corrected log expression values for selected genes. (**C**)AP-1 transcription factors *JUN* and *FOS*, (**D**) apoptosis master regulators *BIRC2* and *CFLAR*, regulators of proteostatic stress *ATF4* and *DEGS1*, and **(E)** transcription factor *MYC*. Line connecting median values between time-points.

(F) Sankey plot of maximally variable genes (MVG) at each time-point (left) matched to corresponding expression pattern (right) (total MVG at 0h = 853; 6h = 470; 24h = 605; 72h = 864 genes).

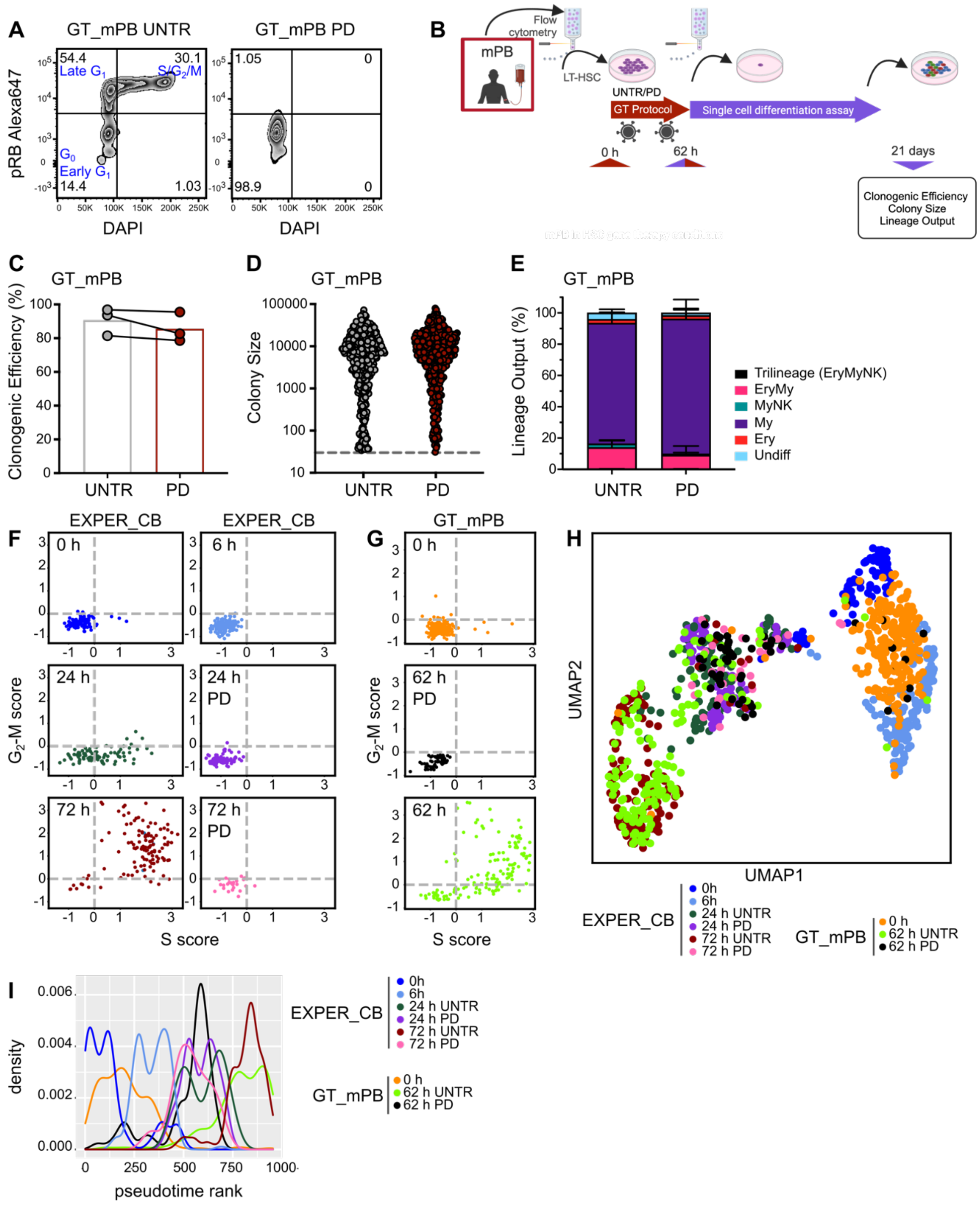

Fig. S4. Validation of reversible early G_1 arrest in LT-HSCs by PD treatment and the transcriptional effects cell cycle arrest

(A) Representative example of pRb/DAPI flow cytometry plot of alive UNTR (left) or PD treated (right) mPB LT-HSCs cultured for 62h in GT conditions.

(B) Workflow of GT protocol culture and single cell differentiation assay. mPB LT-HSCs were flow cytometry sorted in bulk and cultured in GT protocol conditions for 62 h including two rounds of LV transduction. Live (Zombie^{-}) cultured LT-HSCs were then single cell sorted and cultured for an additional 21 days in a single cell differentiation assay in media supporting the generation of Myeloid, Erythroid, Megakaryocytic and NK colonies (MEM). At day 21, colonies were analysed for clonogenic efficiency, colony size and lineage output. Created with BioRender (license agreement: GS26QMICN2).

(C-E) *In vitro* analysis of UNTR and PD treated colonies from GT_mPB LT-HSCs. **(C)** Clonogenic efficiency ((number of wells generating true colonies / number of wells plated)*100), (**D**) colony size distribution**,** and (**E**) types of colonies (lineage output, Ery = CD45^{-}GlyA^{+}; My = CD45^{+}CD14^{+}; NK = CD45^{+}CD56^{+}CD11b^{-})) of mPB UNTR/PD treated LT-HSCs and cultured in GT protocol followed by culture in single cell MEM differentiation assay. A colony is defined by ≥30 cells in CD45^{+} & GlyA^{+} gates (n=3 biological repeats, UNTR = 421 colonies and PD treated = 369 colonies).

(F) Transcriptional allocation of cell cycle status of UNTR/PD treated LT-HSCs cultured in EXPER_CB conditions (n=2 experimental batches; n=536 total cells).

(G) Transcriptional allocation of cell cycle status of UNTR/PD treated LT-HSCs cultured in in GT_mPB conditions (n=4 experimental batches; n=418 total cells).

(H) UMAP visualisation of scRNA-seq from 954 LT-HSCs from the indicated culture conditions (EXPER_CB: 536 single cells: GT_mPB: 418 single cells). No cell cycle regression applied.

(I) 2D Pseudotime density rank plot of LT-HSCs cultured in EXPER_CB and GT_mPB systems. No cell cycle regression applied. Number of cells as in **(H)**.

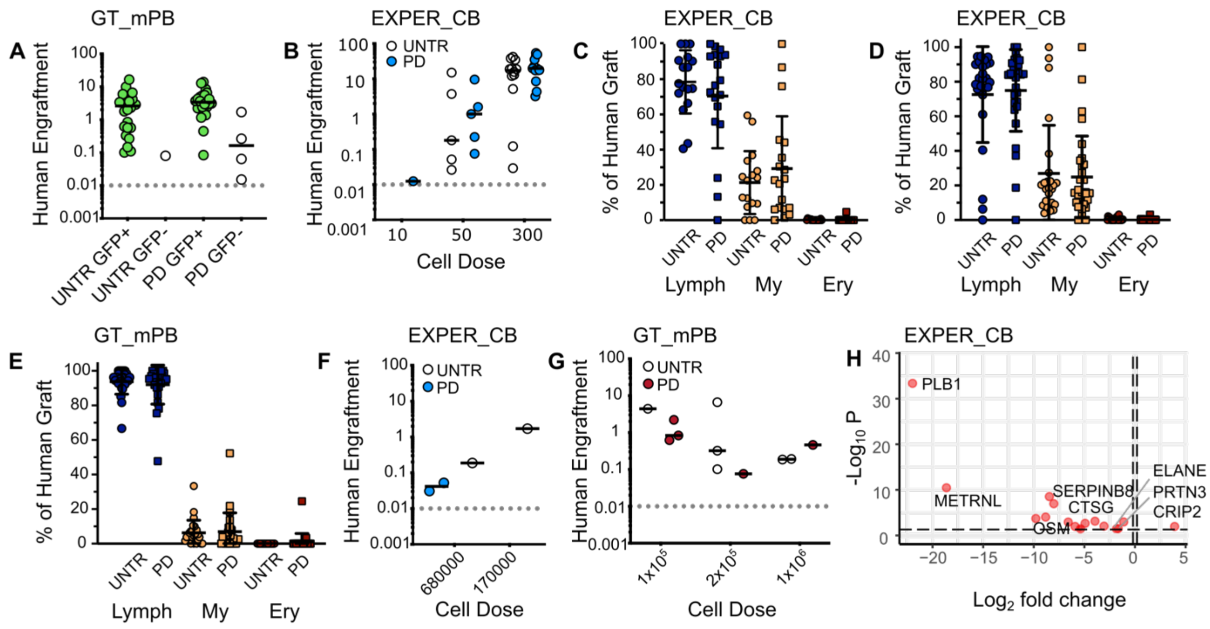

Fig. S5. PD treatment *ex vivo* does not affect engrafting capacity of LT-HSCs but alters the expression of genes linked to differentiation

(A) Graft size in mice engrafted with mPB CD34^{+}CD38^{-} split by GFP status (GFP^{+} graft defined as ≥30 cells in GFP^{+} gate) in primary mice transplanted with matched UNTR/PD treated cell doses. GFP^{+} grafts were observed in 24/25 mice engrafted with UNTR cells (96%) and 22/26 of mice engrafted with PD treated cells (85%). Mann Whitney test performed (PD GFP^{+} vs PD GFP^{-} *p=0.0024*). Dotted line: threshold of engraftment (CD45^{++}≥ 0.01 % and at least 30 cells recorded).

(B-C) Graft size (% of human CD45^{++} and GlyA^{+}) **(B)** and lineage output **(C)** at 18 weeks post transplantation of UNTR/PD treated LT-HSCs cultured for 24 h in EXPER system (n=3 independent experiments; only engrafted mice shown, n= 17 UNTR mice and n=19 PD treated mice) in primary mice transplanted with matched UNTR/PD treated cell doses. Two-way ANOVA with Sidak's multiple comparisons performed (50 cells UNTR vs 50 cells PD p=0.638; 300 cells UNTR vs 300 cells PD p=0.6380) (**B**). Lineage output: Lymphoid = CD45^{++}CD19^{+}; Myeloid = CD45^{++}CD33^{+}; Erythroid = CD45^{-}GlyA^{+}(**C**) Mann Whitney U test performed (Lymphoid UNTR vs Lymphoid PD p=0.666; Myeloid UNTR vs PD p=0.736; Erythroid UNTR vs PD p=0.186).

(D) Lineage output at 18 weeks post-transplantation of UNTR/PD treated CB LT-HSCs cultured for 72 h in EXPER system (n=5 independent experiments; only engrafted mice shown, n=30 PD mice, n = 25 UNTR mice). Mann Whitney U test performed (Lymphoid UNTR vs PD p=0.864; Myeloid UNTR vs PD p>0.9; Erythroid UNTR vs PD p=0.129).

(E) Lineage output at 18 weeks post-transplantation of UNTR/PD treated mPB CD34^{+}CD38^{-} cultured for 62 h in GT system. (n=3 independent experiments; only engrafted mice shown, n=26 PD mice, n = 25 UNTR mice) Mann Whitney U test performed (Lymphoid UNTR vs PD p=0.873; Myeloid UNTR vs PD p=0.746; Erythroid UNTR vs PD p=0.490).

(A-E) Raw data available in **Table S1**.

(F-G) Graft size in secondary transplantation experiments from primary recipients initially engrafted with **(F)** UNTR/PD treated CB LT-HSCs cultured for 72 h in EXPER system (only engrafted mice shown; 2 UNTR, 4 PD; n=1 experiment) and **(G)** UNTR/PD treated mPB $CD34^+CD38^-$ cells cultured for 62h in GT system (only engrafted mice shown, UNTR = 6 mice; PD = 5 mice; n=1 experiment).

(H) Volcano plot of GMP signature genes differentially expressed (*FDR<0.05*) between UNTR/PD treated EXPER_CB LT-HSCs cultured for 72h (17 genes shown).

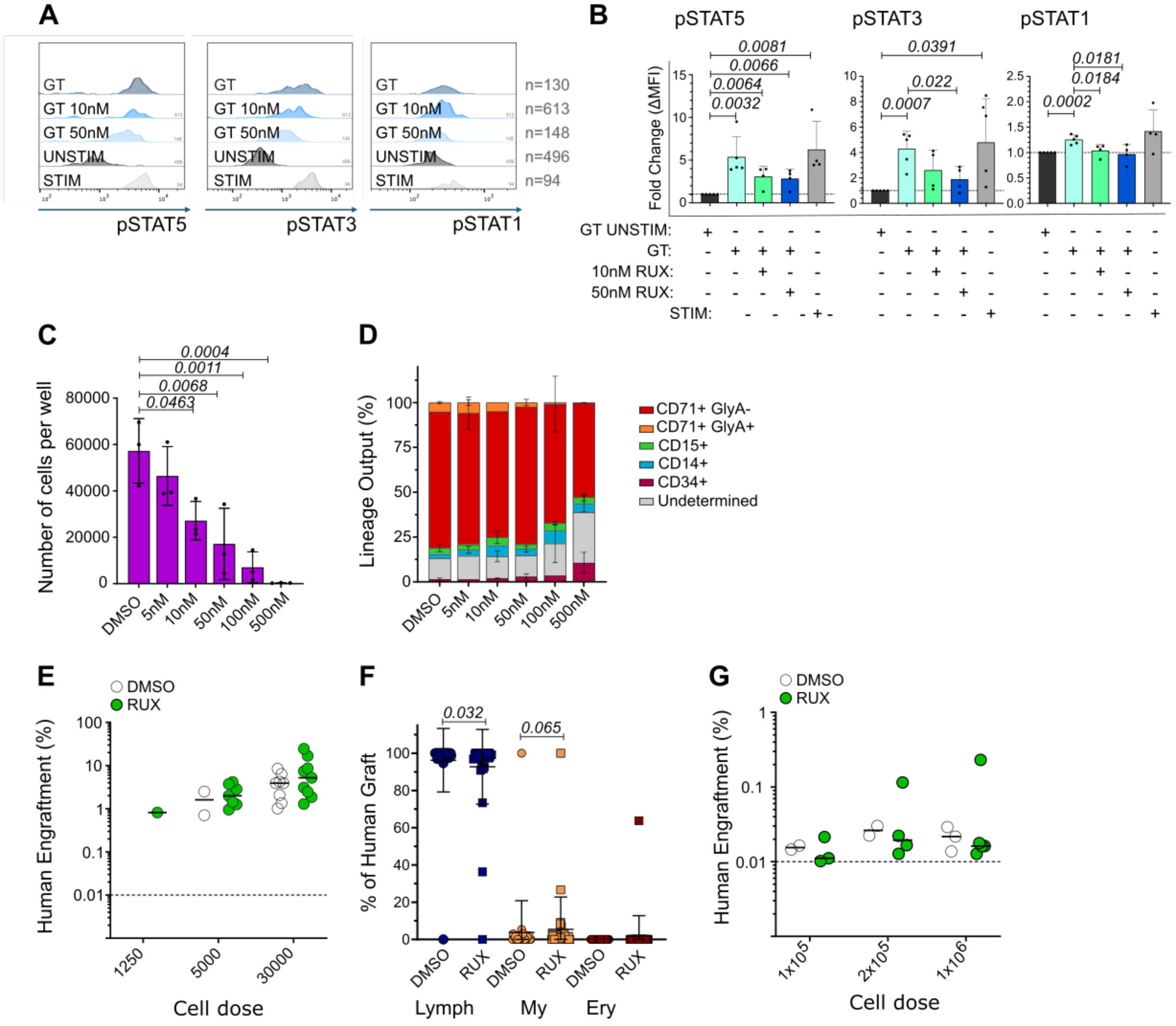

Figure S6: Characterising the effects of RUX treatment on mPB HSCs *in vitro* and *in vivo*

(A-B) Flow cytometry measurement of phosphorylation levels of STAT5, STAT3 and STAT1 in LT-HSCs cultured in modified GT_mPB conditions and indicated RUX doses. n=4 independent biological replicates. **(A)** Representative flow cytometry plots of pSTAT1, pSTAT3 and pSTAT5 measurements. Number of cells in each gate is shown on right. pSTAT1, STAT3 and STAT5 measured in the same reaction. **(B)** MFI for individual pSTAT proteins normalised to GT base media with no cytokine addition (GT UNSTIM). STIM conditions represent GT base media with high concentration of stimulatory cytokines (inc. EPO, IFN-g, IL-6, TPO, SCF and Flt3-L). Mean and SD shown. Unpaired t-test comparisons with $p<0.05$ shown (STAT3: GT UNSTIM vs 10nM RUX $p=0.0761$; GT UNSTIM vs 50nM RUX $p=0.0525$. STAT1: GT UNSTIM vs STIM $p=0.0541$).

(C-D) 250 mPB LT-HSC ($CD19^{-}CD34^{+}CD38^{-}CD45RA^{-}CD90^{+}CD49f^{+}$) cultured for 14 days in MEM conditions with DMSO or the indicated doses of RUX. **(C)** Bar plots showing the number of viable cells at day 14. Bars show mean ± SD. Two-way ANOVA performed with multiple

comparisons and *$p<0.05$* shown. Data-points represents the mean of technical replicates from a single donor. **(D)** Stacked bar plots show the frequency of indicated cell types in culture with RUX. Two-way ANOVA with Sidak's multiple comparisons performed. All comparisons n/s. Gating strategy shown in **Fig.S8**. Mean ± SD shown. n=3 independent mPB donors.

(E) Graft size (% of human $CD45^{++}$) in peripheral blood at 8-week post transplantation of mPB $CD34^{+}CD38^{-}$ cells after GT protocol culture for 62h including LV with DMSO/ RUX (10nM) (n=6 biological repeats; n=84 mice). Only engrafted mice shown (n=27 mice; n=17 RUX mice and n=10 DMSO mice). Dotted line: threshold of engraftment ($CD45^{++\ +} \geq 0.01$ % and at least 30 cells recorded. Two-way ANOVA with Sidak's multiple comparisons performed (5000 cells DMSO vs 5000 cells RUX p=0.265; 30000 cells DMSO vs 30000 cells RUX p=0.265).

(F) Lineage output of human graft at 18 weeks post transplantation in BM of mice transplanted with DMSO/RUX treated LT-HSCs cultured for 62h in GT_mPB system (n=6 biological experiments; n=90 mice total). Only engrafted mice shown (n=68 mice; n=34 DMSO mice and n=34 RUX mice). Mann Whitney U test performed (Lymphoid DMSO vs RUX p=0.053; Myeloid DMSO vs RUX p=0.115; Erythroid DMSO vs RUX p>0.9).

(G) Graft size (% of human $CD45^{++}$ & $GlyA^{+}$) in BM of secondary transplanted mice at 8-week post transplantation of BM from primary mice (n=1 experiment; n=35 mice total). Only engrafted mice shown (n=19 mice; n=7 DMSO n=12 RUX). Dotted line: threshold of engraftment ($CD45^{++\ +} \geq 0.01$ % and at least 30 cells recorded).

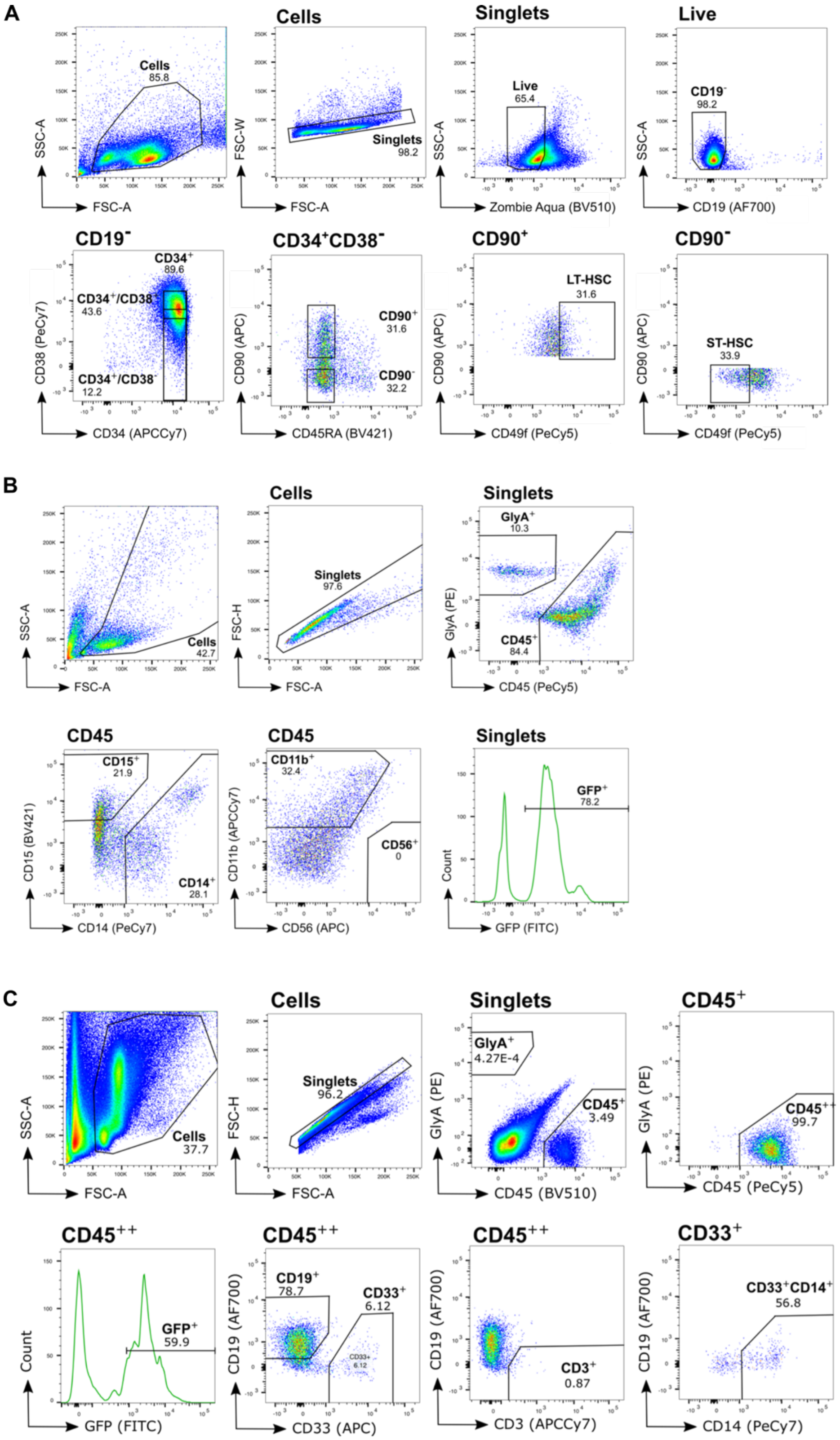
A
Cells
Singlets
Live
Cells
85.8
SSC-A
FSC-A
FSC-W
Singlets
98.2
Live
65.4
Zombie Aqua (BV510)
CD19⁻
98.2
CD19 (AF700)
CD19⁻
CD34⁺CD38⁻
CD90⁺
CD90⁻
CD34⁺
89.6
CD34⁺/CD38⁺
43.6
CD34⁺/CD38⁻
12.2
CD38 (PeCy7)
CD34 (APCCy7)
CD90⁺
31.6
CD90⁻
32.2
CD90 (APC)
CD45RA (BV421)
LT-HSC
31.6
CD49f (PeCy5)
ST-HSC
33.9
B
Cells
Singlets
Cells
42.7
Singlets
97.6
FSC-H
GlyA⁺
10.3
CD45⁺
84.4
GlyA (PE)
CD45 (PeCy5)
CD45
CD45
Singlets
CD15⁺
21.9
CD14⁺
28.1
CD15 (BV421)
CD14 (PeCy7)
CD11b⁺
32.4
CD56⁺
0
CD11b (APCCy7)
CD56 (APC)
GFP⁺
78.2
Count
GFP (FITC)
C
Cells
Singlets
CD45⁺
Cells
37.7
Singlets
96.2
GlyA⁺
4.27E-4
CD45⁺
3.49
CD45 (BV510)
CD45⁺⁺
99.7
CD45 (PeCy5)
CD45⁺⁺
CD45⁺⁺
CD45⁺⁺
CD33⁺
GFP⁺
59.9
CD19⁺
78.7
CD33⁺
6.12
CD19 (AF700)
CD33 (APC)
CD3⁺
0.87
CD3 (APCCy7)
CD33⁺CD14⁺
56.8

Fig. S7. Representative gates for LT-HSC isolation, colony generation from single LT-HSCs and long-term *in vivo* grafts generated from transplanted HSCs

(A) Representative gating strategy for isolation of LT-HSC. The example is here is from a 0h mPB $CD34^{+}$ enriched donor sample. LT-HSC: $Zombie^{-}CD19^{-}CD34^{+}CD38^{-}CD90^{+}CD49f^{+}$ (top 30% of CD90 and CD49f expression). The same strategy was used for CB LT-HSCs.

(B) Representative gating strategy for determination of colony size and lineage output from single cell MEM assay. Representative gating of a colony arising from a single sorted mPB LT-HSC after 21 days culture in MEM media. A true colony was determined if >30 cells are observed in each of the $CD45^{+}$ and $GlyA^{+}$ gates. Colonies were assigned to specific lineages if >30 cells were found in the following gates: myeloid: $CD45^{+}CD11b^{+}$; monocyte: $CD45^{+}CD14^{+}$; granulocyte: $CD45^{+}CD15^{+}$; lymphoid (NK only): $CD45^{+}CD11b^{-}CD56^{+}$. Undifferentiated colonies were determined as having >30 cells in ($CD45^{+}$ & $GlyA^{+}$) gates but <30 cells in all the other lineages.

(C) Representative gating strategy for analysis of mouse BM after primary transplantation (18-20 weeks). The example here is from a mouse injected with mPB $CD34^{+}CD38^{-}$ cells cultured in GT conditions (62h) and transduced with a LV containing GFP. Erythroid (Ery) cells were identified as $CD45^{-}GlyA^{+}$. Myeloid (My) cells were identified as $CD45^{++}CD33^{+}$. Lymphoid (Lym) cells were identified as $CD45^{++}CD19^{+}$. The same gating strategy was used for CB experiments and all secondary transplantation experiments.

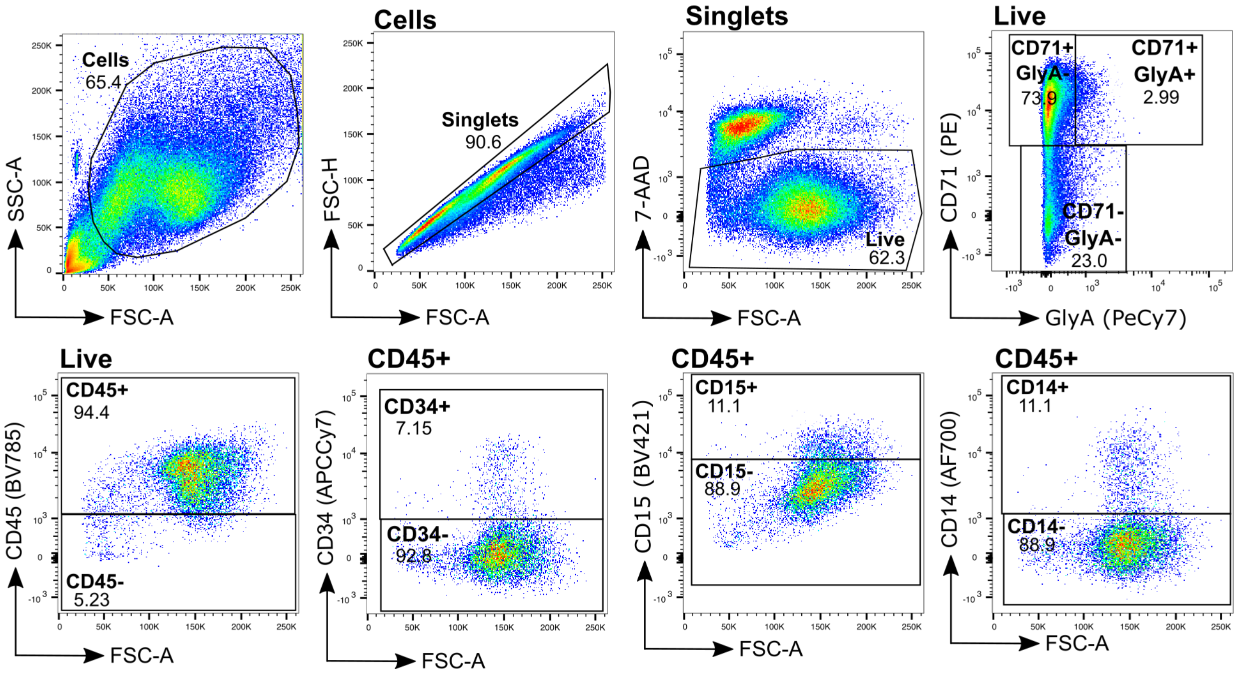

Fig. S8. Representative gates for 14-day differentiation of LT-HSCs

Representative gating of cells arising from 250 sorted mPB LT-HSCs cultured in DMSO or RUX doses (5-500nM) after 14 days culture in MEM media. Absolute cell number determined from Live (7-AAD$^-$). Erythroid progenitors determined as: CD71$^+$GlyA$^-$ and CD71$^+$GlyA$^+$ cells. Myeloid cells determined as: CD45$^+$CD14$^+$ (monocyte) and CD45$^+$CD15$^+$ (granulocyte), CD45$^+$CD34$^+$ (HSPCs) and CD45$^-$ (Undetermined).

Table S1. Experimental details and engraftment levels for mice used in primary transplantation experiments

Culture conditions	Cell type	Hours of culture (h)	Cell Dose	Number of tested mice	Number of engrafted mice	Number of experiments	LTRC frequency (ELDA estimate and 95% CI)	% LTRC (ELDA estimate and 95% CI)
GT_mPB	$CD34^+$ $CD38^-$	0	50	6	1	2	1 in 939 (477-1846)	0.106 (0.054-0.210)
			250	7	5			
			1250	9	7			
			5000	5	4			
			8000	3	3			
		6	50	10	3	2	1 in 1211 (518-2829)	0.083 (0.035-0.193)
			1250	6	2			
			5000	5	5			
		24	50	8	1	2	1 in 3371 (1614-7037)	0.030 (0.014-0.062)
			1250	8	4			
			5000	7	4			
		62	50	7	0	2	1 in 2510 (1239-5084)	0.040 (0.020-0.081)
			1250	7	3			
			5000	5	4			
			8000	4	4			
EXPER_CB	LT-HSC	0	5	14	6	2	1 in 14.8 (8.1 – 26.8)	6.8 (3.7–12.3)
			25	10	7			
			100	7	7			
		6	5	8	4	2	1 in 15.2 (6.7 – 34.2)	6.6 (2.9-14.8)
			25	5	3			
			100	6	6			
		24	10	8	0	3	1 in 80.6 (43.3 – 150)	1.2 (0.7-2.3)
			50	11	5			
			300	12	12			
		72	50	13	4	5	1 in 293.7 (184 – 468)	0.34 (0.21-0.54)
			300	12	9			
			700	15	12			

Table S2: Engraftment data for primary Limiting Dilution Assays reported in this study comparing culture duration in untreated conditions.

Number of mice injected and number of mice engrafted for each of the doses tested in primary limiting dilution experiments. Mice were considered engrafted if human cells ($hCD45^{++}$ & $GlyA^+$) were ≥30 cells and represent ≥0.01% of Singlets, and if ≥20 cells were present in any lineage determination gate. Engraftment measurements for all primary mice are reported in **Table S1**. EXPER_CB mice were considered engrafted if engrafted thresholds were met in either the injected femur or other bone marrow harvested from hind legs. LTRC: Long Term Repopulating Cell.

Table S3. DESeq2 output for the comparison of 0h-early and 0h-late LT-HSC subsets

Table S4. DESeq2 output for all pairwise comparisons of timepoints in the scRNA-seq time course of LT-HSCs cultured in EXPER_CB system

The specific timepoints being compared are indicated in the name of each tab.

Table S5. Lists of "ex vivo modulated genes" defined in this study

Tab "ex_vivo_mod_genes_10010_EXPER" reports the union of all differentially expressed genes (by DESeq2 FDR<0.05) between any 2 pairwise comparisons in Integration 1 (0h, 6h, 24h, 72h; n=10,010 genes).

Tab "ex_vivo_mod_genes+PD_EXPER" reports the union of the 10,010 genes and of the genes differentially expressed between 0h vs 72h PD, 72h PD vs 72h UNTR, 0 vs 24h PD and 24h PD vs 2 h UNTR (n=10,903 genes).

Tab "ex_vivo_mod_genes+PD_GT" reports the union of all differentially expressed genes (by DESeq2 FDR<0.05) between any 2 pairwise comparisons in Dataset 2 (0h, 62h UNTR, 62h PD; n=5,469 genes).

Table S6. List of genes and GSVA pathways in each pattern identified by DEGpattern analysis

Table S7. List of maximally variable genes at each time point of scRNA-seq timecourse, with associated Reactome pathways analysis

These results pertain to the BASiCS variability analysis.

Tab "Maximally_variable_genes_time" reports the maximally variable genes corresponding to each timepoint. The other tabs show the full output of Reactome pathway analysis from these lists.

Table S8. DESeq2 output and Reactome pathway analysis for comparisons of untreated and PD treated conditions

Tabs "24hPD_vs24hUNTR_EXPER", "72hPD_vs72hUNTR_EXPER", and "62hPD_vs_ 62hUNTR_GT" tabs report the DEseq2 output for the analysis of differentially expressed genes between the conditions indicated in the tab name. The other tabs show the full output of Reactome pathway analysis from the differentially expressed genes (FDR < 0.05) in each of the lists.

Culture conditions	Cell type	Hours of culture (h) and Condition	Cell Dose	Number of tested mice	Number of engrafted mice	Number of experiments	LTRC frequency (ELDA estimate and 95% CI)	% LTRC (ELDA estimate and 95% CI)
**GT_mPB**	CD34^{+} CD38^{-}	62 PD	8000	4	3	3		
			25000	11	11			
			55000	13	12			
		62 UNTR	8000	4	4	3		
			25000	13	12			
			55000	11	9			
**EXPER_ CB**	LT-HSC	24 PD	10	7	1	3	1 in 53.9 (27.3 – 106)	1.9 (0.9 – 3.7)
			50	10	6			
			300	13	13			
		24 UNTR	10	8	0	3	1 in 80.6 (43.3 – 150)	1.2 (0.7 - 2.3)
			50	11	5			
			300	12	12			
		72 PD	50	12	4	5	1 in 230 (146 – 361)	0.43 (0.28 – 0.68)
			300	15	14			
			700	15	12			
		72 UNTR	50	13	4	5	1 in 294 (184 – 468)	0.34 (0.21-0.54)
			300	12	9			
			700	15	12			

Table S9: Engraftment data for primary Limiting Dilution Assays reported in this study comparing UNTR/PD treated conditions
Number of mice injected and number of mice engrafted for each of the doses and conditions tested in primary limiting dilution experiments. Mice were considered engrafted if human cells (hCD45^{++} & GlyA^{+}) were ≥30 cells and represent ≥0.01% of Singlets, and if ≥20 cells were present in any lineage determination gate. Engraftment measurements for all primary mice are reported in **Table S1**. EXPER_CB mice were considered engrafted if engrafted thresholds were met in either the injected femur or other bone marrow harvested from hind legs. UNTR: Untreated; PD: Palbociclib; LTRC: Long Term Repopulating Cell.

Culture conditions	Cell type	Hours of culture (h) and Condition	Cell Dose	Number of tested mice	Number of engrafted mice	Number of experiments	LTRC Frequency (ELDA estimate and 95% CI)	% LTRC (ELDA estimate and 95% CI)
**GT_mPB**	Whole BM	62 PD	100000	4	3	1	1 in 622437 (213407 - 1815440)	1.61E-04 (4.69E-04 - 5.51E-05)
			200000	3	1			
			1000000	3	1			
		62 UNTR	100000	4	1	1	1 in 557781 (212307 - 1465425)	1.79E-04 (4.71E-04 - 6.82E-05)
			200000	3	3			
			1000000	4	2			
**EXPER_CB**	CD45++	72 PD	42500	4	1	1	1 in 339688 (119228 - 967794)	2.94E-04 (8.39E-04 - 1.03E-04)
			170000	3	0			
			680000	3	3			
		72 UNTR	42500	4	0	1	1 in 1129479 (270729 - 4712177)	8.85E-05 (3.69E-04 - 2.12E-05)
			170000	3	1			
			680000	3	1			

Table S10: Engraftment data for secondary Limiting Dilution Assays reported in this study comparing UNTR/PD treated conditions

Number of mice injected and number of mice engrafted for each of the doses and conditions tested in secondary limiting dilution experiments. GT mice were transplanted with counted, unsorted BM isolated from primary recipients. EXPER mice were transplanted with $CD45^{++}$ cells isolated from primary recipients by flow cytometry. Mice were considered engrafted if human cells ($hCD45^{++}$ & $GlyA^{+}$) were ≥30 cells and represent ≥0.01% of Singlets, and if ≥20 cells were present in any lineage determination gate. EXPER_CB mice were considered engrafted if engrafted thresholds were met in either the injected femur or other bone marrow harvested from hind legs. UNTR: Untreated; PD: Palbociclib; LTRC: Long Term Repopulating Cell.

pSTAT	Donor	Metric	GT UNSTIM	GT	GT + RUX (10nM)	GT + RUX (50nM)	STIM
pSTAT1	Donor1	MFI	206	273	261	223	287
pSTAT1	Donor2	MFI	205	288	281	221	342
pSTAT1	Donor3	MFI	251	329	247	254	333
pSTAT1	Donor4	MFI	314	303	270	260	342
pSTAT1	Donor 1-4	SD	51.36	23.88	14.38	20.37	26.34
pSTAT3	Donor1	MFI	458	1779	1490	1012	2277
pSTAT3	Donor2	MFI	416	2033	1757	1046	2910
pSTAT3	Donor3	MFI	381	2334	1660	1131	3349
pSTAT3	Donor4	MFI	387	2223	1559	1243	3858
pSTAT3	Donor 1-4	SD	35.16	243.02	116.82	102.98	670.82
pSTAT5	Donor1	MFI	1090	2867	3020	2519	2884
pSTAT5	Donor2	MFI	784	3497	3296	2666	3791
pSTAT5	Donor3	MFI	568	3230	2852	2530	3520
pSTAT5	Donor4	MFI	762	4592	2824	3117	3809
pSTAT5	Donor 1-4	SD	215.73	743.28	216.70	280.74	432.05

Table S11: pSTAT measurement by flow cytometry. Raw MFI data on Live gate reported per donor. Mean Fluorescence Intensity (MFI).

Table S12: raw data and full statistics of all serial replating colony assays performed in this study

Each tab represent a series of serial replating experiments, with raw colony counts. Glmer analysis fitting raw colony counts (averaged from technical duplicates and rounded up). Tukey corrected p-values for all pairwise comparisons of EM means shown in each tab. Donors 1 to 6 are the same individuals across the RUX_0h, RUX_CASi and RUX_UM171_TPO tabs.

Culture conditions	Cell type	Hours of culture (h) and Condition	Cell Dose	Number of tested mice	Number of engrafted mice	Number of experiments	LTRC Frequency (ELDA estimate and 95% CI)	% LTRC (ELDA estimate and 95% CI)
GT_mPB	$CD34^+$ $CD38^-$	62 RUX	50	9	1	6	1 in 1208 (652 – 2240)	0.08 (0.15 – 0.04)
			1250	8	4			
			5000	18	18			
			30000	11	11			
		62 DMSO	50	8	3	6	1 in 1637 (933 – 2872)	0.06 (0.11 – 0.03)
			1250	8	5			
			5000	17	15			
			30000	11	11			

Table S13: Engraftment data for primary Limiting Dilution Assays reported in this study comparing DMSO/RUX treated conditions.
Number of mice injected and number of mice engrafted for each of the doses and conditions tested in primary limiting dilution experiments. Mice were considered engrafted if human cells ($hCD45^{++}$ & $GlyA^+$) were ≥30 cells and represent ≥0.01% of Singlets, and if ≥20 cells were present in any lineage determination gate. Engraftment measurements for all primary mice are reported in **Table S1.** RUX: Ruxolitinib; LTRC: Long Term Repopulating Cell.

Culture conditions	Cell type	Hours of culture (h) and Condition	Cell Dose	Number of tested mice	Number of engrafted mice	Number of experiments	LTRC Frequency (ELDA estimate and 95% CI)	% LTRC (ELDA estimate and 95% CI)
GT_mPB	Whole BM	62 RUX	100000	7	3	1	1 in 177778 (85446 – 369885)	5.62E-04 (1.17E-03 – 2.70E-04)
			200000	6	4			
			1000000	5	5			
		62 DMSO	100000	7	2	1	1 in 518463 (227327 – 1182456)	1.93E-04 (4.40E-04 – 8.46E-05)
			200000	6	2			
			1000000	4	3			

Table S14: Engraftment data for secondary Limiting Dilution Assays reported in this study comparing DMSO/RUX treated conditions.
Number of mice injected and number of mice engrafted for each of the doses and conditions tested in secondary limiting dilution experiments. Mice were considered engrafted if human cells (hCD45^{++} & GlyA^{+}) were ≥30 cells and represent ≥0.01% of Singlets, and if ≥20 cells were present in any lineage determination gate. RUX: Ruxolitinib; LTRC: Long Term Repopulating Cell.

**Antibody**	**Manufacturer**	**Cat. #**	**Location used**
Rat monoclonal anti-CD49f, PeCy5, clone GoH3	BD	551129	Cambridge
Mouse monoclonal anti-CD38, PeCy7, clone HIT2	Biolegend	303516	Cambridge
Mouse monoclonal anti-CD90, clone 5E10	BD	559869	Cambridge
Mouse monoclonal anti- CD19, AlexaF700, clone HIB19	Biolegend	302226	Cambridge
Mouse monoclonal anti-CD34, APCCy7, clone 581	Biolegend	343514	Cambridge
Mouse monoclonal anti-CD45RA, BV421, clone HI100	Biolegend	304129	Cambridge
Mouse monoclonal anti-GlyA, PE, clone HIR2	BD	340947	Cambridge
Mouse monoclonal anti-CD45, PECy5, clone HI30	Biolegend	304010	Cambridge
Mouse monoclonal anti-CD14, PECy7, clone M5E2	Biolegend	301814	Cambridge
Mouse monoclonal anti-CD56, APC, clone HCD56	Biolegend	318310	Cambridge
Mouse monoclonal CD11b, APCCy7, clone ICRF44	Biolegend	301342	Cambridge
Mouse monoclonal anti-CD15, BV421, clone MC-48	Biolegend	125614	Cambridge
Mouse monoclonal anti CD33, APC, clone P67.6	BD	345800	Cambridge
Mouse monoclonal anti-CD3, APCCy7, clone HIT3a	Biolegend	300318	Cambridge
Mouse monoclonal anti-CD45, BV510, clone HI30	Biolegend	304036	Cambridge
Rabbit monoclonal Phospho-Rb, Alexa Fluor 647	Cell Signalling Technologies	8974S	Cambridge
Mouse monoclonal anti-Ki67, FITC	BD	556026	Cambridge
Mouse monoclonal anti-CD10, BV605, clone HI10a	BD	562978	Cambridge
Mouse monoclonal anti-CD15, BV421, clone W6D3	Biolegend	323039	Cambridge
Mouse monoclonal anti-CD45, BV78,5 clone, HI30	Biolegend	304027	Cambridge
Mouse monoclonal anti-GlyA, PECy7, clone HI264	Biolegend	349111	Cambridge
Mouse monoclonal anti-CD45RA, FITC, clone HI100	BD	563839	Cambridge
Mouse monoclonal anti-CD71, PE, clone RI7217	Biolegend	113807	Cambridge
Mouse monoclonal anti-CD14/AF700 clone M5E2	Biolegend	301822	Cambridge
Mouse monoclonal anti-GlyA, PE, clone 11E4B-7-6	Beckman Coulter	IM2211U	Toronto
Mouse monoclonal anti-CD14, PECy5.5, clone MΦP-9	BD	B62209	Toronto
Mouse monoclonal anti-CD33, APC, clone P67.	BD	340474	Toronto
Mouse monoclonal anti-CD19, AF700, clone HIB19	Biolegend	55792	Toronto
Mouse monoclonal anti-CD3, APCCy7, clone SK7	BD	341090	Toronto
Mouse monoclonal anti-CD45, BV450, clone HI30	BD	560367	Toronto
Mouse monoclonal anti-CD45, VioBright B515, clone REA737	Miltenyi	130-130-165	Toronto

Table S15: Antibodies used in this study.

This study used the antibodies in the table in the panels described below and referred to in the relevant methods sections:

Panel A: CD49f/PECy5 (clone GoH3, 1 in 100), CD38/PECy7 (clone HIT2, 1 in 100), CD90/APC (clone 5E10, 1 in 100), CD19/AlexaF700 (clone HIB19, 1 in 300), CD34/APCCy7 (clone 581, , 1 in 100) and CD45RA/BV421 (clone HI100, 1 in 100).

Panel B: GlyA/PE (clone HIR2, 1 in 1,000), CD45/PECy5 (clone HI30, 1 in 300), CD14/PECy7 (clone M5E2, 1 in 1000), CD56/APC (clone HCD56, 1 in 200), CD11b/APCCy7 (clone ICRF44, 1 in 300) and CD15/BV421 (clone MC-480, 1 in 200).

Panel C: GlyA/PE (clone HIR2, 1 in 1000), CD45/PECy5 (clone HI30, 1 in 300), CD14/PECy7 (clone M5E2, 1 in 1,000), CD33/APC (clone P67.6, 1 in 200), CD19/AlexaF700 (clone HIB19, 1 in 300), CD3/APCCy7 (clone HIT3a, 1 in 100) and CD45/BV510 (clone HI30, 1 in 500).

Panel D: CD45/FITC (clone 2D1, 1 in 100) CD45/PeCy5 (clone J33, 1 in 200), CD34/APCCy7 (clone 581, 1 in 200) and CD38/PeCy7 (clone HB7, 1 in 200).

Panel E: GlyA/PE (clone 11E4B-7-6), CD14/PeCy5-5 (clone MΦP-9), CD33/APC (clone P67.6, 1 in 100), CD19/AF700 (Clone HIB19), CD3/APCCy7 (clone SK7), CD45/BV450 (clone HI30), CD45/Vio Bright B515 (clone REA737).

For Live/Dead discrimination Zombie Aqua (cat. no. 423101, 1 in 2,000) was included in Panel A and Sytox Blue**** (cat no. S34857, 1 in 2,000) was included in Panel D.

Panel F: CD10/BV605 (clone HI10a), CD15/BV421 (clone W6D3), CD45/BV785 (clone HI30), CD34/APCCy7 (clone 581), GlyA/PECy7 (clone HI264), CD45RA/FITC (clone, HI100), CD71/PE (clone, RI7217), CD14/AF700 (clone M5E2)

Batch number	Time-point	Sorted	After QC
1	0h	72	29
	6h	72	49
	24h	72	28
	72h UNTR	72	42
	72h PD	72	27
2	0h	94	56
	6h	94	85
	24h UNTR	95	58
	24h PD	95	80
	72h UNTR	95	82

Table S16. Number of single cells before and after QC for Dataset 1 (EXPER_CB conditions).

Batch Number	Cell Type	Before QC	After QC	After outlier removal
Batch 1	0h	96	83	82
	62h UNTR	84	76	76
	62h PD	0	0	0
Batch 2	0h	66	50	50
	62h UNTR	48	39	39
	62h PD	48	38	38
Batch 3	0h	62	42	42
	62h UNTR	24	16	16
	62h PD	24	12	12
Batch 4	0h	96	44	44
	62h UNTR	48	12	12
	62h PD	44	7	7

Table S17. Number of single cells before and after QC for Dataset 2 (GT_mPB conditions).

	Dataset 1		Dataset 2			
**QC thresholds**	**Batch 1**	**Batch 2**	**Batch 1**	**Batch 2**	**Batch 3**	**Batch 4**
Number of Mapped reads	> 330,000	> 190,000	NA	NA	NA	NA
Number of nuclear genes	> 276,500	> 180,000	> 158,489	> 158,489	> 15,000	> 100,000
Ratio of genes to total number of reads	> 0.342	> 0.2	> 0.3	> 0.3	> 0.1	> 0.2
Number of genes with 10 reads per million	> 2100	> 1000	NA	> 1000	> 1000	> 1000
Ratio of nuclear genes to number of mapped reads	> 0.818	NA	NA	NA	NA	NA
Ratio of mitochondrial genes to number of mitochondrial and nuclear genes	NA	< 0.2	< 0.2	< 0.2	< 0.2	< 0.2
Ratio of ERCC to number of mapped reads	NA	< 0.2	< 0.4	< 0.4	< 0.2	< 0.2
Ratio of ‘QC_no_feature’ to total number of readsw	NA	< 0.36	NA	NA	NA	NA

Table S18. Quality control thresholds (QC) for scRNA-seq experiments.

QC thresholds used for filtering “good quality” single cells in each of the independent scRNA-seq experiments. NA: not applied.

References

1. Notta F, Doulatov S, Laurenti E, et al. Isolation of Single Human Hematopoietic Stem Cells Capable of Long-Term Multilineage Engraftment. *Science*. 2011;333(6039):218–221.
2. Picelli S, Faridani OR, Björklund ÅK, et al. Full-length RNA-seq from single cells using Smart-seq2. *Nature Protocols*. 2014;9(1):171–181.
3. Wu TD, Reeder J, Lawrence M, Becker G, Brauer MJ. GMAP and GSNAP for Genomic Sequence Alignment: Enhancements to Speed, Accuracy, and Functionality. *Methods Mol Biol*. 2016;1418:283–334.
4. Babraham Bioinformatics - FastQC A Quality Control tool for High Throughput Sequence Data. https://www.bioinformatics.babraham.ac.uk/projects/fastqc/
5. Anders S, Pyl PT, Huber W. HTSeq--a Python framework to work with high-throughput sequencing data. *Bioinformatics*. 2015;31(2):166–169.
6. Jawaid W. Gottgens lab single cell RNAseq toolkit. 2017; https://github.com/wjawaid/bglab
7. Hao Y, Hao S, Andersen-Nissen E, et al. Integrated analysis of multimodal single-cell data. *Cell*. 2021;184(13):3573-3587.e29.
8. Cao J, Spielmann M, Qiu X, et al. The single-cell transcriptional landscape of mammalian organogenesis. *Nature*. 2019;566(7745):496–502.
9. SeuratWrappers. 2022; https://github.com/satijalab/seurat-wrappers
10. Wolf FA, Angerer P, Theis FJ. SCANPY : large-scale single-cell gene expression data analysis. *Genome Biology*. 2018;19(1):15.
11. Satija R, Farrell JA, Gennert D, Schier AF, Regev A. Spatial reconstruction of single-cell gene expression data. *Nat Biotechnol*. 2015;33(5):495–502.
12. Polański K, Young MD, Miao Z, et al. BBKNN: fast batch alignment of single cell transcriptomes. *Bioinformatics*. 2020;36(3):964–965.
13. Love MI, Huber W, Anders S. Moderated estimation of fold change and dispersion for RNA-seq data with DESeq2. *Genome Biology*. 2014;15:550.
14. Ritchie ME, Phipson B, Wu D, et al. limma powers differential expression analyses for RNA-sequencing and microarray studies. *Nucleic Acids Research*. 2015;43(7):e47.
15. Subramanian A, Tamayo P, Mootha VK, et al. Gene set enrichment analysis: a knowledge-based approach for interpreting genome-wide expression profiles. *Proc. Natl. Acad. Sci. U.S.A.* 2005;102(43):15545–15550.
16. Hänzelmann S, Castelo R, Guinney J. GSVA: gene set variation analysis for microarray and RNA-Seq data. *BMC Bioinformatics*. 2013;14(1):7.
17. Belluschi S, Calderbank EF, Ciaurro V, et al. Myelo-lymphoid lineage restriction occurs in the human haematopoietic stem cell compartment before lymphoid-primed multipotent progenitors. *Nat Commun*. 2018;9(1):4100.
18. Zhang YW, Mess J, Aizarani N, et al. Hyaluronic acid–GPRC5C signalling promotes dormancy in haematopoietic stem cells. *Nat Cell Biol*. 2022;24(7):1038–1048.
19. Patano L. degPatterns. Available at: https://rdrr.io/github/lpantano/DEGreport/man/degPatterns.html. 2017.
20. Liu B, Li C, Li Z, et al. An entropy-based metric for assessing the purity of single cell populations. *Nature Communications*. 2020;11(1):3155.
21. Eling N, Richard AC, Richardson S, Marioni JC, Vallejos CA. Correcting the Mean-Variance Dependency for Differential Variability Testing Using Single-Cell RNA Sequencing Data. *Cell Systems*. 2018;7(3):284-294.e12.

22. Kaufmann KB, Zeng AGX, Coyaud E, et al. A latent subset of human hematopoietic stem cells resists regenerative stress to preserve stemness. *Nat Immunol*. 2021;22(6):723–734.
23. Knapp DJHF, Hammond CA, Hui T, et al. Single-cell analysis identifies a CD33+ subset of human cord blood cells with high regenerative potential. *Nature Cell Biology*. 2018;20(6):710–720.
24. Sommarin MNE, Dhapola P, Safi F, et al. Single-Cell Multiomics Reveals Distinct Cell States at the Top of the Human Hematopoietic Hierarchy. *bioRxiv*. 2021;2021.04.01.437998.
25. Zhang YW, Mess J, Aizarani N, et al. Hyaluronic Acid–GPRC5C Signalling Promotes Dormancy in Hematopoietic Stem Cells. *Nature Cell Biology*. 2022;
